# Drug and siRNA screens identify ROCK2 as a therapeutic target for ciliopathies

**DOI:** 10.1038/s43856-025-00847-1

**Published:** 2025-04-19

**Authors:** Claire E. L. Smith, Andrew J. Streets, Alice V. R. Lake, Subaashini Natarajan, Sunayna K. Best, Katarzyna Szymanska, Magdalena Karwatka, Thomas Stevenson, Rachel Trowbridge, Gary Grant, Sushma N. Grellscheid, Richard Foster, Ciaran G. Morrison, Georgia Mavria, Jacquelyn Bond, Albert C. M. Ong, Colin A. Johnson

**Affiliations:** 1 Division of Molecular Medicine, Leeds Institute of Medical Research, Faculty of Medicine and Health, University of Leeds, Leeds, UK; 2 Academic Nephrology Unit, Division of Clinical Medicine, School of Medicine and Population Health, Faculty of Health, University of Sheffield, Sheffield, UK; 3 Computational Biology Unit, Department of Biosciences, University of Bergen, Bergen, Norway; 4 School of Molecular & Cellular Biology & Astbury Centre for Structural Molecular Biology, Faculty of Biological Sciences, University of Leeds, Leeds, UK; 5 Department of Biosciences, University of Durham, Durham, UK; 6 School of Chemistry, University of Leeds, Leeds, UK; 7 Centre for Chromosome Biology, School of Biological and Chemical Sciences, University of Galway, Galway, Ireland; 8 St James’s Campus Infrastructure Flow Cytometry and Imaging Facility, Faculty of Medicine and Health, University of Leeds, St James’s University Hospital, Leeds, UK

**Keywords:** High-throughput screening, RNAi, Polycystic kidney disease

## Abstract

**Background:**

Primary cilia mediate vertebrate development and growth factor signalling. Defects in primary cilia cause inherited developmental conditions termed ciliopathies. Ciliopathies often present with cystic kidney disease, a major cause of early renal failure. Currently, only one drug, Tolvaptan, is licensed to slow the decline of renal function for the ciliopathy polycystic kidney disease. Novel therapeutic interventions are needed.

**Methods:**

We screened clinical development compounds to identify those that reversed cilia loss due to siRNA knockdown. In parallel, we undertook a whole genome siRNA-based reverse genetics phenotypic screen to identify positive modulators of cilia formation.

**Results:**

Using a clinical development compound screen, we identify fasudil hydrochloride. Fasudil is a generic, off-patent drug that is a potent, broadly selective Rho-associated coiled-coil-containing protein kinase (ROCK) inhibitor. In parallel, the siRNA screen identifies *ROCK2* and we demonstrate that ROCK2 is a key mediator of cilium formation and function through its possible effects on actin cytoskeleton remodelling.

**Conclusions:**

Our results indicate that specific ROCK2 inhibitors (e.g. belumosudil) could be repurposed for cystic kidney disease treatment. We propose that ROCK2 inhibition represents a novel, disease-modifying therapeutic approach for heterogeneous ciliopathies.

## Plain language summary

Primary cilia are antennae-like structures on cells that are important for early development and healthy cell function. Defects in primary cilia can cause inherited diseases called ciliopathies. Ciliopathies often cause fluid-filled sacs, called cysts, that are a major cause of kidney disease and failure. There is currently one drug licensed to slow kidney disease progression, but it is poorly tolerated in patients. Therefore, new drugs are needed. In this study, we used screening assays to identify potential drugs and their targets that are effective in promoting the formation of primary cilia. Our results identified ROCK2 (Rho-associated coiled-coil-containing protein kinase 2), an inhibitor of protein signalling, as a key mediator of cilium function. These findings suggest that drugs that specifically target ROCK2 could be a potential treatment option for cystic kidney disease.

## Abstract (web summary)

Smith et al. use clinical development screen and whole genome siRNA-reverse genetics phenotypic screen to identify ROCK2, as a modulator of cilia formation and function via its effects on actin cytoskeleton remodelling. Repurposing ROCK2 is a viable treatment for ciliopathies, for which a limited therapeutic option is available.

## Introduction

Primary cilia are microtubule-based organelles extending from the apical surface of most mammalian cells. They act as cellular antennae in vertebrates that receive and integrate mechanical and chemical signals from the extracellular environment, serving diverse roles in chemo-, mechano- and photo-sensation^1^. Cilia mediate many vertebrate developmental and growth factor signalling pathways, including those activated by Sonic Hedgehog (Shh), PDGF and Wnt ligands^2–4^. Ciliogenesis occurs during G_1_ and is initiated by the docking of the mother centriole at the apical cell surface via its subdistal appendages, prior to formation of the basal body, trafficking of ciliary vesicles and subsequent extension of the ciliary axoneme^5^. Defects in primary cilia are associated with heterogeneous but comparatively common Mendelian inherited conditions known as the ciliopathies^6^, with an overall estimated prevalence of 1 in 2000^7^. Affected children and adults commonly present with cystic kidney disease and have other clinical features such as retinal degeneration. Cystic kidney disease, associated with ciliopathies such as autosomal dominant polycystic kidney disease (ADPKD), Joubert syndrome and nephronophthisis, is a major cause of renal failure within the first two decades of life. The incidence of these ciliopathies range between 200 and 300 live births per annum in the UK^7^, and we estimate that about 300 patients in the UK newly require renal replacement therapy (dialysis and transplantation) annually that can be attributed to cystic kidney disease leading to kidney failure.

Tolvaptan, a selective vasopressin V2 receptor antagonist, slows the decline in renal function for ADPKD^8,9^. However, common (11–13%) side effects of Tolvaptan include polyuria, increased thirst, pollakiuria and xerostomia^10,11^, which mean that many patients do not tolerate treatment. Furthermore, there are no preventative treatments or new therapeutic interventions that may modify disease progression or the long-term outlook of ciliopathy patients with other types of cystic kidney disease. There are often several years between diagnosis and end-stage renal failure in the paediatric or juvenile age ranges, creating a window for therapeutic intervention. This has created an acute clinical need for the potential repurposing of existing drugs or the development of novel lead compounds that could treat cystic kidney disease. A recent cellular phenotype-based drug screen identified eupatilin, a plant flavonoid, which rescues ciliogenesis and ciliary transport defects caused by loss of the ciliopathy protein CEP290^12^. However, the advantages of a drug repurposing approach include existing efficacy and safety profiles established in previous clinical trials for other indications. Drug repurposing, therefore, significantly reduces the time and investment required to make the move from the laboratory to the clinic^13^.

Here, we report a series of cellular phenotype-based compound library screens of clinical development compounds for effects on cilia formation and function. We identify fasudil hydrochloride, a potent but broadly selective Rho-associated coiled-coil-containing protein kinase (ROCK) inhibitor, as the top drug hit. In a parallel whole genome siRNA-based reverse genetics phenotypic screen of positive modulators of cilia formation, we identify ROCK2 as the target molecule and demonstrate that ROCK2 is a key mediator of cilium formation and function through downstream effects on actin cytoskeleton remodelling. Specific ROCK2 ablation or inhibition has a broad effect in rescuing ciliary function over several ciliopathy disease classes. We propose that ROCK2 inhibition represents a novel, disease-modifying therapeutic approach for heterogeneous ciliopathies. These results indicate that ROCK inhibitors, particularly specific ROCK2 inhibitors such as belumosudil (also known as KD025), could be repurposed for pharmacological intervention in cystic kidney disease.

## Methods

### Ethical approval: human subjects and mice

The cell line OX161/c1 was immortalised from tubule cells taken from an ADPKD human kidney as part of clinical care with informed consent and have been previously described elsewhere^14^.

All experiments in mice were carried out under UK Home Office project licence PP9820851 with local ethical approval by the University of Sheffield.

### Cell lines

Cell lines were sourced from the American Type Culture Collection® (ATCC®) unless otherwise stated. Cells were used for screening between passages 15 and 25. mIMCD3 and hTERT RPE-1 mother cell lines were previously verified using arrayCGH and RNA-sequencing^15^ (Short Read Archive accession numbers SRX1411364, SRX1353143, SRX1411453 and SRX1411451). NIH3T3 Shh-Light II cells were a gift from Frédéric Charron, Montreal Clinical Research Institute. Wild-type (AD2) induced pluripotent stem cells^16^ were a gift from Prof Majlinda Lako, University of Newcastle and were maintained in mTeSR plus media (Stem Cell Technologies). The derivation of the OX161-c1 cell line and growth conditions used have previously been described^14^. Briefly, cystic epithelial cells were prepared from tubular cells isolated from PKD human kidneys removed as part of clinical care. Primary cells were immortalised by transduction with a replication-defective retroviral vector containing the catalytic subunit of human telomerase. Cell lines were maintained by passaging under standard conditions and were tested every 3 months for mycoplasma.

### Antibodies

The following antibodies were obtained commercially: anti-acetylated α-tubulin (clone 6-11B-1, Sigma T6793); anti-ARL13B (Proteintech 17711-1-AP); anti-ARL13B (clone N295B/66, NeuroMab); anti-myosin light chain II (CST 8505); anti-phosphorylated myosin light chain II (Ser19) (CST 3671); phosphorylated myosin light chain II (Thr18/Ser19) (CST 3674); anti-ROCK1 (clone C8F7, CST 4035); anti-ROCK2 (Bethyl A300-047A); anti-IFT88 (Proteintech 13967-1-AP), anti-GAPDH (CST 2118); anti-β-actin (clone AC-15, Abcam Ab6276); anti-Smoothened homologue (Bioss antibodies, bs-2801R), anti-polyglutamylation modification (GT335, Adipogen, AG-20B-0020-C100), anti g-tubulin (Abcam Ab137822), anti-OCT3/4 (R&D Systems AF1759) and anti-SSEA4 (BD Biosciences 560218) preconjugated with Alexa Fluor 555, anti-RPGRIP1L (Proteintech 55160-1-AP), anti-beta actin (AC-15, Sigma A1978), anti-vinculin (Sigma V4505), anti-ezrin (Proteintech 26056-1-AP), anti-ZO-1 (ZO-1-1A12, Invitrogen 339100), anti-E-cadherin (BD Bioscience 610181), anti NPHS1 (R&D Systems AF4269). Mouse monoclonal anti-human CEP164 1F3G10 was purified and validated as described^17^.

### Lectins

Fluorescein lotus lectin was obtained from Vector Labs (FL-1321-2).

### Immunofluorescence imaging

Cells plated on slides or in 96-well plates were fixed using 4% paraformaldehyde (PFA) or using ice-cold methanol followed by permeabilisation using PBS containing 0.1% Triton X-100. Cells were stained for immunofluorescence using standard methods. Cells in plates were stained using anti-ARL13B (1:6000) as a cilia marker. Cells on coverslips were stained with ARL13B (1:2000) or acetylated α-tubulin (1:1000) as cilia markers. Slides were imaged as z stacks using confocal microscopy (Nikon A1R Confocal Laser Scanning Microscope, controlled by the NIS-Elements C software). Confocal images were analysed for cilia length and incidence using FIJI image software^18^ macros. Cilia and nuclei were highlighted as regions of interest (ROI) and were quantified for a total number of each ROI, cilia length and total fluorescence using a custom-written macro (Supplemental Data 3). 96-well plates were stained for MLCII, p-MLCII (Ser19) and pp-MLCII (Thr18/Ser19) (1:50) to image MLCII and its phosphorylated isoforms, and ARL13B (1:2000) to image cilia. Plates were imaged at 40× using a high-throughput microscope (PerkinElmer Operetta® microscope controlled using Harmony® high-content analysis software). Image data were analysed using the Columbus™ Image Data Storage and Analysis system (PerkinElmer).

Changes in acto-myosin contractions were determined by high-content imaging and analysis of phosphorylated MLCII levels, which reflect the active state of non-muscle myosin IIA and the capacity for acto-myosin contractility. Cells were stained with anti-phospho-MLC antibodies for monophosphorylated MLCIIa (Ser19) (p-MLCII) and biphosphorylated MLCIIa (Thr18/Ser19) (pp-MLCII). Automated high-content imaging using Gabor image structure analysis (scale: 2px, wavelength: 8, number of angles: 8 with regional intensity) allowed quantification of fibre-like structures associated with acto-myosin contractility.

### Small molecule library and other compounds

The Tocriscreen Total drug screen library of 1120 biologically active compounds with wide chemical diversity was obtained from Tocris (product no. 2884). Compounds were supplied at 10 mM in DMSO. Fasudil hydrochloride was obtained from Cell Guidance Systems (SM-49), belumosudil (KD025) from Cayman Chemical (17055), and hydroxyfasudil (S8208) and ripasudil (S7995) were obtained from Selleckchem. 8-Br-cAMP (B7880) was obtained from Sigma.

### Drug screening: primary and secondary screen

**Fig. 1:**
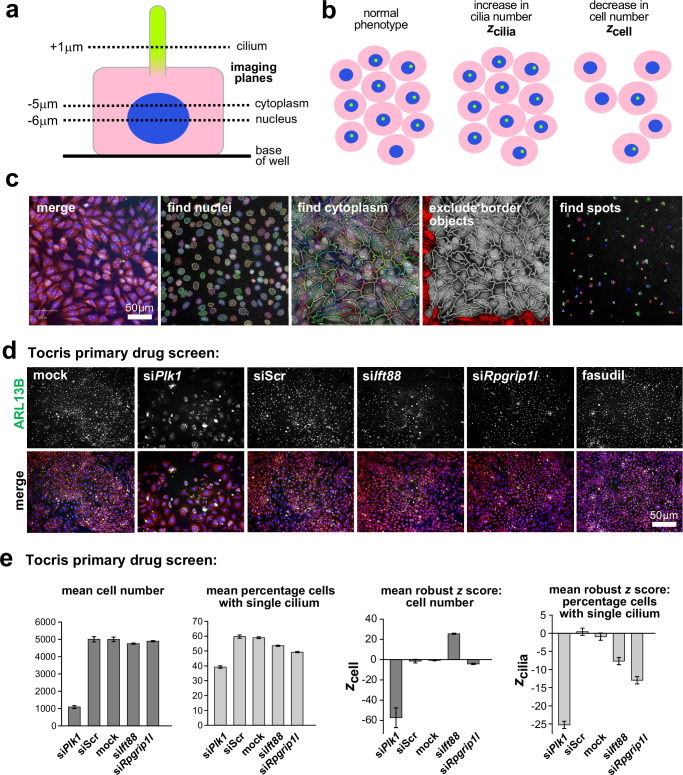
High-content imaging protocol for high-throughput ciliogenesis screening. **a** Schematic of a polarised mIMCD3 cell showing the focal planes used to image nuclei (blue), cytoplasm (pink) and ciliary axonemes (green). **b** Robust *z* scores were calculated to identify significant changes in cilia incidence (*z*_cilia_) or in cell number (*z*_cell_). **c** mIMCD3 cells imaged using an Operetta high-content imaging system. Merge images show staining for cilia marker ARL13B (green), nuclei (DAPI; blue) and cellular RNA (TOTO-3; pink). Representative images shown from Harmony/Columbus software of cell segregation and cilia recognition (“find spots”) protocols. Scale bar = 50 µm. **d** Representative immunofluorescence high-content images from the Tocris primary drug screen showing decrease of cilia incidence following reverse transfection with siRNA pool against *Plk1* (a positive control for transfection since efficient transfection results in cell death), *Ift88* and *Rpgrip1l* (positive controls targeting essential ciliary gene transcripts that abrogate ciliogenesis) compared to the non-targeting scrambled siRNA (siScr). Following knockdown with si*Rpgrip1l*, cilia incidence was rescued with 10 µM fasudil, which was one of the top hits from the primary screen (see Fig. 2). Scale bar = 50 µm. **e** Left: bar graphs quantitate the effect on cell number and ciliogenesis (mean % cells with a single cilium) for positive controls (*Plk1*, *Ift88* and *Rpgrip1l* siRNAs) and negative controls (siScr and mock transfections). Right: bar graphs showing mean robust *z* scores for cell number (*z*_cell_) and mean robust *z* score for cilia incidence (*z*_cilia_) for positive and negative siRNA controls. Error bars indicate s.e.m. for a total of *n* = 14 replicates (*n* = 2 passages, each comprising 2 technical replicates per plate and 2 knockdown conditions).

mIMCD3 cells were seeded in 96-well ViewPlates (PerkinElmer) at 8000 cells per well in Opti-MEM. Cells were reverse transfected with siRNA targetting *Rpgrip1l* transcripts (si*Rpgrip1l*, designed against RefSeq NM_173431) encoding the essential ciliary protein RPGRIP1L, or a non-targetting (scrambled) siRNA negative control (siScr), using Lipofectamine RNAiMAX as instructed by the manufacturer. Control transfections of si*Ift88*, si*Mks1* and a mock transfection were also included. Chemicals were added 24 h after cell plating at a final concentration of 10 µM. The final concentration of the DMSO vehicle in each well was 0.1%. Cells were fixed and stained 72 h after seeding and 48 h after the addition of chemicals. Cells were fixed using ice-cold methanol and permeabilised using 0.1% Triton X-100 in PBS. Cells were stained using anti-ARL13B (Proteintech) at 1:6000, followed by staining with goat anti-rabbit Alexa Fluor 488 (A11034, Fisher) at 1:2000, DAPI (Fisher) at 1:5000 and TOTO-3 (Fisher) at 1:5000. High-content imaging and analysis were as described above. Robust *z* scores^19^ were calculated to test for statistical significance in comparisons of cell number and cilia incidence (*z*_cell_ and z_cilia_). Mean values for two passages were used to identify chemicals that significantly restored cilia after knockdown without significant effects on cell number. The high reproducibility of these knockdown assays in the primary screen was confirmed by Pearson’s correlation coefficient (R^2^) values of 0.863 and 0.781 for si*Rpgrip1l* and siScr knockdowns, respectively, for z_cilia_ between the two replicates of the screen. The strictly standardised mean difference (SSMD) for the screen, which quantifies the difference between negative and positive siRNA controls^20^, had a mean value of 2.702, indicating that the siRNA effect size was strong (3 > SSMD ≥ 2) as defined previously^21^. A transfection control (si*Plk1*) resulted in a 78.4% mean reduction in cell number compared to negative controls (mean *z*_cell_ −57.36), indicating that siRNA-reverse transfection was highly efficient (Fig. 1d, e). Transfection of positive controls si*Ift88* and si*Rpgrip1l* resulted in significant decreases in cilia incidence (percentage of cells with a single cilium reduced by 9.8% and 17.1%, z_cilia_ −7.67 and −12.96, respectively) compared to negative controls (Fig. 1d, e).

### Drug screening: tertiary screen

hTERT RPE-1 cells were seeded at 20,000 cells per well in DMEM/F12 0.2% foetal calf serum (FCS) in 96-well ViewPlates coated with Matrigel Matrix (Corning). A similar methodology was used for the secondary screen except that cells were reverse transfected with si*IFT88*, as well as si*RPGRIP1L* and siScr.

### MTT assay

hTERT RPE-1 cells were seeded at 11,200 per well in DMEM/F12 0.2% FCS in a 96-well plate. mIMCD3 cells were seeded at 12,800 cells per well in Opti-MEM in a 96-well plate. Fasudil hydrochloride was diluted in DMSO and added to cells at a final concentration of 0.1, 0.3, 1, 3, 10, 30 and 100 µM. At each time-point, media was removed and 50 µl of MTT reagent at 1 mg/ml added. Plates were incubated for 3 h in darkness at 37 °C. Any remaining MTT solution was removed, and dark blue formazan precipitates were solubilised in 100 µl propan-1-ol. Optical density was measured at 570 nm using a Mithras Berthold LB940 plate reader.

### Generation of hTERT RPE-1 CRISPR/Cas9 gene-edited cell lines

hTERT RPE-1 cells were CRISPR edited using guide RNAs (gRNAs) designed using the online CRISPR Design tool (crispr.mit.edu [no longer available]) to target the coding exons of *RPGRIP1L* (GGTCCAACTGATGAGACTGC) and *IFT88* (GGGCCCCTTGACTGACTAAG). gRNA for *TMEM67* (TAACAAATGTTGGCTCACAT) was designed using Benchling (www.benchling.com/). *BbsI* restriction sites were added, and reverse HPLC-purified oligonucleotides were obtained commercially. Oligos were annealed and then cloned into the pX458 CRISPR/Cas9 expression vector. The cloned vectors were transfected into hTERT RPE-1 cells using Lipofectamine 2000. Transfected cells were single cell sorted by FACS into 96-well plates 24 h post-transfection. Genomic DNA was extracted from confluent colonies, the targeted regions amplified and colonies with heterozygous variants identified using a T7 endonuclease digestion. Variants were identified using Sanger sequencing. Heterozygous knockout lines included *IFT88*^+/^^−^, *RPGRIP1L*^+/^^−^ and *TMEM67*^+/^^−^, as well as a biallelic knockout line *TMEM67*^−^^/^^−^. The null mutations were: *IFT88* (NM_175605.5):[c.371_408delinsAAGAAAAAAG,p.(P124Qfs*15)], *RPGRIP1L* (NM_015272.5):[c.15_37del, p.(D6Cfs*35)], *TMEM67* (NM_153704.6): [c.369delC, p.(E124Kfs*12)] and *TMEM67* (NM_153704.6):[c.519delT, p.(C173Wfs*20)];[c.519dupT, p.(E174*)]. We confirmed reduced expression of IFT88, RPGRIP1L and TMEM67 in each cell line, respectively, by western blot or by immunofluorescence. Cell lines were assessed for cilia incidence and cilia length. Cilia incidence was determined using the same method as for the secondary siRNA screen. Briefly, cells grown without serum for 48 h were methanol fixed and immuno-stained for acetylated α-tubulin (1:4000) and stained with DAPI (1:2000) and TOTO-3 (1:4000). Plates were imaged at 20× using a high-throughput microscope (PerkinElmer Operetta® microscope controlled using Harmony® high-content analysis software). Image data was analysed using the Columbus™ Image Data Storage and Analysis system (PerkinElmer). Image data was analysed to identify whole nuclei, cell boundaries and cilia. For determination of cilia length, a proxy measure of pixel area of alpha tubulin staining was obtained for each cilium identified by the software.

### Dose response assays

hTERT RPE-1, hTERT RPE-1 *IFT88*^+/^^−^, hTERT RPE-1 *RPGRIP1L*^+/^^−^, hTERT RPE-1 *TMEM67*^+/^^−^ and hTERT RPE-1 *TMEM67*^−^^/^^−^ cell lines were seeded at 9000 cells per well in DMEM/F12 plus 0.2% FCS in 96-well ViewPlates coated with Matrigel matrix. mIMCD3 cells were seeded at 9000 cells per well in Opti-MEM in uncoated 96-well View-plates. Fasudil hydrochloride, hydroxyfasudil and ripasudil were diluted in DMSO. Chemicals were added, and cells were stained as previously described in the “Drug screening” section.

### Cell cycle analysis

100,000 hTERT RPE-1 cells were seeded in 6-well plates in DMEM/F12 medium either with or without 10% FCS. The next day, cells were treated with a final concentration of 5 µM hydroxyfasudil or vehicle control (0.1% DMSO). After 48 h of drug treatment, all cells were collected, washed with PBS and then fixed by adding ethanol dropwise to a concentration of 70%. Cells were resuspended by vortexing at low speed. Cells were fixed overnight at −20 °C, pelleted at 160 × *g* at 4 °C, washed in 500 μl cold FACS buffer (0.1% BSA, 0.1% Triton X-100 in PBS) and resuspended in 500 μl propidium iodide staining solution (20 μg/ml propidium iodide, 200 μg/ml RNase in FACS buffer) and passed through a 70 μm cell strainer. Stained cells were incubated overnight in the dark at 4 °C. The intensity of propidium iodide staining and cell size were captured for a minimum of 1000 events using the CytoFLEX S (Beckman Coulter) at low speed. Data were analysed using ModFit LT (v3.2, Verity Software House).

### Assessment of basal body docking at the apical cell surface

20,000 mIMCD3 cells were seeded in DMEM/F12 supplemented with 10% FCS in 12-well plates. The following day, either fasudil or hydroxyfasudil to a final concentration of 5 µM or DMSO to a final concentration of 0.1% (vehicle control) were added to the media. After 48 h, the serum was withdrawn, and after a further 16 h, cells were fixed with ice-cold methanol. Basal body docking was investigated by incubating cells with anti-ezrin (1:100) to stain the apical cell surface and anti-CEP164^17^ (1:10,000) to mark the mother centriole and distal appendages. *z* stacks were used to produce MIPs to identify ROIs for CEP164 for each stack. The intensity of CEP164 staining and ezrin staining was measured at each position in the stack, and the relative positions of each stain were determined. Basal body docking at the apical cell surface was defined as the most intensely stained slice for CEP164 being within two slices (1 µm) of the most intensely stained slice for ezrin. Ciliation as a proportion of docking was investigated by staining cells with anti γ tubulin (1:1000) and anti-ARL13B (1:2000) to mark the axoneme and the basal body and anti-CEP164 antibody as before. Cells were imaged from the ciliary tip to the loss of the CEP164 signal. Ciliation and the presence of basal bodies were assessed by manual grading of images for ARL13B/gamma tubulin staining and CEP164 staining.

### Assessment of vesicle trafficking

30,000 mIMCD3 cells were seeded in DMEM/F12 supplemented with 10% FCS onto coverslips in a 12-well plate. Cells were transfected the following day with Rab8a-GFP plasmid (#49543)^22^ using polyethylenimine transfection reagent in Opti-MEM. Four hours after transfection, either fasudil or hydroxyfasudil to a final concentration of 5 mM or DMSO to 0.1% (vehicle control) was added to the media. Cells were fixed with 10% neutral buffered formalin 24 h after transfection. After permeabilising cells with PBS + 0.1% Triton X-100, coverslips were blocked with 5% milk in PBS and incubated with anti-human ARL13B (1:1000) and anti-polyglutamylation modification (GT335, 1:1000) antibodies. After washing with PBS, cells were stained with secondary antibodies and DAPI. Coverslips were mounted using ProLongGold. Coverslips were imaged using 0.5 µm steps in nine-step *z* stacks on a Nikon A1R confocal microscope. Images were analysed using FIJI. Two fields of view (FOV) were analysed per biological replicate with three biological replicates. Cilia number was assessed using combined ARL13B and anti-polyglutamylation modification staining. Regions of interest were identified based on cilia positions on MIPs. Rab8a-GFP intensity was assessed at each step throughout the stack, the maximum value was taken, and an average intensity was calculated for Rab8a over the entire image.

### Luciferase assays

We used the murine NIH3T3 Shh-Light II reporter cell line, which contains the firefly luciferase reporter gene under the control of the Gli promoter. NIH3T3 Shh-Light II cells were reverse transfected in Opti-MEM and seeded at 14,400 per well in 96-well plates. Knockdowns included ciliary targets (si*Ift88* or si*Rpgrip1l*) and Patched1 (*Ptch1*), the Shh receptor. The latter was included as a positive control since knockdown stimulates the pathway by releasing Smoothened (Smo), the downstream G protein-coupled receptor, from the ciliary membrane^23^. Hydroxyfasudil, the positive control Smoothened agonist (SAG; Cayman 11914) or vehicle controls were added after 24 h to final concentrations of 10 µM and 250 nM, respectively. Cells were lysed using 30 µl passive lysis buffer (Promega). 10 µl of cell lysate was analysed using a Mithras Berthold LB940 plate reader in a white 96-well plate (Greiner) using injectors to dispense 20 µl luciferase assay reagent (Promega) followed by 20 µl Stop n Glo substrate (Promega) diluted 1:1 with filtered dH_2_O. Values were expressed as ratios of firefly luciferase: *Renilla* luciferase normalised to the values for the unstimulated DMSO vehicle control treated cells.

### SMO occupancy assay

mIMCD3 cells were seeded on coverslips at 1.1 × 10^5^ cells in 1 ml Opti-MEM. Chemicals were added after 24 h, and cells were fixed and stained after 72 h using 1:300 anti-SMO, 1:2000 anti-ARL13B (Neuromab) antibodies followed by goat anti-rabbit 488 and goat anti-mouse 568 both at 1:2000 and DAPI at 1:500. All antibodies were diluted in 3% goat serum. Slides were imaged using confocal microscopy as described above. Images were analysed using Columbus to identify cilia as regions of interest and the SMO occupancy within those regions. Values of total SMO area: cilia number ratios were used to control for variable cilia size.

### Analysis of primary whole genome siRNA screen data

Primary whole genome screen data was previously published in Wheway et al.^15^ and is freely available. The secondary screen hit list was generated by filtering out hits that had any significant changes to cell number in any experimental replicates (robust *z* score for cell number 2 ≥ x ≥ −2) and prioritising those that significantly increased cilia incidence in both experimental replicates (robust *z* score for cilia incidence x ≥ 2). Final hits were selected based on siRNA specificity and the existence of human orthologues.

### Secondary siRNA screen

Secondary screening was performed following the previously published whole genome screen protocol and validated controls^15^. 2.5 µl of ON-Target Plus siRNA libraries (Dharmacon) were reverse transfected into mIMCD3 cells (8000 cells/well) as 2 µM siRNA (final concentration 50 nM) using Lipofectamine RNAiMAX in Opti-MEM. Each 96-well plate included 8 different controls duplicated in columns 1 and 12. si*Plk1* as a control for transfection; si*Mks1*, si*Ift88* and si*Rpgrip1l* are positive controls for cilia loss; negative controls were siScr, si*MLNR* (designed against a human gene with no target in the mouse genome), and mock transfection with transfection reagent only. siRNA transfections were incubated for 72 h. Plates were fixed in ice-cold methanol and immuno-stained for acetylated α-tubulin (1:4000) and stained with DAPI (1:2000) and TOTO-3 (1:4000). Plates were imaged at 20× using a high-throughput microscope (PerkinElmer Operetta® microscope controlled using Harmony® high-content analysis software). Image data was analysed using the Columbus™ Image Data Storage and Analysis system (PerkinElmer). Image data was analysed to identify whole nuclei, cell boundaries and cilia. Robust *z* scores were used to determine significant hits within each experimental replicate. Negative controls were pooled (siScr, si*MNLR*, mock transfection) within each experimental replicate and compared to individual siRNA knockdown values for each quantified phenotype. Average robust *z* scores were calculated, and hits were classed as having an average robust *z* score for cilia incidence (*z*_cilia_) ≥2. Comparison of the two replicates of the screen showed that the mean R^2^ values between runs were 0.6366 for cell number and 0.7156 for cilia incidence, indicating high reproducibility of the assay.

### Tertiary siRNA screen

Tertiary screening was carried out in the human cell line hTERT RPE-1, using ON-Target Plus siRNAs against previously validated siRNA including human *RPGRIP1L* (RefSeq NM_015272) and an additional independent ciliary target, human *IFT88* RefSeq NM_175605^15^. We also used new, freshly prepared commercial batches of each chemical.

### Western blotting

Whole cell lysates were separated by SDS-PAGE and blotted onto PVDF membranes using the NuPAGE system and reagents (Invitrogen™). Membranes were blocked in 5% [w/v] milk/TBST and then immunoblotted for ROCK2 (1:1000), ROCK1 (1:1000), IFT88 (1:1000), RPGRIP1L (1:500–1000). GAPDH (1:5000), vinculin (1:5000) or β-actin (1:10,000) antibodies were used as loading controls.

### Plasmids

GFP-mROCK2 was a gift from Alpha Yap (Addgene plasmid #101296). GFP-mouse MLCII and GFP-MLCII^AA^ (constitutively inactive “TASA” Thr18>Ala18 Ser19>Ala19 double point mutant) constructs were the kind gift of Michael F. Olson, Toronto Metropolitan University, Toronto, Canada. 500 ng of plasmid DNA was transfected into 1×10^5^ hTERT RPE-1 cells in DMEM/F12 medium containing 10% FCS, on 13 mm coverslips using Lipofectamine 2000 (1:3 ratio DNA:Lipofectamine). Transfection complexes were removed after 4 h and replaced with fresh medium. At 24 h post-transfection, cells were serum-starved in DMEM/F12 0.2% FCS for 24 h before coverslips were fixed and prepared for immunofluorescence imaging.

### Inhibitor treatments

1 × 10^5^ cells were grown on 13 mm round coverslips in DMEM/F12 containing 10% FCS for 24 h and then serum-starved as above. Cells were then treated with either vehicle control (DMSO) or chemical inhibitors at varying concentrations, diluted to final working concentrations in DMEM/F12 0.2% FCS. Coverslips were fixed and prepared for immunofluorescence imaging.

### Generation of human iPSC CRISPR/Cas9 cell lines

AD2 human induced pluripotent stem cells were gene edited by transfection of a modified eSpCas9 plasmid containing the *RPGRIP1L* targeting guide sequence. Guides targeting exon 2 of *RPGRIP1L* (NM_015272.5) were designed using Benchling as previously described in the “Generation of hTERT RPE-1 CRISPR/Cas9 gene-edited cell lines” section. *BbsI* restriction sites were added to the guide selected, and the following oligos were annealed and cloned into the eSpCas9 plasmid (#79145, Addgene) following digestion with *BbsI*: 5′ CACCGGTCCAACTGATGAGACTGC 3′ and 5′ AAACGCAGTCTCATCAGTTGGACC. Guide efficiency was checked by transfecting the modified eSpCas9 plasmid into HEK293 cells and assessing gene editing via sequencing of *RPGRIP1L* exon 2 using primers *RPGRIP1L*_ex2F AAGCAGCACATGTGGACAAT and *RPGRIP1L*_ex2R CCAGGCAATTCAGTTTGGAG.

AD2 cells were nucleofected as previously described^16,24^ using the Human Stem Cell Nucleofector II Kit (Lonza Bioscience) according to the manufacturer’s instructions. Briefly, 8 × 10^5^ cells were resuspended in Nucleofector solution mix along with 2 µg of the modified eSpCas9 plasmid containing the RPGRIP1L targeting guide and nucleofected using the AMAXA nucleofector (Lonza Bioscience). Following nucleofection, cells were plated in mTESR plus (Stem Cell Technologies) containing ROCK inhibitor (Y27632, 1254 Tocris). ROCK inhibitor was withdrawn after 24 h. Fluorescence-assisted single cell sorting was carried out 48 h post-transfection using the Influx 6 way cell sorter (BD Biosciences). Single GFP positive cells were sorted into 96-well plates, and any resulting colonies were checked for editing as previously described in the “Generation of hTERT RPE-1 CRISPR/Cas9 gene-edited cell lines” section. Known off-target sites with scores of ≥0.5 that were in known protein coding sequences were also sequenced to ensure there was no known unintended off-target gene editing. CRISPR-Cas9 gene-edited cell lines were also analysed for karyotypic abnormalities using the hPSC Genetic Analysis Kit (Stem Cell Technologies) and stained for pluripotent markers OCT3/4 (1:15) and SSEA4 (1:50).

The effects of the mutations were assessed by western blotting. Protein was extracted using a radio-immunoprecipitation assay buffer supplemented with protease inhibitors. Protein was quantified using Bradford assay, and 30 µg of protein was separated by electrophoresis and blotted onto PVDF membrane as previously described in the “Western blotting” section. We characterised two mutant iPSC lines (Supplemental Fig. 7). The first (line 1) had compound heterozygous *RPGRIP1L* variants (NM_015272.5):[c.24_66del, p.(Ala9Glufs*14)];[c.24_66del, p.(Ala9_Phe22del)] for which levels of full-length RPGRIP1L protein were decreased by 61 ± 6.2% reduction compared to the isogenic control (Supplemental Fig. 7a, b). The second mutant iPSC line 2 had a homozygous insertion (NM_015272.5):[c.66dupT, p.(G23Wfs*26)];[c.66dupT, p.(G23Wfs*26)] with a 65.7 ± 3.3% decrease in RPGRIP1L protein levels (Supplemental Fig. 7a, b). Raw sequencing traces are provided in Supplemental Dataset 6.

### Stem cell differentiation and generation of kidney organoids

Control and CRISPR-Cas9 gene-edited cell lines underwent differentiation using a protocol modified from published protocols^25,26^. Briefly, 150,000 cells were seeded in 6-well Matrigel coated plates and grown in APEL2 media (Stem Cell Technologies) supplemented with 5% PHFMII (Gibco) with added 8 μM CHIR99021 (4423, Tocris) for 4 days followed by APEL2 supplemented with 200 ng/ml FGF9 (273-F9, R&D Systems) and 1 μg/ml heparin (#07980, Stem Cell Technologies) for a further 4 days. The media were changed every other day. Cells were then trypsinized, and 10,000 cells per well were seeded in a 96-well round-bottomed Lipidure (AMS.52000024GB1G, AMS Biotechnology) coated plate. The plate was centrifuged to allow the cells to form pellets. Following treatment with 5 μM CHIR90221 for 1 h, cells were incubated with 200 ng/ml FGF9 and 1 μg/ml heparin for a further 8 days. 72 h after the withdrawal of growth factors, cells were treated with the following drugs for 4 days: 5 μM hydroxyfasudil, 2.5 μM fasudil, 300 nM belumosudil, 1 μM belumosudil and 300 μM 8-Br-cAMP. Organoids were then fixed with PFA, and those to be sectioned were subsequently treated with 30% sucrose, washed and embedded in optimal cutting temperature compound. After 19 days, kidney organoids from both normal isogenic control and *RPGRIP1L* mutant iPSC line 1 expressed markers of mature kidney proximal tubule, distal tubule and nephron structures, indicating successful differentiation. *RPGRIP1L* mutant iPSC line 2 failed to differentiate into kidney precursor cells and was abandoned at day 8 of differentiation.

### Cyst counting

Prior to fixation, brightfield images at ×4 magnification were taken prior to and after drug treatments. These 2D images were visually assessed for signs of surface anomalies/cyst formation at the edge of each organoid. Anomalies were staged from 0 normal, 1 small bulges/points, 2 rounded cyst-like structures with smooth edges to 3 uneven cyst-like structures with broken borders. Anomalies classed as stage 2 or 3 were counted as cysts.

### Whole organoid and section staining

10 µm sections were obtained using a cryostat (CM1800, Leica) cooled to −18 °C and Celledge R+ blades (JDD-0200-00A, Cell Path). Sections were kept at −80 °C prior to staining. Sections underwent post-fixing with 10% neutral buffered formalin as previously described. Organoids and sections were blocked and permeabilised using PBS containing 5% donkey serum and 0.3% Triton X-100. Whole organoids (day 19 and day 23) were stained with antibodies to CDH1 (1:250) and NPHS1 (1:500) as well as LTL-FITC (1:250). Sections (day 23) were stained with ezrin (1:100) and ZO-1 (1:500). All were also stained with DAPI and LTL. Ezrin and ZO-1 staining was manually graded by two individuals blinded to the experimental conditions, mean gradings were used as the final result.

### Assessment of tubule area

Sections were stained with DAPI and with antibodies to ezrin (1:100) and ZO-1 (1:500). Images were taken and tubules identified via their distinctive morphology. The longest axis (a) and the corresponding perpendicular axis (b) of each tubule was quantified using Fiji, and the tubule area was calculated based on the shape of an ellipse (πab; Fig. 6b).

### Matrigel 3D cyst assays

3D Matrigel cyst assays were performed as previously described^27^. In brief, OX161/c1 cells (1 × 10^4^/well) were mixed with 70 μl Matrigel (Becton Dickinson, UK), plated into 96-well plates in triplicate and incubated for 30 min at 37 °C to facilitate gel formation. Cells were then cultured for 12 days in the presence of belumosudil. The media was replaced every 2 days. The average cyst area was calculated by measuring cyst areas in individual wells on day 12. At least 65 cysts were measured in triplicate wells at each time-point.

### Effect of belumosudil in vivo

A tetracycline-inducible kidney-specific Pkd1 mouse model (*Pax8*^*rtTA*^*-TetO-Cre-Pkd1*^*fl/fl*^)^28^ on a C57/BL6 background was obtained from the Baltimore PKD Centre. All procedures were carried out at the University of Sheffield Medical School. Mixed sex cohorts were studied from PN13-22. The animals had not undergone any prior experimental procedures and were healthy. At PN12, animals were genotyped and distributed into appropriate groups.

At PN13-15, intraperitoneal injection of doxycycline (50 mg/kg/day) was performed in all experimental groups on 3 consecutive days to induce deletion in of *Pkd1* in the kidney at an early time-point to generate a polycystic kidney disease mouse model.

Following doxycycline injections, groups of mice were treated with 5, 10 or 25 mg/kg/day of belumosudil or vehicle (5% DMSO, 35% polyethylene glycol 300, 2% Tween 80 in sterile distilled water) by intraperitoneal (IP) injection for 7 days. The injection site was alternated. The effectiveness of belumosudil in reducing kidney weight/body weight and cystic index was evaluated after 7 days of treatment by IP injections.

Mice were monitored daily until the end of the experiment. Monitoring consisted of weighing the animals and physical inspection daily for any sign of discomfort or illness.

After sacrifice at PN23, following a schedule 1 method, kidneys were collected, and each was cut transversally into four pieces. The top and bottom sections were snap frozen for biochemical analysis, whereas the middle section was embedded in cry-M-bed solution (Wolflabs) or immersed in 10% neutral buffered formalin (MilliporeSigma) for histological analysis. Tissue sections were analysed for cystic index or processed for immunofluorescence staining. Microscope images were subsequently used to calculate the tubule cytosol area.

Animals were only excluded if they had to be culled due to adverse events, but no animals reached this stage, so all were included, and all data were reported. Treatments, treatment order and randomisation were carried out by an animal house technician with no input from those directing the study. The effects of cage position were not taken into account. During the experimental procedure, only the animal house technician was aware of the group allocation. Once animals were euthanised, treatments were unblinded to the individual processing the samples and taking microscope images. Blinded images were presented to the individuals measuring tubules, which were unblinded upon statistical analysis being obtained.

### Statistics, reproducibility and screen quality controls

Robust *z* scores^19^ for cell number (*z*_cell_) and the percentage of cells with a single cilium (*z*_cilia_) were calculated for all results, compared to the median and median absolute deviation (MAD) of all positive and negative controls in a processed batch. This allowed the application of meaningful statistical cut-off values (*z*_cilia cutoff_ and *z*_cell cutoff_) for the identification of significant “hits” affecting ciliogenesis based on the median and MAD of positive and negative controls per four-plate batch^20^. The strictly standardised mean difference (SSMD) of the percentage of cells with a single cilium in *Ift88* and *Rpgrip1l* knockdowns, compared to all negative controls, was consistently >1.5, reflecting the suitability of these siRNAs as positive controls for effects on ciliogenesis and the consistency and quality of the screen^20,21^. For cell number, cilia incidence and cilia length measurements normal distribution of data was confirmed using the Kolmogorov–Smirnov test (GraphPad Prism software). Pair-wise comparisons were analysed with Student’s two-tailed t-test (unless otherwise stated) or one-way analysis of variance (ANOVA) using InStat (GraphPad Software). Cell cycle profile data were analysed using Chi-squared tests. Significance of pairwise comparisons indicated as: ns not significant, *p < 0.05, **p < 0.01, ***p < 0.001, ****p < 0.0001. Results reported are from at least three independent experimental replicates, apart from drug and reverse genetics screens, which comprised of two replicates (runs). Error bars on graphs represent s.e.m. unless otherwise stated.

### Reporting summary

Further information on research design is available in the Nature Portfolio Reporting Summary linked to this article.

## Results

### Drug screens

We selected the Tocriscreen Total library of 1120 biologically active clinical development compounds to screen for potential drugs that restore cilia after small interfering RNA (siRNA) knockdown. We carried out the screen using the mouse inner medullary collecting duct (mIMCD3) cell line, which displays primary cilia that are easy to detect, using automated imaging platforms (Fig. 1a–c). Robust *z* scores^19^ were calculated to test for statistical significance in comparisons of cell number and cilia incidence (*z*_cell_ and z_cilia_). A control for transfection efficiency (si*Plk1*) resulted in a significant reduction in cell number compared to negative controls, whereas transfection of the positive control siRNAs, targeting transcripts from the essential ciliary genes si*Ift88* and si*Rpgrip1l*, resulted in significant decreases in cilia incidence (Fig. 1d, e; see the “Methods” section for further details).

**Fig. 2:**
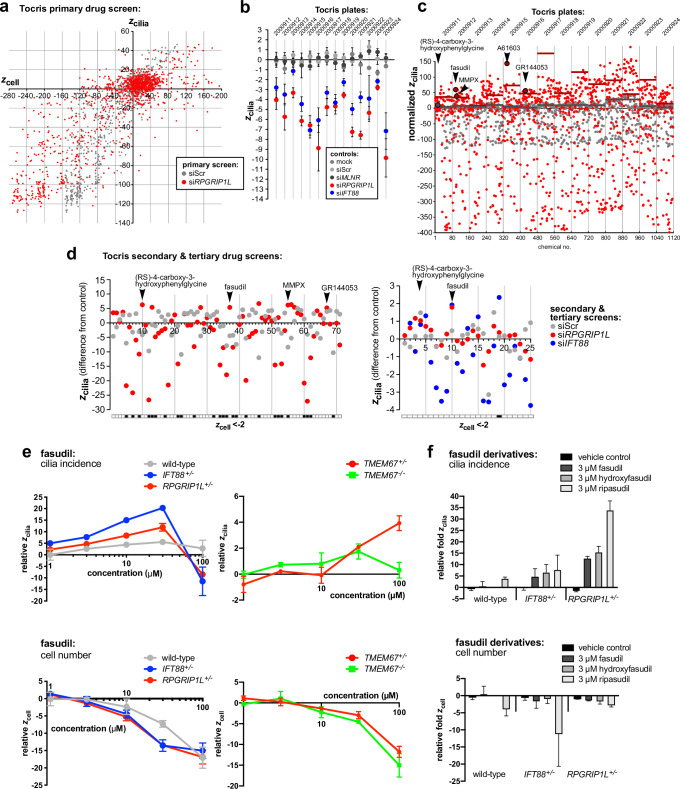
High-throughput screens of cilia incidence using the Tocriscreen Total library of active clinical development compounds and dose response for candidate hit fasudil and derivatives. **a** Primary drug screen summarising mean *z*_cell_ and *z*_cilia_ values (*n* = 2 experimental replicates) for mouse mIMCD3 cells treated with each chemical and reverse transfected with either si*Rpgrip1l* (red) or siScr (grey). **b** Summary of negative (grey) and positive (red & blue) control *z*_cilia_ values for each Tocris plate. Error bars indicate s.e.m. for *n* = 2 experimental replicates, each with 4 technical replicates. All plates passed the quality control criteria of *z*_cilia_ ≤ −2 for positive controls (si*Ift88* and si*Rpgrip1l*) and −2 ≤ *z*_cilia_ ≤ +2 for negative controls (mock, siScr, si*MLNR*). **c** Normalised *z*_cilia_ values for cells treated with each chemical and reverse transfected with either si*Rpgrip1l* (red) or siScr (grey). Tocris plate numbers are indicated along the x-axis. Normalised *z*_cilia_ cut-off values (coloured bars) for each plate are indicated (coloured bars). Hits are defined as chemicals with normalised *z*_cilia_ greater than cut-off values. Selected hits are indicated (*n* = 2 experimental replicates). **d** Left: secondary screen (*n* = 2 biological replicates in cells treated with siScr, one biological replicate in cells treated with si*Rpgrip1l*) of 71 chemicals in mIMCD3 cells. Hits are defined as chemicals with *z*_cilia_ difference from relevant control of ≥+2. Hits were excluded if *z*_cell_ ≤ −2 (black filled squares in the grid). Right: tertiary screen (*n* = 2 biological replicates, with three experimental replicates for each knockdown condition) of 25 chemicals in human hTERT RPE-1 cells. Hits are defined as chemicals with *z*_cilia_ difference from relevant control of ≥1.5. Hits were excluded if *z*_cell_ ≤ −2 (black filled squares in the grid). **e** Dose response assays in ciliopathy crispant hTERT RPE-1 cells (wild-type parental line, heterozygous *IFT88*^+/^^−^, *RPGRIP1L*^+/^^−^ and *TMEM67*^+/^^−^, and biallelic *TMEM67*^−^^/^^−^ knockout line) for concentration range of 1–100 µM fasudil. Graphs show mean robust *z* scores (*n* = 2 experimental replicates, each with *n* = 3 technical replicates) for cilia incidence (*z*_cilia_; upper panels) and cell number (*z*_cell_); lower panels. Values are normalised to vehicle control (DMSO) treated cells. Error bars represent the range. **f** Dose response assays in cells of the indicated genotype for 3 µM fasudil and derivatives hydroxyfasudil and ripasudil. Bar graphs show the fold-change in *z*_cilia_ (top) and *z*_cell_ (bottom) relative to vehicle control (*n* = 2 experimental replicates, each with *n* = 3 technical replicates). Error bars represent the range.

For each screening batch (corresponding to four 96-well plates within the two replicates), we normalised robust *z* scores across the screen to set cut-off values so that we could select hits within each batch (Fig. 2a–c). Hits were selected if z_cilia_ ≥ z_ciliacutoff_ (where z_ciliacutoff_ = normalised z_cilia_ of batch controls + 2). Chemicals were excluded from further analysis if z_cell_ ≤ z_cellcutoff_ (where z_cellcutoff_ = normalised z_cell_ of batch controls − 2) for >2 of the 4 plates in each batch to remove those that had a significant cytotoxic effect. Following the normalisation of cut-off values, we selected 73 chemicals for secondary screening, although only 71 were commercially available (Supplemental Data 1). Secondary screening in mIMCD3 cells identified 25 hit compounds (Fig. 2d). We then did a tertiary screen in a cell line of a different species (the ciliated human cell line hTERT RPE-1), using siRNAs of a different chemistry against human *RPGRIP1L* (RefSeq NM_015272) and an additional independent ciliary target, human *IFT88* (RefSeq NM_175605), encoding the essential ciliary intraflagellar transport protein (IFT88). We also used new, freshly prepared commercial batches of each chemical. The tertiary screen validated two chemicals, fasudil hydrochloride and (*RS*)-4-carboxy-3-hydroxyphenylglycine (Fig. 2d). Fasudil hydrochloride (hereafter referred to as fasudil) increased cilia incidence despite knockdowns of IFT88 and RPGRIP1L (z_cilia_ increases of +1.92 and +1.79, respectively, compared to negative controls) without any cytotoxic effect (no significant decrease in z_cell_ values). Negative control cells transfected with siScr (scrambled siRNA) and treated with fasudil also had no significant differences in either z_cell_ or z_cilia_, suggesting that fasudil was not cytotoxic and did not affect cilia incidence in normal ciliated cells (Fig. 2d). (*RS*)-4-carboxy-3-hydroxyphenylglycine increased cilia incidence (z_cilia_ increases of +0.82 and +1.04 for si*IFT88* and si*RPGRIP1L*, and +1.48 for siScr) without any significant effects on z_cell_ (Supplemental Fig. 1a–c) and was also taken forward to dose response testing.

### Dose response testing of fasudil

Fasudil (also known as HA-1077 and AT877) is an isoquinolone derivative based on the core structure of 5-(1,4-diazepan-1-ylsulfonyl)isoquinoline and is a potent but broadly selective Rho-kinase (ROCK) inhibitor of both ROCK1 and ROCK2 isozymes. Fasudil is currently unlicensed by the Federal Drugs Agency (FDA) and the European Medicines Agency (EMA) but is in clinical use in Japan and China and in trials elsewhere as a treatment for cerebral vasospasm^29^, pulmonary hypertension^30^, to reduce cognitive decline in stroke patients^31^ and for amyotrophic lateral sclerosis^32^.

We tested the effect of fasudil on cell viability, performing MTT assays in both mIMCD3 and hTERT RPE-1 cell lines (Supplemental Fig. 1a). For mIMCD3 cells, the mean metabolic activity ranged between 81.6% and 96.9% compared to vehicle control across all time points for the range of concentrations tested. hTERT RPE-1 cells were more sensitive to the cytotoxic effects of fasudil, with mean metabolic activity that ranged from 66.9% at 100 µM to a maximum of 93.3%. Next, following siRNA knockdowns, we tested dose responses in both mIMCD3 and hTERT RPE-1 cell lines for fasudil concentrations ranging from 1 µM to 100 µM (Supplemental Fig. 1b), confirming significant rescue of cilia incidence following either siI*ft88* or si*Rpgrip1l* knockdowns even with 1 µM fasudil. Significant rescue was maintained up to 30 µM, but with significant cytotoxic effects (z_cell_ < −2) at concentrations ≥30 µM. Dose response assays in hTERT RPE-1 cells showed significant rescue at 10 µM and higher fasudil concentrations following si*RPGRIP1L* knockdown (Supplemental Fig. 1c).

(*RS*)-4-carboxy-3-hydroxyphenylglycine is a broad-spectrum glutamatergic antagonist and is currently unlicensed for clinical use. Since the effect on cilia incidence was less significant than for fasudil in both tertiary and dose response testing, (*RS*)-4-carboxy-3-hydroxyphenylglycine was not investigated further.

### Fasudil and derivatives restore primary cilia in CRISPR-Cas9 edited cell lines

We then investigated the effects of fasudil in alternative cellular models of ciliopathies. We used CRISPR-Cas9 editing to create heterozygous knockout lines *IFT88*^+/^^−^, *RPGRIP1L*^+/^^−^ and *TMEM67*^+/^^−^, as well as a biallelic knockout line *TMEM67*^−^^/^^−^, confirming loss or reduced expression of the encoded protein (Supplemental Fig. 2a–c). The *IFT88*^+/^^−^ and *RPGRIP1L*^+/^^−^ lines had decreased cilia incidence and significantly reduced cilia length (Supplemental Fig. 2d). In dose response assays, cilia incidence was rescued in the *IFT88*^+/^^−^ and *RPGRIP1L*^+/^^−^ cell lines at 1 µM fasudil, the lowest concentration tested, but there were significant cytotoxic effects ≥10 µM (Fig. 2e). For the *TMEM67*^+/^^−^ cell line, fasudil did not rescue cilia incidence without significant cytotoxic effect, and for the *TMEM67*^−^^/^^−^ cell line, cilia incidence was not rescued at any tested concentration of fasudil. This suggests that fasudil is only effective in ameliorating phenotypes in certain ciliopathy disease types.

Fasudil is an inhibitor of both ROCK isozymes, ROCK1 and ROCK2, with half-maximal inhibition concentration (IC_50_) ranges of 0.84–2.67 and 0.69–1.90 µM, respectively^33–35^ (Supplemental Table 1). However, fasudil has off-target effects on other kinases (e.g. >75% inhibition of MAPK15, RPS6KA1, PKN2 and RPS6KA at 10 µM^36^), which may contribute to the cytotoxicity observed at high concentrations in our dose response testing. We therefore investigated two other derivative compounds based on 5-(1,4-diazepan-1-ylsulfonyl)isoquinoline; hydroxyfasudil and ripasudil. Hydroxyfasudil (also known as HA-1100), the major active metabolite of fasudil, has IC_50_ values of 0.73 µM and 0.72 µM for (human) ROCK1 and ROCK2, respectively^33^ and has a more favourable kinase inhibition profile^36^. Ripasudil (also known as K115) is currently in clinical use as a topical treatment for glaucoma and ocular hypertension^37,38^ and is a more selective inhibitor of ROCK2 with IC_50_ of 0.051 µM for ROCK1 and 0.019 µM for ROCK2^39^. Both hydroxyfasudil and ripasudil significantly increased cilia incidence relative to fasudil, in both the *IFT88*^+/^^−^ and *RPGRIP1L*^+/^^−^ gene-edited cell lines (Fig. 2f), but ripasudil was significantly more cytotoxic at 3 µM and was not considered for further analysis.

ROCK inhibition mediates functional increases of ciliary vesicle trafficking and Shh signalling.

Since actin remodelling pathways are activated by ROCK and are known to be important in ciliogenesis and cilia maintenance^40,41^, we hypothesised that ROCK inhibition by fasudil or hydroxyfasudil would result in visible changes to the actin cytoskeleton. We assessed levels of active phosphorylated non-muscle myosin light chain II (MLCII), which indicates capacity for acto-myosin contractility, by using automated high-content imaging of mIMCD cells. Levels of biphosphorylated MLCII (Thr18/Ser19) (pp-MLCII) were lower, but not significantly so (*p* = 0.055), in fasudil-treated cells, compared with cells treated with vehicle control, whereas levels of MLCII and phosphorylated MLCII were similar under both conditions (Supplemental Fig. 3a).

**Fig. 3:**
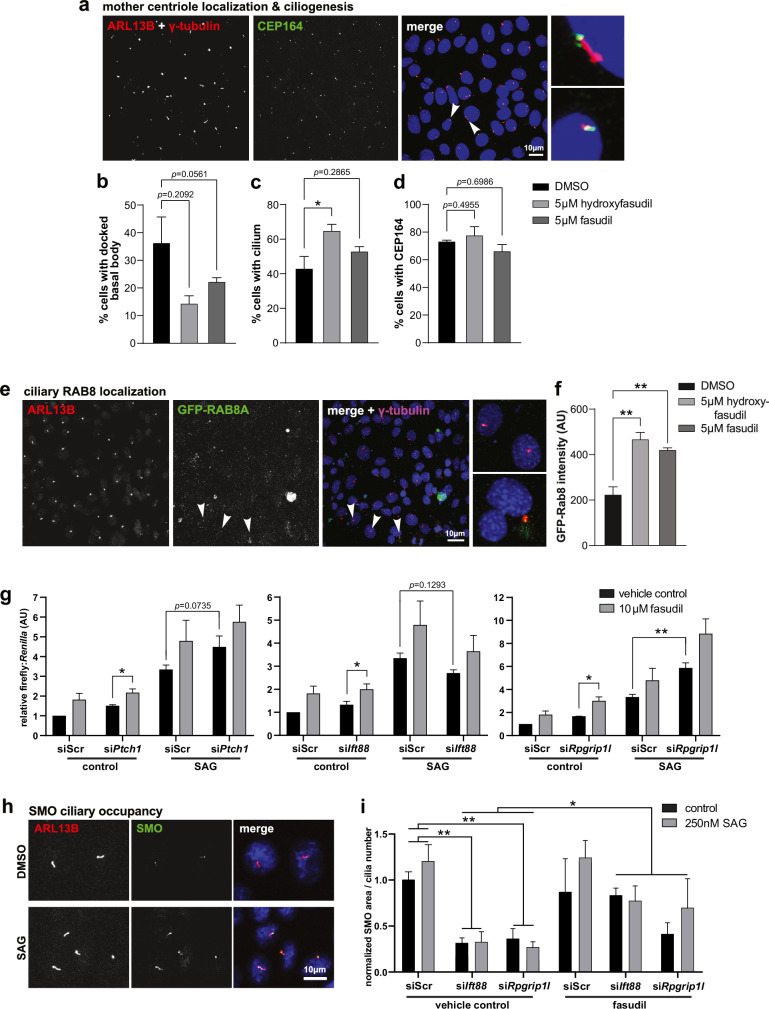
Inhibition of ROCK increases ciliary vesicle trafficking and restores ciliary-mediated Shh signalling. **a** Representative mIMCD3 cells stained for primary cilia markers ARL13B and γ tubulin (left; both red), and CEP164 (middle; green) to show the subdistal appendages and mother centriole. Smaller panels (right) show magnified examples of cilia. Scale bar = 10 µm. **b** Bar graph quantifies the percentage of mIMCD3 cells with a basal body docked at the apical membrane but with no ciliary axoneme, as measured by staining of CEP164 but not ARL13B. One-way ANOVA with Dunnett’s multiple comparisons test: DMSO vs 5 µM hydroxyfasudil: ns *p* = 0.0561, DMSO vs 5 µM fasudil: ns *p* = 0.2092, both F = 3.947 df = 6. **c** Percentage of cells with a cilium stained for both ARL13B and γ tubulin. DMSO vs 5 µM hydroxyfasudil: *p = 0.0260, DMSO vs 5 µM fasudil: ns *p* = 0.2865, both F = 5.751, df = 6. **d** Percentage of cells positive for CEP164 foci. DMSO vs 5 µM hydroxyfasudil: ns *p* = 0.6986, F = 1.660, df = 6. DMSO vs 5 µM fasudil: ns *p* = 0.4955, F = 5.751, df = 2. **e** Representative mIMCD3 cells stained for ARL13B (red), γ tubulin (magenta) and expressing GFP-RAB8A (green). Smaller panels (right) show marker colocalisation, indicative of cilia vesicle trafficking during ciliogenesis in magnified images of cells indicated by arrowheads. Scale bar = 10 µm. **f** Mean intensity of ciliary GFP-RAB8A (>98 cilia per FOV). DMSO vs 5 µM hydroxyfasudil **p = 0.0029, DMSO vs 5 µM fasudil **p = 0.0083, F = 16.59, df = 6. For (**c**–**f**), all assays performed with *n* = 3 biological replicates, 2 technical replicates, and error bars indicate s.e.m. **g** Bar graphs of dual (firefly & *Renilla*) luciferase Gli reporter assays showing the effects of si*Ptch1*, s*iIft88* and si*Rpgrip1l* knockdown compared to siScr in mouse Shh-Light II NIH3T3 cells. Cells were treated with 250 nM Smoothened agonist (SAG) and/or 10 µM hydroxyfasudil and compared with vehicle (DMSO) controls, with data normalised to controls transfected with siScr. SAG significantly increased the relative firefly:*Renilla* luciferase ratios following all knockdown treatments (not marked on the graph for reasons of clarity): si*Ptch1* (*p = 0.01132, t = 4.078) s*iIft88* (*p = 0.01830, t = 3.968) and si*Rpgrip1l* knockdowns (**p = 0.007303, t = 4.156, all unpaired two-tailed Student’s t-test, *n* = 3 biological replicates, 3 technical replicates, 4 treatments, df = 4). In the presence of SAG, si*Ift88* decreased whereas si*Ptch1* increased Shh signalling (n.s. *p* = 0.07351, t = 3.442 and ns *p* = 0.12934, t = 3.085, respectively; *n* = 3 biological replicates, with 3 technical replicates, df = 4). Knockdown with si*Rpgrip1l* in the presence of SAG significantly increased Shh signalling (**p = 0.007224, t = 4.158; *n* = 3, total of *n* = 6 technical replicates, df = 4), consistent with previous studies in the *Rpgrip1l*^−^^/^^−^ knockout mouse^82^. ROCK inhibition significantly increased the luciferase ratio in two knockdown conditions when compared to the vehicle control, even without SAG stimulation (si*Ptch1* *p = 0.02834, t = 3.8472, si*Ift88* ns *p* = 0.07092, t = 3.4608, si*Rpgrip1l* *p = 0.01843, t = 3.8425; *n* = 3, with 3 technical replicates, df = 4). Error bars represent s.e.m. **h** Representative mIMCD3 cells treated with SAG or vehicle control, stained for ARL13B (red) and Smoothened (SMO; green). SAG treatment increased SMO levels within cilia. Scale bar = 10 µm. **i** Normalised area of SMO localisation per cilium for mIMCD3 cells knocked down with siScr, si*Ift88* or si*Rpgrip1l*. Knockdown with si*Ift88* or si*Rpgrip1l* significantly reduced SMO localised to each cilium when treated with vehicle control (**p = 0.001333, t = 4.8533 and **p = 0.002788, t = 4.7277, respectively, unpaired two-tailed Student’s t-tests; *n* = 2 biological replicates, *n* = 2 technical replicates, 2 conditions, df = 2). Treatment with 10 µM fasudil increased ciliary SMO (*p = 0.01940, t = 4.4659; *n* = 2 biological replicates, 2 technical replicates, 4 conditions, df = 2). A minimum of 19 cilia were analysed per replicate. Error bars represent s.e.m.

To determine the mechanism of ROCK inhibition on ciliogenesis, we prioritised hydroxyfasudil because this had a greater effect on cilia incidence without cytotoxicity (Fig. 2f). We confirmed that treatment of hTERT RPE-1 cells with 5 µM hydroxyfasudil had no significant effects on cell cycle profiles for cells grown in conditions with serum (*p* = 0.692), or for cells grown in conditions without serum (*p* = 0.992) (Supplemental Fig. 4). We next assessed subdistal appendage localisation at the apical cell surface membrane using CEP164^42^ and ezrin, respectively, to determine the amount of basal body docking (Fig. 3a). Cells treated with 5 µM hydroxyfasudil had a marginal decrease of apically localised basal bodies with CEP164 staining (*p* = 0.0561; Fig. 3b) and, as expected, a significant increase in ciliary incidence (*p* = 0.0260; Fig. 3c), but there were no significant differences in the overall incidence of cells expressing CEP164 following treatment with either hydroxyfasudil or fasudil (Fig. 3d). These findings indicate that ROCK inhibition has minimal effects on basal body docking prior to ciliogenesis. We then investigated the effect of ROCK inhibitors on ciliary vesicle trafficking using RAB8A as a marker (Fig. 3e). RAB8 localises to ciliary vesicles and membranes^22,43^, including the ciliary base and growing axoneme, but is lost from mature cilia^44^. We transfected GFP-tagged RAB8A^22^ into mIMCD3 cells and observed significantly increased ciliary RAB8A following treatment with both hydroxyfasudil and fasudil (*p* = 0.0029 and 0.0083, respectively; Fig. 3f). This suggests that ROCK inhibition increases ciliary vesicle trafficking and is a significant mechanism by which cilia incidence is increased.

To test if primary cilia restored by ROCK inhibition are functional, we assessed cellular responsiveness to Sonic Hedgehog (Shh) signalling using the murine NIH3T3 Shh-Light II reporter cell line. We reverse transfected the Light II cells with si*Ift88* or si*Rpgrip1l* against ciliary targets or si*Ptch1* as a positive control. Treatment with Smoothened agonist (SAG) exacerbated Shh responses in all knockdown conditions (si*Ptch1 p* = 0.01132, si*Ift88 p* = 0.01830, si*Rpgrip1l p* = 0.007303; Fig. 3g), indicating that Light II cells retained correct Shh responsiveness. ROCK inhibition consistently increased Shh signalling in all knockdown conditions, even without SAG stimulation, and significantly increased Shh signalling in two of the three knockdown conditions: si*Ptch1 p* = 0.02834; si*Ift88 p* = 0.07092, si*Rpgrip1l p* = 0.01843 (Fig. 3g), suggesting that it can partially restore ciliary-mediated Shh signalling. As a second orthogonal assay, we also assessed Smo occupancy in the ciliary membrane. Smo accumulates in the ciliary membrane when PTCH1 is inactivated and is subsequently removed upon the binding of Shh ligand to PTCH1^45^. SAG treatment of mIMCD3 cells resulted in Smo accumulation within cilia (Fig. 3h), whereas si*Ift88* or si*Rpgrip1l* knockdowns reduced ciliary Smo levels (si*Ift88 p* = 0.001333, si*Rpgrip1l p* = 0.002788; Fig. 3i), indicating a significant decrease in Shh signalling. ROCK inhibition significantly increased Smo occupancy at cilia (*p* = 0.01940; Fig. 3i), suggesting that ROCK inhibition can restore aspects of ciliary function in a cellular model of the ciliopathy disease state.

### Secondary siRNA screening identified ROCK2 as a negative regulator of cilia incidence

**Fig. 4:**
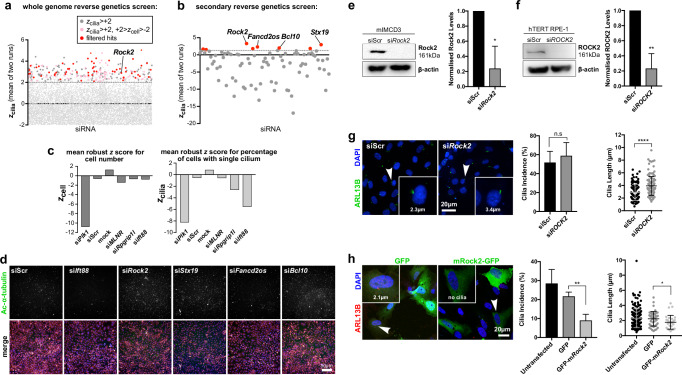
Whole genome siRNA-reverse genetics screen of cilia incidence identifies ROCK2 as a negative modulator of ciliogenesis. **a** Whole genome siRNA primary reverse genetics screen summarising mean *z*_cilia_ values (*n* = 2) for mouse mIMCD3 cells; 8907 data points are outside of the y-axis limit of −2.0. Dark grey points indicate hits *z*_cilia_ ≥ +2 (cut-off indicated by grey dashed line), and pink points indicate hits with no significant effect on cell number (−2 ≤ *z*_cell_ ≤ +2). Red points indicate 83 hits taken forward for secondary screening that have human orthologues and siRNAs targeting all transcripts of the gene. **b** Secondary screen (*n* = 2 biological replicates) of 83 primary screen hits in mIMCD3 cells, validating 8/83 hits (red points) with mean *z*_cilia_ ≥ +2. The top four hits are indicated with *z*_cilia_ values as follows: *Rock2* + 3.80, *Stx19* + 3.46, *Fancd2os* + 2.83, *Bcl10* + 2.49. **c** Bar graphs showing mean *z*_cell_ (left) and *z*_cilia_ (right) for positive controls (*Plk1*, *Ift88* and *Rpgrip1l* siRNAs) and negative control siRNAs (siScr and mock transfections) for the secondary screen (*n* = 2 biological replicates, 3 plates each, with 2 technical replicates for si*Plk1*, si*Mks1*, si*Rpgrip1l*, si*Ift88*, si*MLNR*, mock (transfection reagent only) and 4 technical replicates for siScr). **d** Representative high-content images from the primary screen for top hits (si*Rock2*, si*Stx19*, si*Fancd2os* and si*Bcl10* knockdowns) showing an increase in cilia incidence compared to scrambled siRNA (siScr) negative control; si*Ift88* is included as a positive control for cilia loss. Merge images show staining for cilia marker acetylated α-tubulin (green), nuclei (DAPI; blue) and cellular RNA (TOTO-3; pink). Scale bar = 50 µm. Validation of siRNA knockdowns for murine (**e**) and human (**f**) ROCK2 as a top candidate hit in mIMCD3 and hTERT RPE-1 cells compared to scrambled siRNA (siScr) negative control. Bar graphs quantify western blot densitometry measures (*n* = 3) for each cell line, normalised against β-actin levels, error bars represent s.d. Statistical significance of pairwise comparisons are indicated (*p = 0.0111, t = 4.472 and **p = 0.0025, t = 6.737, respectively, both unpaired, two-tailed Student’s t-tests, *n* = 3 biological replicates, df = 4). **g** Left: hTERT RPE-1 cells, stained for cilia marker ARL13B (green) after knockdown with si*ROCK2*, showing an increase in cilia length. Arrowheads indicate cilia displayed in magnified insets, shown with their measured lengths. Right: There was no significant difference in cilia incidence (n.s. *p* = 0.531, t = 0.6850; *n* = 3 biological replicates, df = 4). Mean cilium length after siScr treatment was 2.91 µm, compared to si*ROCK2* knockdown of 3.92 µm (*n* = 3 biological replicates, ≥40 cilia measured per replicate, specifically 121 and 132 cilia total for siScr and si*ROCK2* treated cells, respectively). ****p < 0.0001; t = 5.794, df = 251. Scale bar = 20 µm. **h** Left: hTERT RPE-1 cells transiently transfected with either untagged GFP or GFP-m*Rock2* constructs (green) and stained for ARL13B (red). Arrowheads indicate cells displayed in magnified insets. Right: cells transfected with GFP-m*Rock2* had a significantly lower cilia incidence (**p = 0.0049, t = 5.630; *n* = 3 biological replicates, df = 4) and shorter cilia (*p = 0.0120, Mann–Whitney *U*-test, *n* = 3 biological replicates (6 for untransfected) with a total of 188, 49 and 31 cilia measured for untransfected, GFP transfected (median length 2.362 µm) and GFP-mRock2 transfected (median 1.708 µm) cells, respectively, *U* = 506.5) compared to cells expressing untagged GFP. Scale bar = 20 µm.

Since fasudil and hydroxyfasudil inhibit both ROCK isozymes, ROCK1 and ROCK2, and have off-target effects on other kinases, we sought to determine the specific molecular drug targets that increase cilia incidence. We therefore re-analysed the raw image data from our previously published whole genome-wide siRNA-reverse genetics screen of ciliogenesis regulators that had identified hits that decreased cilia incidence^15^ to instead identify hits that increased cilia incidence. The screen data was filtered for hits that: (i) significantly increased cilia incidence *z*_cilia_ > +2; (ii) did not affect cell number +2 > *z*_cell_ > −2; (iii) used an siRNA pool that targeted all annotated transcripts of the gene; and (iv) had a human orthologue so that follow-up investigations could be carried out (Fig. 4a). We identified 83 hits from the re-analysed primary screen that were taken forward for secondary screening. Eight of 83 hits significantly increased cilia incidence across replicates of the secondary screen (Fig. 4b, Supplemental Data 2). Positive (si*Plk1*, si*Rpgrip1l* and si*Ift88*) and negative controls (siScr and mock transfections) verified that our assay could identify targets that caused changes in cell numbers and cilia incidence (Fig. 4c). The top hit from the secondary screen was *Rock2*, with an average *z*_cilia_ score of +3.309 and *z*_cell_ of −0.300, indicating increased cilia incidence (Fig. 4d). Efficient knockdown was confirmed by western blots (Fig. 4e, f)*. ROCK2* knockdown in hTERT RPE-1 cells increased cilia incidence by >10% and significantly increased average cilia length (by 34.7%; Fig. 4g). Over-expression of GFP-tagged m*Rock2* significantly decreased both cilia incidence and cilia length compared to controls (Fig. 4h).

We then assessed if the ROCK1 isozyme can compensate for ROCK2 during ciliogenesis since they both have distinct and separate functional roles in other cellular mechanisms^46–49^ but share 93% kinase domain homology. Efficient knockdowns by both mouse and human siRNAs against *ROCK1* were confirmed by western blots (Supplemental Fig. 2e). *ROCK1* knockdowns in either mIMCD3 or hTERT RPE-1 cells had no significant effects on cilia incidence (Supplemental Fig. 2f, g) but did increase cilia length in mIMCD3 cells. These findings all suggest that ROCK2 has a conserved and non-redundant role during mammalian ciliogenesis that is not fully compensated by ROCK1.

**Fig. 5:**
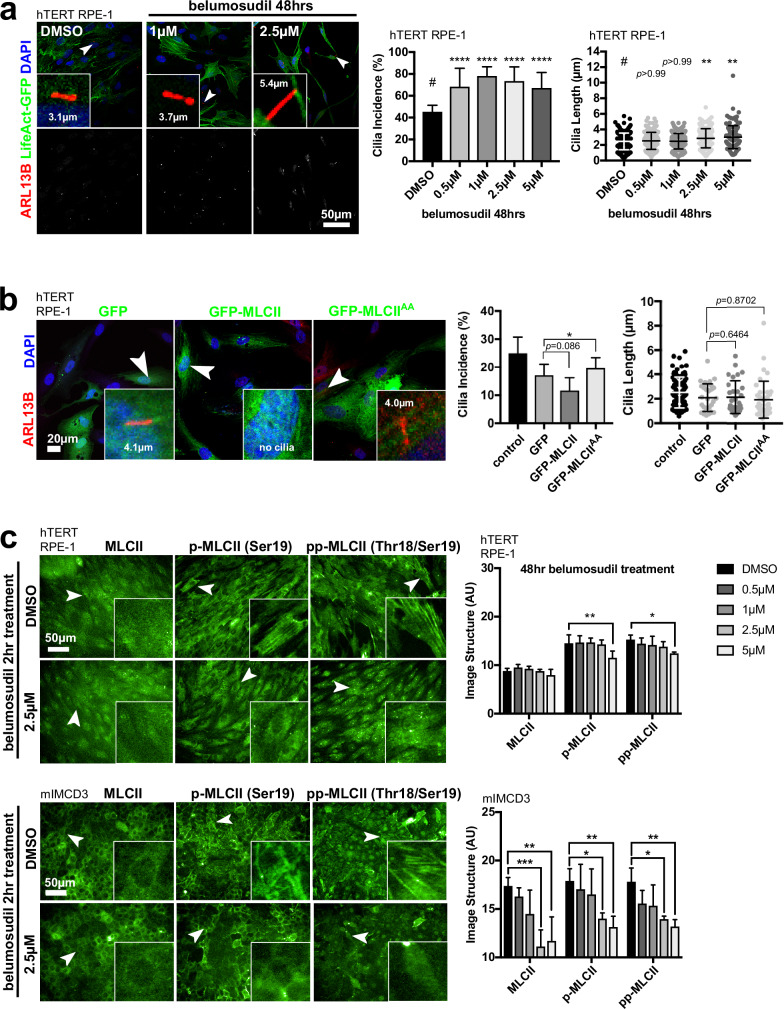
ROCK2 inhibition disrupts acto-myosin contraction and increases ciliogenesis. **a** Belumosudil treatment of hTERT RPE-1 cells for 48 h significantly increased cilia incidence across all concentrations (0.5–5 µM) ****p < 0.0001 for all comparisons (one-way ANOVA with Dunnett’s test for multiple corrections, *n* = 14 biological replicates, F = 14.49, df = 65) and had no effect on cilia length at lower concentrations, ns *p* > 0.9999 for both 0.5 µM and 1 µM belumosudil but increased cilia length for concentrations ≥2.5 µM compared to vehicle (DMSO) control, **p = 0.0087 and **0.0095, respectively for 2.5 µM and 5 µM belumosudil compared with DMSO vehicle control (Kruskal–Wallis test with Dunn’s multiple comparisons test, *n* = 3 biological replicates, ≥39 cilia measured per replicate, total cilia DMSO *n* = 212, 0.5 µM *n* = 280, 1 µM *n* = 271, 2.5 µM *n* = 167, 5 µM *n* = 119, Kruskal–Wallis statistic 20.12). The mean cilia length for the control treatment was 2.5 µm, compared to 2.9 µm and 3.0 µm at 2.5 µM and 5 µM belumosudil, respectively. **b** hTERT RPE-1 cells were transfected with GFP-tagged active mouse MLCII or constitutively inactive MLCII^AA^ constructs (green) and stained for ARL13B (red). Arrowheads indicate cells displayed in magnified insets. Cells that over-expressed active MLCII had a moderate but non-significant (ns) decrease in cilia incidence (ns *p* = 0.0866, t = 2.261, unpaired, two-tailed Student’s t-test, *n* = 3 biological replicates, df = 4), MLCII^AA^ expression significantly increased cilia incidence compared to untagged GFP control (*p = 0.0174, t = 3.908). There were no significant changes in cilia length when comparing transfection of GFP vs GFP-MLCII constructs (ns *p* = 0.8702, t = 0.1641, total cilia measured: control *n* = 97, GFP transfected *n* = 31, GFP-MLCII transfected *n* = 30, df = 59), nor when comparing GFP vs GFP-MLCII^AA^ (n.s. *p* = 0.6464, t = 0.4609, total cilia measured: GFP-MLCII^AA^ transfected *n* = 39, df = 68). **c** Belumosudil inhibits the kinase activity of ROCK2 and changes non-muscle myosin IIA organisation, visualised by MLCII staining and the presence of MLCII-associated acto-myosin structures in both hTERT RPE-1 (upper panels) and mIMCD3 (lower panels) cell lines. Cells were treated with increasing concentrations of belumosudil for either 2 h or 48 h (*n* = 3 for both time points). Antibodies marked MLCII, p-MLCII (monophosphorylated MLCII) and pp-MLCII (biphosphorylated MLCII at Thr18 & Ser19), with representative images shown with 2.5 µM belumosudil for 2 h treatments. p-MLCII and pp-MLCII visualise acto-myosin fibre-like structures in both hTERT RPE-1 (upper panels) and mIMCD3 (lower panels), visible in cells indicated by arrowheads and displayed in magnified insets. Automated high-content image analysis of image structure quantified both p-MLCII and pp-MLCII fibre-like structures, with bar graphs showing significant decreases in both hTERT RPE-1 (top) and mIMCD3 (bottom) cells when treated with 5 µM belumosudil for 48 h (For hTERT RPE cells: p-MLCII DMSO vs 5 µM belumosudil **p = 0.0087, pp-MLCII DMSO vs 5 µM belumosudil *p = 0.0127, both F = 6.607, df = 30. For mIMCD3 cells: MLCII DMSO vs 2.5 µM belumosudil ***p = 0.0003, DMSO vs 5 µM belumosudil **p = 0.0010, p-MLCII DMSO vs 2.5 µM belumosudil *p = 0.0280, DMSO vs 5 µM belumosudil **p = 0.0061, pp-MLCII DMSO vs 2.5 µM belumosudil *p = 0.0296, DMSO vs 5 µM belumosudil **p = 0.0077, all F = 14.56, df = 30, *n* = 3 biological replicates, two-way ANOVA with Dunnett’s multiple comparisons test). Scale bar = 50 µm.

To further validate this finding, we used the specific ROCK2 inhibitor belumosudil (also known as KD025 and SLX-2119), which has IC_50_ values of 24 µM and 0.105 µM for ROCK1 and ROCK2, respectively (Supplemental Table 1). Belumosudil treatment over a 2 h period had no effect on cilia incidence in mIMCD3 nor hTERT RPE-1 cells (Supplemental Fig. 5a). However, significant effects on both cilia incidence and cilia length were observed after 48 h treatment (Fig. 5a, Supplemental Fig. 5b). This suggested that the inhibition of ROCK2 was the predominant effect leading to increased ciliogenesis and rescue of ciliary function.

### Acto-myosin contraction contributes to the regulation of ciliogenesis

To substantiate the contribution of ROCK2, we first tested the effect of chemical inhibitors of F-actin stabilisation (cytochalasin D) and non-muscle myosin II (blebbistatin). Consistent with a previous study^40^, treatment with 0.5 µM cytochalasin D significantly increased cilia incidence over 16 h from 28.8 to 51.3%, and significantly increased cilia length in as little as 2 h by 11.7% (Supplemental Fig. 6a). However, 0.5 µM blebbistatin treatment also increased cilia incidence over 16 h from 25.3 to 44.7% (consistent with previous reports^50^) and 2.5 µM treatment significantly increased cilia length in 2 h by 20.9% (Supplemental Fig. 6b). These data suggest that short-term (2 h) treatments caused significant increases in cilia length, whereas longer 16 h treatments caused increases in cilia incidence. The increase in cilia incidence was more significant for treatment with blebbistatin, an inhibitor of acto-myosin contractions, than for cytochalasin D, which inhibits F-actin stabilisation, indicating the possible relative contributions of these processes during ciliogenesis.

We further validated the functional role of acto-myosin contractility during ciliogenesis by transfecting GFP-tagged wild-type mouse non-muscle myosin light chain II (MLCII) or a constitutively inactive non-phosphorylatable mutant of MLCII (MLCII^AA^) into hTERT RPE-1 cells^51^. Expression of wild-type GFP-MLCII did not significantly decrease cilia incidence or length, possibly because it was not readily phosphorylated by ROCK2. However, constitutively inactive GFP-MLCII^AA^ significantly increased cilia incidence, compared to a GFP-only transfection control (Fig. 5b), phenocopying blebbistatin treatment and *ROCK2* siRNA knockdowns. We confirmed by high-content imaging that *ROCK2* knockdowns, but not ROCK1 (Supplemental Fig. 3b), and belumosudil treatment (Fig. 5c) significantly reduced levels of active phosphorylated non-muscle MLCII and fibre-like patterns associated with acto-myosin contractility.

### Treatment of ciliopathy kidney organoids with ROCK inhibitors reduces cyst number and renal tubule size

**Fig. 6:**
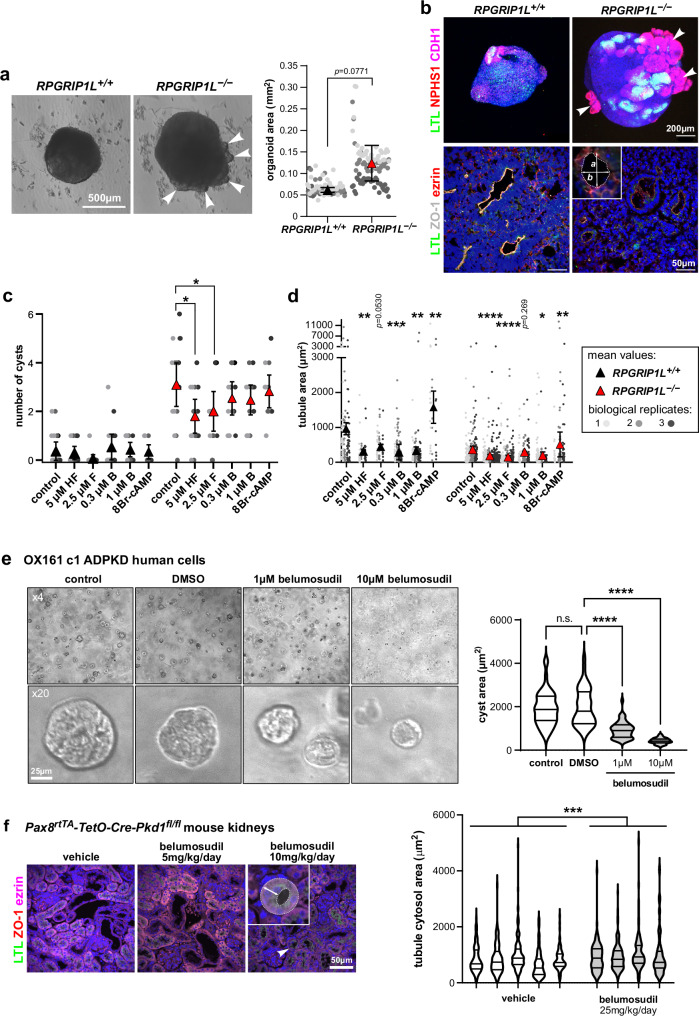
Hydroxyfasudil and belumosudil rescue normal renal tubule sizes for kidney organoids modelling severe ciliopathy and for an in vivo early-onset model of polycystic kidney disease. **a** Brightfield images of wild-type isogenic control (left) and *RPGRIP1L*^−^^/^^−^ mutant line 1 (right) kidney organoids at day 19 of differentiation. Arrowheads indicate cysts. Scale bar = 500 µm. The scatter graph shows the quantification of organoid area at day 19, based on automatic quantification of brightfield images. Triangles indicate the median values, error bars represent SD; unpaired two-tailed Student’s t-test n.s. *p* = 0.0771, t = 4.4248, df = 4, for *n* = 3 biological replicates, with 31, 32, 32 and 32, 32, 32 technical replicates, respectively (one set of technical replicates was for organoids grown from the same original well of stem cells). **b** Top: wild-type (left) and *RPGRIP1L*^−^^/^^−^ mutant line 1 kidney organoids (right) stained for LTL (proximal tubules; green), NPHS1 (nephron and podocytes; red) and CDH1 (distal tubules; magenta). Arrows indicate cysts. Scale bar = 200 µm. Bottom: kidney organoid sections stained for LTL (proximal tubules; green), ZO-1 (tight junctions; grey) and ezrin (apical cell surface; red). Scale bar = 50 µm. The inset indicates dimensions *a* and *b* used to calculate tubule area using the formula $\pi {ab}$. **c** Scatter graph showing cyst number observed in day 23 drug treated wild-type (left) and *RPGRIP1L*^−^^/^^−^ mutant line 1 (right) kidney organoids. The mean number of cysts before drug treatment at day 19 was 0.0538 for isogenic wild-type control and 1.846 for *RPGRIP1L*^−^^/^^−^ mutants. Significantly fewer cysts in mutant *RPGRIP1L*^−^^/^^−^ organoids were observed for DMSO vs 5 µM hydroxyfasudil (HF; unpaired two-tailed Student’s t-test: *p = 0.01059, t = 5.0499, df = 33, *n* = 21 and 14 for respective groups) and DMSO vs 2.5 µM fasudil (F; *p = 0.03711, t = 4.1848, df = 34, *n* = 21 and 15 for respective groups). **d** Tubule areas (median values) in sections of day 23 wild-type and *RPGRIP1L*^−^^/^^−^ mutant line 1 kidney organoids following drug treatments. Error bars indicate SD. Unpaired two-tailed Student’s t-tests for wild-type *RPGRIP1L*^+/+^ organoids: DMSO vs 5 µM hydroxyfasudil (HF) **p = 0.00512, t = 6.1384, df = 175, *n* = 126, 51, respectively; DMSO vs 2.5 µM fasudil (F) not significant (n.s.) *p* = 0.0530, t = 4.2866, df = 174, *n* = 126, 50, respectively; DMSO vs 0.3 µM belumosudil (B) ***p = 0.000858, t = 5.2481, df = 223, *n* = 126, 109, respectively; DMSO vs 1 µM belumosudil **p = 0.00302, df = 229, t = 5.9188, *n* = 126, 105, respectively; DMSO vs 8-bromo-cAMP **p = 0.00849, t = 6.8629, df = 155, *n* = 126, 31, respectively. For *RPGRIP1L*^−^^/^^−^ mutant line 1: DMSO vs 5 µM hydroxyfasudil (H) ****p = 0.0000383, t = 8.3237, df = 509, *n* = 208, 303, respectively; DMSO vs 2.5 µM fasudil (F) ****p = 0.0000256, t = 8.3519, df = 429, *n* = 208, 223, respectively; DMSO vs 0.3 µM belumosudil (B) n.s. *p* = 0.269, t = 2.3125, df = 320, *n* = 208, 114, respectively; DMSO vs 1 µM belumosudil (B) *p = 0.0132, t = 5.1269, df = 327, *n* = 208, 121, respectively; DMSO vs 8-bromo-cAMP **p = 0.00592, t = 5.6543 df = 301, *n* = 208, 95, respectively. **e** The ROCK2 inhibitor belumosudil reduced cyst growth in a patient-derived *PKD1* cystic cell line (OX161/c1) in 3D Matrigel cyst assays. Representative images of cysts after 12 days of treatment. The average cyst area was reduced at all concentrations tested (*n* = 3, *n* = 50, 44, 48, 48 cysts per treatment, respectively). Significance was determined by one-way ANOVA (Control vs DMSO: ns *p* = 0.9879, DMSO vs 1 µM belumosudil: ****p < 0.0001, DMSO vs 10 µM belumosudil: ****p < 0.0001, all df = 218, F = 71.71). **f** Left: kidney sections from *Pax8*^*rtTA*^*-TetO-Cre-Pkd1*^*fl/fl*^ mice stained for LTL (proximal tubules; green), ZO-1 (tight junctions; red) and ezrin (apical cell surface; red) following drug treatments with vehicle or belumosudil at either 5 mg/kg/day or 10 mg/kg/day (representative images from an experiment with *n* = 5 animals for each treatment, *n* = 15 animals total). Scale bar = 50 µm. The inset indicates the tubule area, delimited by ezrin and ZO-1 staining for LTL^+^ tubules, used in calculations. Right: Violin plot of individual renal tubule area per kidney for 25 mg/kg/day drug treatment (*n* = 5 or 4 animals, as indicated (*n* = 9 animals total), with *n* = 620 and 422 tubules total, respectively), showing increase in tubule cytosol areas (unpaired two-tailed Student’s t-tests for tubules from vehicle vs 25 mg/kg/day belumosudil animal groups, ***p = 0.0002243. t = 8.4978, df = 1040).

Next, we investigated the effect of ROCK inhibition in a physiologically relevant 3D kidney organoid ciliopathy disease model. We used CRISPR-Cas9 gene editing to introduce biallelic *RPGRIP1L* exon 2 mutations (Supplemental Fig. 7a, b) into human induced pluripotent stem cells (iPSCs; line AD2)^16^. RPGRIP1L mutant lines and normal isogenic control iPSCs were pluripotent (Supplemental Fig. 7c), with mutant lines having greater dose-dependent increases in cilia incidence than the isogenic control line in response to both fasudil and hydroxyfasudil (Supplemental Fig. 7d). We then differentiated iPSC lines into kidney precursor cells and then kidney organoids in non-adherent 96-well plates^25,26^. Line 1 *RPGRIP1L* mutant kidney organoids were moderately larger than normal isogenic controls (*p* = 0.0771; Fig. 6a) and had significantly more visible cystic changes (mean cyst number: 0.0538 for wild-type vs 1.846 for *RPGRIP1L*^−^^/^^−^ mutant, Welch’s unpaired t-test *p* = 0.0194 t = 6.400, df = 2.161, *n* = 3 biological replicates (organoids derived from the same well of stem cells) each with 31–32 organoids). This confirmed that biallelic mutations in *RPGRIP1L* caused significant cystic changes in mature kidney organoids.

At day 20, mature kidney organoids were treated for 4 days with a ROCK inhibitor (hydroxyfasudil, fasudil or belumosudil), 8-Br-cAMP (a cAMP analogue to induce cystogenesis as a positive control) or vehicle control (0.1% DMSO). All kidney organoids continued to express mature kidney markers of kidney proximal tubule, distal tubule and nephron structures during these treatments (Fig. 6b). *RPGRIP1L*^−^^/^^−^ mutant organoids treated with 5 µM hydroxyfasudil and 2.5 µM fasudil had significantly fewer cysts (*p* = 0.01059 and *p* = 0.03711, respectively; Fig. 6c), whereas cyst number remained unchanged for control organoids treated with ROCK inhibitors or 8-Br-cAMP. However, individual tubule areas increased in both control and *RPGRIP1L*^−^^/^^−^ mutant organoids treated with 8-Br-cAMP (*p* = 0.00849 and *p* = 0.00592, respectively), consistent with a pre-cystic change in tissue organisation (Fig. 6d). For both control and *RPGRIP1L*^−^^/^^−^ mutant organoids, kidney tubules were significantly smaller following treatment with the majority of ROCK inhibitors, including belumosudil at two different doses (0.3 and 1.0 µM; Fig. 6d). *RPGRIP1L*^−^^/^^−^ mutant organoids had disrupted apico-basal tissue organisation within renal tubules, as visualised by the apical cell surface marker ezrin and the tight junction protein ZO-1 (Fig. 6b, Supplemental Fig. 8a). Treatment with 5 µM hydroxyfasudil, but not 1 µM belumosudil, increased levels of normally localised ZO-1 in *RPGRIP1L*^−^^/^^−^ (*p* = 0.0164; Supplemental Fig. 8b). These results suggest that ROCK inhibitors may help to prevent or reduce pre-cystic changes in tissue organisation in kidney organoids modelling the ciliopathy disease state.

### ROCK2 inhibition in models of polycystic kidney disease reduces cysts and increases renal tubule wall size

We next assessed if ROCK2 inhibition could have potential therapeutic benefit for the most common ciliopathy, autosomal dominant polycystic kidney disease (PKD). Since the ROCK inhibitor hydroxyfasudil reduced cyst expansion in both human *PKD1* 3D cyst assays and an inducible *Pkd1* mouse model^27^, we determined the effect of the ROCK2-specific inhibitor belumosudil in these assays. Belumosudil treatment (1 and 10 μM, 12 days) was associated with a significant decrease in cyst area in the patient-derived *PKD1* cystic line (OX161/c1) (Fig. 6e). We next tested the effectiveness of belumosudil in a doxycycline-inducible kidney-specific *Pkd1* mouse model (*Pax8*^*rtTA*^*-TetO-Cre-Pkd1*^*fl/fl*^)^28^. Treatment with belumosudil (5 or 10 mg/kg/day) from postnatal days PN16-PN23 after kidney-specific *Pkd1* deletion at PN13 was well-tolerated, as reflected by changes in daily body weights (Supplemental Fig. 8c). As expected, ROCK2 inhibition reduced endogenous levels of phosphorylated non-muscle myosin light chain II (p-MLCII; Supplemental Fig. 8d, e), although the extent was variable between animals. After 7 days, drug treated mice had no obvious changes in fractional kidney cystic indices compared with the vehicle-treated control group (Supplemental Fig. 8e), and marginally significant but variable effects on tubule wall area (Supplemental Fig. 8e) at lower belumosudil doses, consistent with the variability in functional ROCK2 inhibition in vivo for these animals. However, at a higher dose (25 mg/kg/day belumosudil) renal tubule wall area in the *Pkd1* mouse model were significantly preserved compared to vehicle treated controls (*p* = 0.0002243, Fig. 6f). We suggest that this preservation of cell morphology might make treated tubules more resistant to subsequent cystic changes.

## Discussion

In this study, two hypothesis-free approaches highlight ROCK2 as a potential therapeutic target for the restoration of cilia in ciliopathy patients. A small molecule screen of FDA-approved drugs identifies fasudil, a broadly selective ROCK inhibitor, as a chemical that rescues ciliogenesis after knockdown or mutation of two known ciliopathy genes (*IFT88* and *RPGRIP1L*) in different cellular disease models but not in *TMEM67*. Increased ciliogenesis due to ROCK inhibition is likely due to increased ciliary vesicle trafficking and associated rescue of ciliary-mediated Sonic Hedgehog signalling. An independent whole genome reverse genetics screen and subsequent validation experiments identify ROCK2 as the target protein that mediates a strong negative regulation of cilia incidence and length. Functional studies verify that ROCK2 mediates ciliogenesis through the regulation of acto-myosin contractility. ROCK2 inhibition in kidney organoids that model ciliopathies, as well as in a patient-derived cystic cell and a mouse model that model PKD, suggest that preservation of tubule cell morphology and subsequent abrogation of cystic changes is a generalisable therapeutic strategy that could inhibit renal cyst initiation in the ciliopathy disease state.

ROCK2 has not been directly shown to be a negative regulator of ciliogenesis in previous studies, but downstream actin remodelling pathways activated by ROCK have provided indirect evidence for the importance of actin remodelling in controlling cilia incidence and length^40,41,52^. Thus, the current evidence suggests that a highly complex network of pathways and regulators control the functional contribution of the actin cytoskeleton during ciliogenesis. When activated by RhoA-GTP, both ROCK1 and ROCK2 phosphorylate several proteins and thus activate downstream pathways that regulate actin remodelling and dynamics. They have therefore been intensively studied to determine their roles in cell adhesion^53,54^, stress-fibre and focal adhesion formation^55,56^, and cell motility/migration^57^.

ROCK controls these cellular mechanisms by directly phosphorylating key actin regulators. ROCK phosphorylates LIMK2, which in turn phosphorylates cofilin to modulate F-actin stabilisation, as well as MLCII and MYPT1, which control acto-myosin contraction and the formation of stress fibres. ROCK also phosphorylates EZR, regulating the linking of the actin cytoskeleton to the plasma membrane^58^. Despite the overall sequence similarity of the two ROCK isozymes (65% overall, 93% in the kinase domain)^59^, our findings indicate that ROCK2 is the target molecule that mediates ciliogenesis rather than ROCK1. *ROCK1* knockdown does not phenocopy *ROCK2* knockdown, and residual ROCK1 activity cannot compensate for the chemical inhibition of ROCK2 kinase activity. These results agree with previous findings from knockout mice, which showed that ROCK1 and ROCK2 have distinct roles and cannot compensate for one another^60,61^, although both ROCK1 and ROCK2 are ubiquitously expressed^62–64^.

ROCK1 and ROCK2, therefore, have different roles in the regulation of the cytoskeleton^46^. Whilst ROCK1 is essential for the formation of stress fibres^65^, ROCK2 is reported to regulate F-actin stability through direct phosphorylation of both LIMK2 and MLCII, the regulatory subunit of non-muscle myosin IIA, leading to activation of acto-myosin cell contractility^66^. Local activation of apical acto-myosin contractility controls cell adhesion, polarity and morphogenesis^67^. In particular, acto-myosin contractility is required for basal body migration to the apical cell surface^50^, an essential mechanical event required prior to ciliogenesis. Regulation of cytoskeletal dynamics is required for both basal body docking and ciliary vesicle trafficking and is, therefore, an essential process of early ciliogenesis, axoneme elongation and maintenance^40,68–70^, although the molecular details remain unclear.

Fasudil was one of the first ROCK inhibitors identified^71^ and is a class I ATP competitive kinase inhibitor of both ROCK isozymes (Supplemental Table 2). Laboratory and clinical studies have shown the potential efficacy of first-generation ROCK inhibitors in treating other conditions such as cerebral vasospasm, glaucoma and idiopathic pulmonary fibrosis^72^. However, these compounds have only rarely entered licensed clinical use because kinase profiling indicates they have off-target effects and inhibit both ROCK isozymes. The pharmacological effect of earlier ROCK inhibitors is therefore complicated by the potentially undesirable effects of ROCK1 inhibition (which include reduced blood pressure, increased heart rate and reversible reduction in lymphocyte counts)^72,73^.

More recent isozyme-specific inhibitors have been formulated, such as belumosudil (also known as KD025)^74–76^ and many others^77^. Belumosudil (trade name REZUROCK) is a preferential ROCK2 inhibitor, developed as an investigational drug and used in the ROCKstar Study^78^, and is currently FDA-approved for treatment of chronic graft-versus-host disease. Despite this, it is notable that isozyme-specific inhibitors are often missing from drug screening libraries and remain commercially unavailable^52^. Lack of screening of specific ROCK1 or ROCK2 inhibitors may therefore mask inhibition of one or other isozyme as an effective target for other disease indications^52^.

Other studies have identified ROCK inhibitors as potential therapeutics for cystic kidney disease (reviewed in Smith et al. ^52^) in both ciliopathies and polycystic kidney disease (PKD). In agreement with our findings, an early study showed that the non-selective ROCK inhibitor Y27632 caused an increase in cilia length due to reductions in actin stress-fibre formation^79^. ROCK inhibitors restored ciliogenesis in a Rho GTPase activating protein (GAP) mutant mouse model (*Arhgap35*^D34/D34^) that had reduced cilia incidence and length^80^. Hydroxyfasudil, a broadly selective inhibitor of both isozymes, reduced cyst expansion in both human *PKD1* mutant 3D cyst assays and an inducible *Pkd1*^−^^*/*^^−^ mouse model^27^. ROCK inhibitors, including one selective for ROCK2, restored renal tubule formation whilst reducing cyst formation in a cystogenic mIMCD3 *Pkd1*^−^^/^^−^ 3D kidney model^81^. This suggests that ROCK2, and not ROCK1, is the key target isozyme, again in agreement with our findings.

In addition, our findings suggest that ROCK2 inhibition both increases cilia incidence and lengthens cilia, potentially increasing both chemosensory and mechanosensory signalling capacity. We have shown that ROCK2 inhibition is able to increase Shh signalling capacity as a functional output, but further research will be required to investigate the effects of these drugs on cilia length in animal and organoid disease models.

In conclusion, our work suggests that ROCK2 is a key mediator of cilium formation and function and determines that ROCK2 inhibition has a broad effect in rescuing ciliogenesis and ciliary function over several ciliopathy disease classes. We propose that ROCK2 inhibition represents a novel, disease-modifying therapeutic approach for heterogeneous ciliopathies, including PKD. Since the newer, more selective ROCK2 inhibitors may be more promising alternatives for ciliopathy disease indications, future work should focus on preclinical studies of belumosudil or related isoquinolone derivatives as potential candidates for drug repurposing.

## Supplementary information

Supplemental Information

Description of Additional Supplementary File

Supplemental Data 1

Supplemental Data 2

Supplemental Data 3

Supplemental Data 4

Supplemental Data 5

Supplemental Data 6

reporting summary

## Data Availability

CRISPR-Cas9 edited cell lines are available upon request. The authors declare that the data supporting the findings of this study are available within the paper and its supplementary information files. The source data for Figs. 1e and 2a–d can be found in Supplemental Data 1 and Supplemental Data 4. The source data for Figs. 2e, f, 3b–d, f, g, i, 4b, c, f–h, 5a–c, 6a, c–f and Supplemental Figs. 1a–c, 2e–g, 3a, 3b, 4, 5a, 5b, 6a, 6b, 7d, 8a–c, 8e can be found in Supplemental Data 4. The source data for Fig. 4e and Supplemental Figs. 2a–c, 7b can be found in Supplemental Data 4 and 5. The source data for Supplemental Fig. 8d can be found in Supplemental Data 5. Sanger sequencing data can be found in Supplemental Data 6. Previously published experimental data that this study presents in Fig. 4a is available in the referenced open access publication^15^. Source data presented in Fig. 4c is available from the publically available repository https://etheses.whiterose.ac.uk/27888/.
