# Supplementary material for: Drug and siRNA screens identify ROCK2 as a therapeutic target for ciliopathies
Source: Commun Med (Lond). 2025 Apr 19;5:129. doi: 10.1038/s43856-025-00847-1 (PMC12009310; doi:10.1038/s43856-025-00847-1)
Supplement: Supplementary file 1 — Supplemental Information [file 43856_2025_847_MOESM1_ESM.pdf]

Supplemental Data: Drug and siRNA screens identify ROCK2 as a therapeutic target for ciliopathies

Claire E. L. Smith[1*], Andrew J. Streets[2*], Alice V. R. Lake[1*], Subaashini Natarajan[1], Sunayna K. Best[1], Katarzyna Szymanska[1], Magdalena Karwatka[1], Thomas Stevenson[3], Rachel Trowbridge[4], Gary Grant[1], Sushma N. Grellscheid[3,5], Richard Foster[4,6], Ciaran G Morrison[7], Georgia Mavria[1], Jacquelyn Bond[8], Albert C. M. Ong[2] and Colin A. Johnson[1]

[1] Division of Molecular Medicine, Leeds Institute of Medical Research, Faculty of Medicine and Health, University of Leeds. Leeds. LS9 7TF. UK.

[2] Academic Nephrology Unit, Division of Clinical Medicine, School of Medicine and Population Health, Faculty of Health, Sheffield. S10 2HQ. UK.

[3] Computational Biology Unit, Department of Biosciences, Thormøhlensgt, 53 A/B, University of Bergen, 5020 Bergen, Norway.

[4] School of Molecular & Cellular Biology & Astbury Centre for Structural Molecular Biology, Faculty of Biological Sciences, University of Leeds, Leeds. LS2 9JT. UK.

[5] Department of Biosciences, University of Durham. DH1 3LE. UK.

[6] School of Chemistry, University of Leeds, Leeds. LS2 9JT. UK.

[7] Centre for Chromosome Biology, School of Biological and Chemical Sciences, University of Galway, Galway, H91 W2TY. Ireland.

[8] St James's Campus Infrastructure Flow Cytometry and Imaging Facility, Faculty of Medicine and Health, University of Leeds, St James's University Hospital, Leeds. LS9 7TF. UK

* indicates joint first authorship, these authors contributed equally to this work.

Corresponding author: c.johnson@leeds.ac.uk +44 113 343 8443

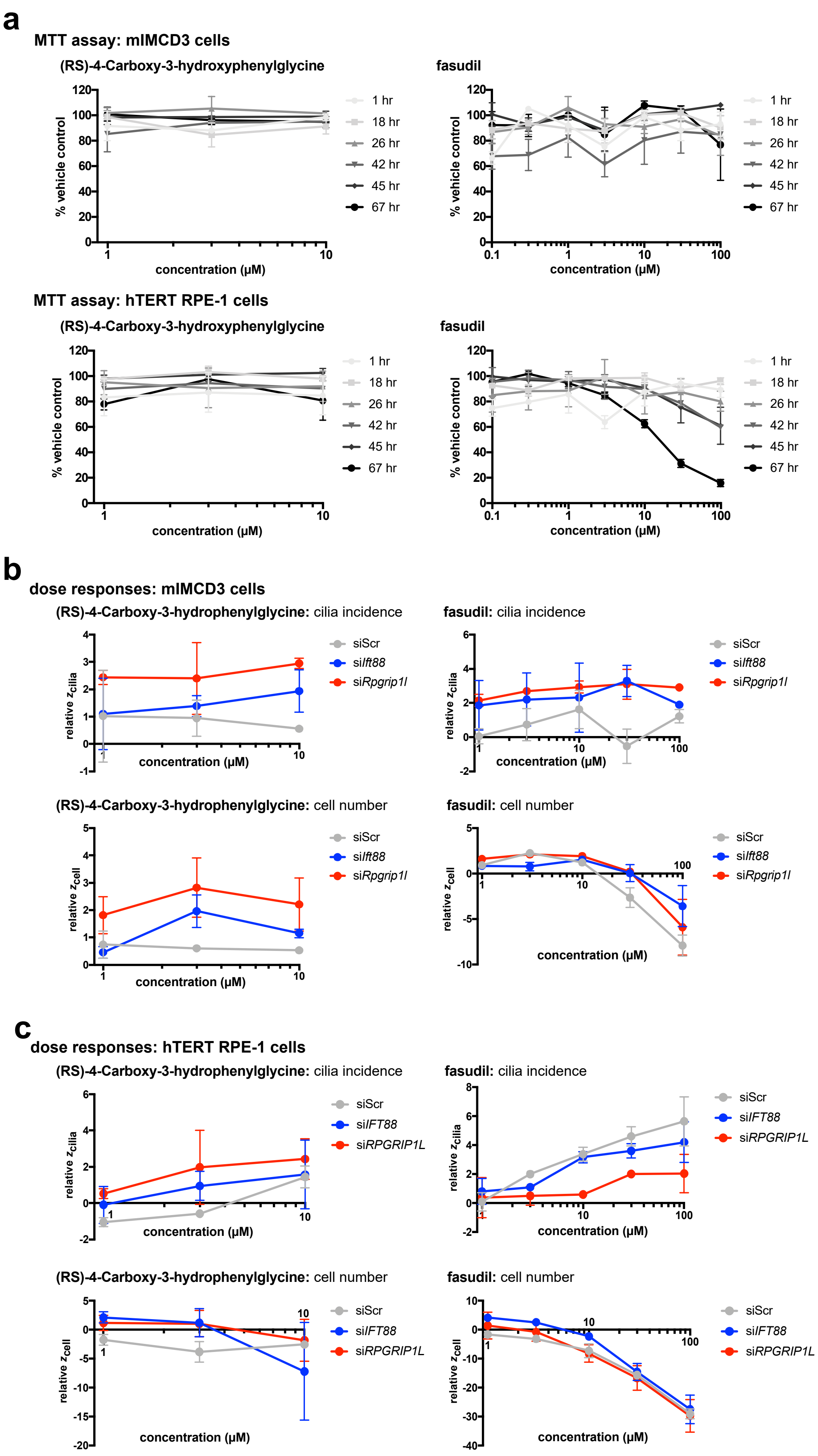
a
MTT assay: mIMCD3 cells
(RS)-4-Carboxy-3-hydroxyphenylglycine
fasudil
% vehicle control
concentration (µM)
1 hr
18 hr
26 hr
42 hr
45 hr
67 hr
MTT assay: hTERT RPE-1 cells
(RS)-4-Carboxy-3-hydroxyphenylglycine
fasudil
b
dose responses: mIMCD3 cells
(RS)-4-Carboxy-3-hydrophenylglycine: cilia incidence
fasudil: cilia incidence
relative z cilia
siScr
siIft88
siRpgrip1l
(RS)-4-Carboxy-3-hydrophenylglycine: cell number
fasudil: cell number
relative z cell
c
dose responses: hTERT RPE-1 cells
(RS)-4-Carboxy-3-hydrophenylglycine: cilia incidence
fasudil: cilia incidence
siIFT88
siRPGRIP1L
(RS)-4-Carboxy-3-hydrophenylglycine: cell number
fasudil: cell number

Supplemental Figure 1: Fasudil dose response and cell viability assays in ciliopathy gene knockdown hTERT RPE-1 cells

(a) Dose response MTT assays of cell viability in mIMCD3 (upper two panels) or hTERT RPE-1 cells (lower two panels) treated with either (RS)-4-carboxy-3-hydrophenylglycine (left) or fasudil (right). Values are normalised to vehicle-treated control cells at each timepoint (1 to 67 hr treatments) for n=2 experimental replicates, each with n=3 technical replicates. Error bars represent range. **(b)** Dose response assays in mIMCD3 cells with bar graphs showing z_{cilia} (top panels) and z_{cell} (bottom panels) values relative to vehicle (DMSO) treated cells specific to each knockdown condition, for either (RS)-4-carboxy-3-hydrophenylglycine (left) or fasudil (right). Concentration range for (RS)-4-carboxy-3-hydrophenylglycine was 1 to 10 μM due to limited solubility in DMSO; fasudil was tested 1 to 100 μM. Cells were reverse transfected with siScr control (grey), si*Ift88* (blue) or si*Rpgrip1l* (red), as indicated. Values are for n=2 experimental replicates, each with n=3 technical replicates. Error bars represent range. Significant increases / decreases are denoted by values > 2 / < -2. **(c)** Dose response assays in hTERT RPE-1 cells with bar graphs showing z_{cilia} (top panels) and z_{cell} (bottom panels) values relative to vehicle (DMSO) treated cells specific to each knockdown condition for either (RS)-4-carboxy-3-hydrophenylglycine (left) or fasudil (right). Concentration range for (RS)-4-carboxy-3-hydrophenylglycine was 1 to 10 μM due to limited solubility in DMSO; fasudil was tested 1 to 100 μM. Cells were reverse transfected with siScr control (grey), si*IFT88* (blue) or si*RPGRIP1L* (red), as indicated. Values are for n=2 experimental replicates, each with n=3 technical replicates. Error bars represent range. Significant increases / decreases are denoted by values > 2 / < -2.

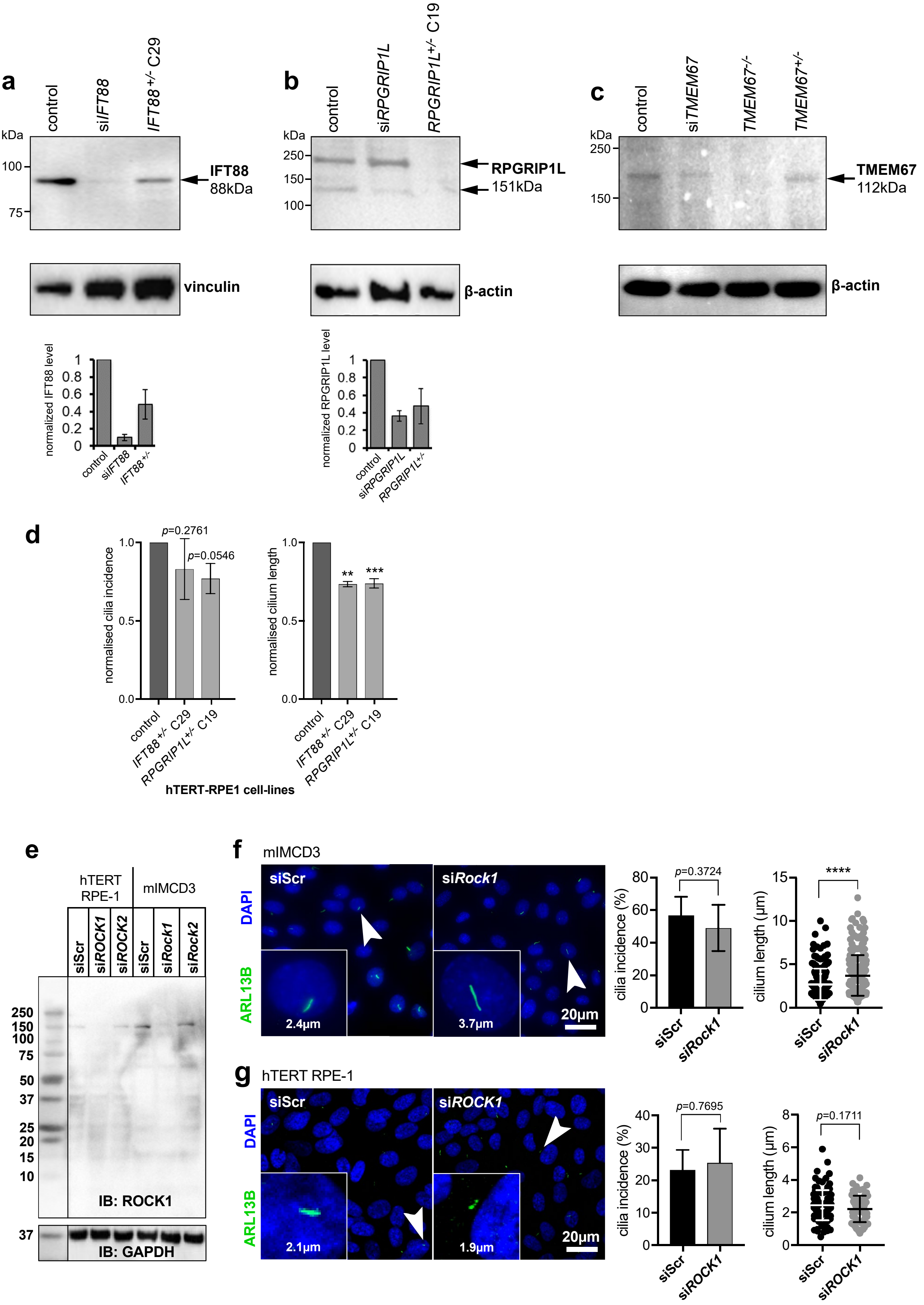

a
control
siIFT88
IFT88+/- C29
kDa
100
75
IFT88
88kDa
vinculin
normalized IFT88 level
1
0.8
0.6
0.4
0.2
0
control
siIFT88
IFT88+/-
b
control
siRPGRIP1L
RPGRIP1L+/- C19
kDa
250
150
100
RPGRIP1L
151kDa
β-actin
normalized RPGRIP1L level
control
siRPGRIP1L
RPGRIP1L+/-
c
control
siTMEM67
TMEM67-/-
TMEM67+/-
kDa
250
150
TMEM67
112kDa
β-actin
d
normalised cilia incidence
p=0.2761
p=0.0546
1.0
0.5
0.0
control
IFT88+/- C29
RPGRIP1L+/- C19
normalised cilium length
**
***
hTERT-RPE1 cell-lines
e
hTERT RPE-1
mIMCD3
siScr
siROCK1
siROCK2
siScr
siRock1
siRock2
250
150
100
75
50
37
25
20
15
10
IB: ROCK1
37
IB: GAPDH
f
mIMCD3
siScr
siRock1
DAPI
ARL13B
2.4µm
3.7µm
20µm
cilia incidence (%)
p=0.3724
80
60
40
20
0
siScr
siRock1
cilium length (µm)
****
15
10
5
0
g
hTERT RPE-1
siScr
siROCK1
2.1µm
1.9µm
20µm
p=0.7695
40
30
20
10
p=0.1711
8
6
4
2
siScr
siROCK1

Supplemental Figure 2: Assessment of relevant protein levels in CRISPR Cas9 edited cell lines and *ROCK1* knockdown does not significantly modulate ciliogenesis.

(a) Western blot showing the relative IFT88 protein levels in hTERT RPE-1 cells transfected with siScr or si*IFT88* or CRISPR-Cas9 engineered heterozygous knockout *IFT88* ((NM_175605.5):[c.371_408delinsAAGAAAAAAG,p.(P124Qfs*15)]) hTERT RPE-1 cells. An arrow indicates the bands used for quantification (predicted weight 88 kDa). Protein levels were quantified using densitometry relative to vinculin (n=3 experimental replicates). Protein levels were significantly decreased for cells transfected with si*IFT88* and for *IFT88*$^{+/-}$ hTERT RPE-1 cells compared with the siScr control. Student's unpaired, two-tailed t test: siScr vs si*IFT88* **** p<0.0001, t=4.4529, siScr vs *IFT88*$^{+/-}$ hTERT RPE-1 * p=0.01253, t=4.0570, both n=3 df=4. **(b)** Western blot showing the relative RPGRIP1L protein levels in hTERT RPE-1 cells transfected with siScr (control) or si*RPGRIP1L* or CRISPR-Cas9 engineered heterozygous knockout *RPGRIP1L* ((NM_015272.5):[c.15_37del, p.(D6Cfs*35)]) hTERT RPE-1 cells. Arrows indicate the bands that represent the two main protein isoforms detected (predicted weight 151 kDa). The lower bands were used for quantification. Protein levels were quantified using densitometry relative to β-actin (n=3 experimental replicates). Protein levels were significantly decreased for cells transfected with si*RPGRIP1L* and for *RPGRIP1L*$^{+/-}$ hTERT RPE-1 cells compared with the siScr control. Student's unpaired, two-tailed t test: siScr vs si*RPGRIP1L* **** p<0.0001, t=4.4464, siScr vs *RPGRIP1L*$^{+/-}$ hTERT RPE-1 * p=0.01046, t=4.0933, both n=3 df=4. **(c)** Western blot showing the relative TMEM67 protein levels in hTERT RPE-1 cells of cells transfected with siScr (control) or si*TMEM67* or CRISPR-Cas9 engineered heterozygous knockout *TMEM67* ((NM_153704.6): [c.369delC, p.(E124Kfs*12)]) and homozygous knockout *TMEM67* ((NM_153704.6):[c.519delT, p.(C173Wfs*20)];[c.519dupT, p.(E174*)]) hTERT RPE-1 cells. The bands detected were of a larger weight than was expected (predicted weight detected by antibody was 112 kDa), possibly due to post translational modifications. Further investigation using high content imaging indicated a reduction in cilia incidence compared with the WT parent line for the CRISPR-Cas9 engineered *TMEM67*$^{+/-}$ and the *TMEM67*$^{-/-}$ lines of 59% and 89% respectively (data available on request). **(d)** Normalised cilia incidence and cilium length data for the

IFT88$^{+/-}$ and *RPGRIP1L*$^{+/-}$ hTERT RPE-1 cell lines. Cilium membrane area (measured as spot size in pixels squared (px^2)) was used as a proxy for cilium length All measurements were normalised to the mean values for WT hTERT RPE-1. Bar graph (left) shows a non-significant decrease in cilia incidence for *IFT88*$^{+/-}$ and a near significant decrease in *RPGRIP1L*$^{+/-}$ hTERT RPE-1 cell lines when compared with WT hTERT RPE-1 cells. Data was confirmed to be normally distributed using a D'Agostino & Pearson omnibus K2 test. Significance was calculated using One-way ANOVA with Dunnett's multiple comparisons test WT hTERT RPE-1 vs. *IFT88*$^{+/-}$ ns p=0.2761; WT hTERT RPE-1 vs. *RPGRIP1L*$^{+/-}$ ns p=0.0546, both F=11.97, df=44, with a minimum of 3 biological replicates for each genotype: 8 for WT hTERT RPE-1, 3 for *IFT88*$^{+/-}$ and 4 for *RPGRIP1L*$^{+/-}$. Bar graph (right) shows a significant decrease in cilium length for both cell lines when compared to WT hTERT RPE-1 cells. Data was confirmed to be normally distributed using a D'Agostino & Pearson omnibus K2 test. Significance was calculated using One-way ANOVA with Dunnett's multiple comparisons test WT hTERT RPE-1 vs. *IFT88*$^{+/-}$ ** p=0.0019; WT hTERT RPE-1 vs. *RPGRIP1L*$^{+/-}$ *** p=0.00023, both F=21.36, df=35, with a minimum of 8 biological replicates for each genotype: 32 for WT hTERT RPE-1, 8 for *IFT88*$^{+/-}$ and 8 for *RPGRIP1L*$^{+/-}$. Error bars represent the standard deviation on both graphs. **(e)** Specificity of *ROCK1* siRNAs demonstrated by western blots following siRNAs knockdowns of either human *ROCK1* or mouse *Rock1* (expected molecular weight 158 kDa). GAPDH is the loading control. **(f)** Representative confocal microscopy images of mIMCD3 cells knocked down with si*Rock1* or siScr negative control and stained for cilia marker ARL13B (green). Representative examples of cilia length measurement are shown for each condition. Bar graphs show a slight decrease in cilia incidence following si*Rock1* knockdown (n.s. p=0.3724, t=0.9449 n=5 biological replicates, df=8, unpaired, two-tailed Student's t-test) but a significant increase in mean cilia length from 2.7 μm to 3.7 μm (**** p<0.0001, n=3 biological replicates, with n=181, median 2.151 μm and n=234, median 2.778 μm respectively for siScr and si*Rock1* transfected cells, U statistic=14887, Mann-Whitney U-test). **(g)** Representative confocal microscopy images of hTERT RPE-1 cells treated with si*ROCK1* or siScr negative control as for (e). Bar graphs show a moderate but not significant increase in cilia incidence following si*ROCK1* knockdown (n.s. p=0.7695, t=0.3136, df=4, n=3 biological replicates).

Mean cilia length was decreased following si*ROCK1* knockdown, from 2.4 μM to 2.2 μM (n.s. *p*=0.1711, n=3 biological replicates, with n=106, median 2.438 μm and n=72, median 2.217 μm respectively for siScr and si*Rock1* transfected cells, *U* statistic=3354, Mann-Whitney *U*-test).

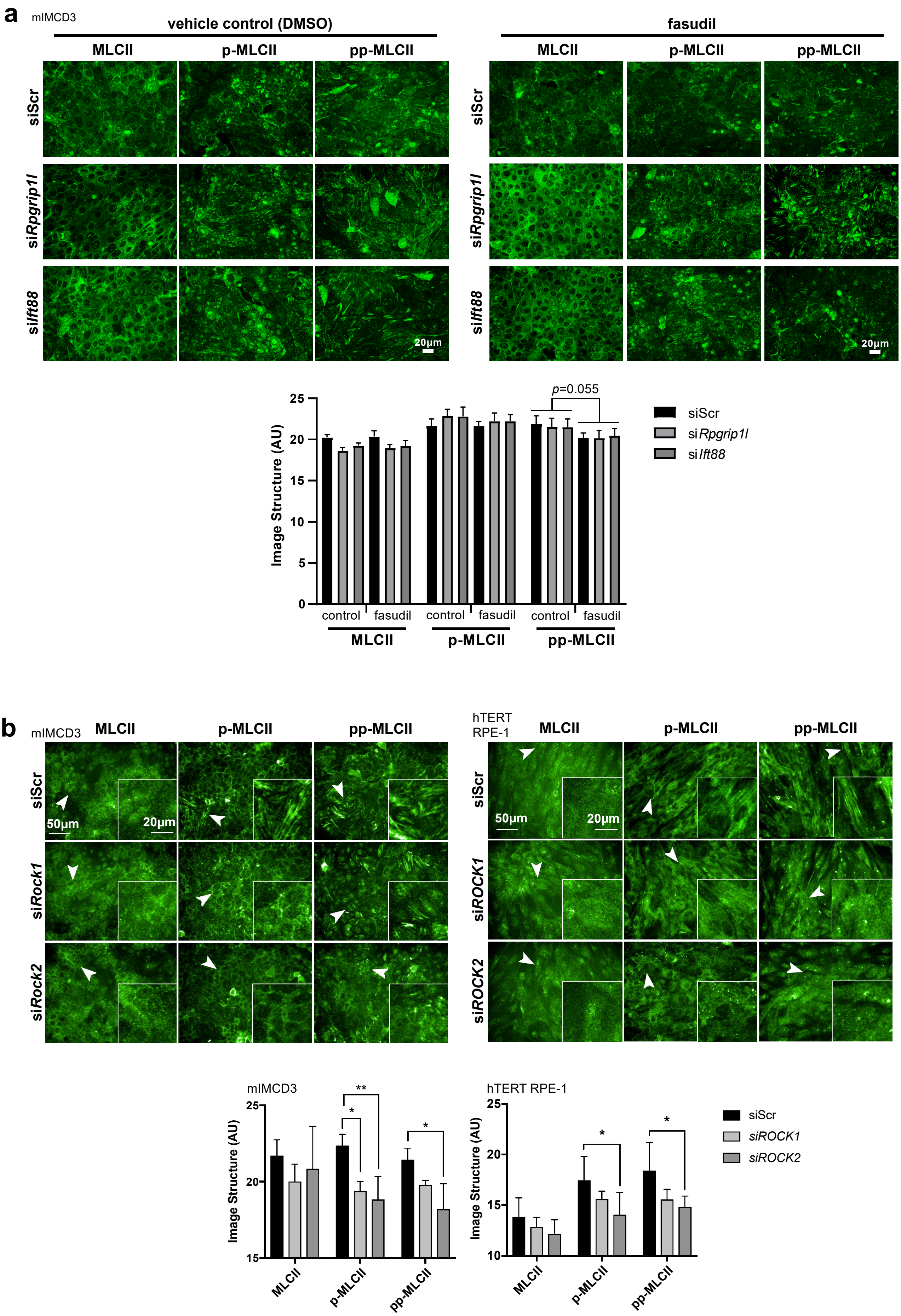

a
mIMCD3
vehicle control (DMSO)
fasudil
MLCII
p-MLCII
pp-MLCII
siScr
siRpgrip1l
siIft88
20μm
p=0.055
Image Structure (AU)
control
fasudil
siScr
siRpgrip1l
siIft88
b
mIMCD3
hTERT RPE-1
siRock1
siRock2
siROCK1
siROCK2
50μm
20μm

Supplemental Figure 3: *ROCK2* knockdown, but not fasudil treatment or *ROCK1* knockdown, significantly affects acto-myosin contractility.

(a) High content imaging of mIMCD3 cells treated with siScr, si*Rpgrip1l* or si*Ift88* and either DMSO (left panel) or 10 µM fasudil (right panel) to show levels of changes non-muscle myosin IIA organization. Acto-myosin structures were visualized by MLCII staining and the presence of MLCII-associated acto-myosin structures. Antibodies marked MLCII, p-MLCII (mono-phosphorylated MLCII) and pp-MLCII (biphosphorylated MLCII at Thr18 & Ser19), with representative immunofluorescence confocal microscopy images shown. Automated high content image analysis of image texture quantified MLCII, p-MLCII and pp-MLCII fibre-like structures, with bar graphs showing decreases in texture for pp-MLCII that were not significant when cells were treated with 10 µM fasudil for 48 hrs. Statistical significance was calculated with a two way, unpaired Student's t-test (n.s. p=0.05459, t=3.7957, n=3 experimental replicates, each with n=4 technical replicates, 3 knockdown conditions df=16). Scale bar = 20 µm. **(b)** siRNA knockdown of *ROCK2* inhibits the kinase activity of ROCK2 changes non-muscle myosin IIA organization, visualized by MLCII staining and the presence of MLCII-associated acto-myosin structures in both mIMCD3 (left panels) and hTERT RPE-1 (right panels) cell-lines. Cells were treated with si*Rock1* / si*ROCK1*, si*Rock2* / si*ROCK2* or scrambled siRNA (siScr). Antibodies marked MLCII, p-MLCII (mono-phosphorylated MLCII) and pp-MLCII (biphosphorylated MLCII at Thr18 & Ser19), with representative immunofluorescence confocal microscopy images shown. p-MLCII and pp-MLCII visualize acto-myosin fibre-like structures in both mIMCD3 and hTERT RPE-1 cells indicated by arrowheads and displayed in magnified insets. Automated high content image analysis of image texture quantified both p-MLCII and pp-MLCII fibre-like structures, with bar graphs showing significant decreases for pp-MLCII staining following si*Rock2* / si*ROCK2* knockdowns only. Statistical significance was calculated with a two-way ANOVA with Dunnett's multiple comparisons test as indicated (mIMCD3: MLCII: siScr vs si*Rock1* n.s. p=0.2415, siScr vs si*Rock2*: n.s. *p*=0.6556; p-MLCII: siScr vs si*Rock1*: * *p*=0.0272, siScr vs si*Rock2*: ** *p*=0.0093; pp-MLCII: siScr vs si*Rock1*: n.s. *p*=0.2588, siScr vs si*Rock2*: * *p*=0.0167, all n=3 biological

replicates, F=9.1, df=18. hTERT RPE-1: MLCII: siScr vs si*Rock1*: n.s. *p*=0.7057, siScr vs si*Rock2*: n.s. *p*=0.3877; p-MLCII: siScr vs si*Rock1*: n.s. *p*=0.3352, siScr vs si*Rock2*: * *p*=0.0485; pp-MLCII: siScr vs si*Rock1*: n.s. *p*=0.1040, siScr vs si*Rock2*: * *p*=0.0376, all n=3 biological replicates, F=6.552, df=18.

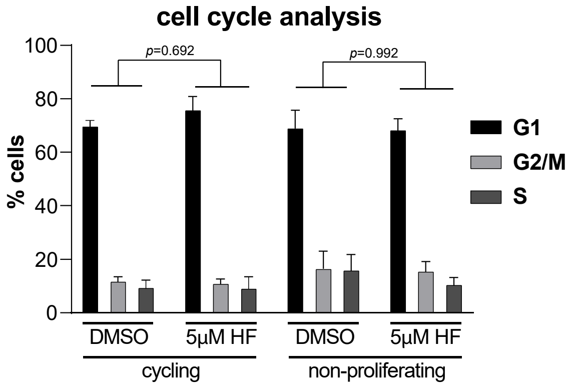

Supplemental Figure 4: Effects of hydroxyfasudil on the cell cycle.

Bar graph showing the percentage of hTERT RPE-1 cells in G_1, G_2/M and S phase of the cell cycle, determined using FACS of propidium iodide stained cells treated with hydroxyfasudil or vehicle (DMSO) control. Chi-squared test: DMSO vs 5 µM hydroxyfasudil in cycling cells: ns p=0.692, χ^2=2.237, df=4, n=3 biological replicates, 3 technical replicates. DMSO vs 5 µM hydroxyfasudil in non-proliferating cells: ns *p*=0.992, χ^2=0.265, df=4, n=3 biological replicates, 3 technical replicates.

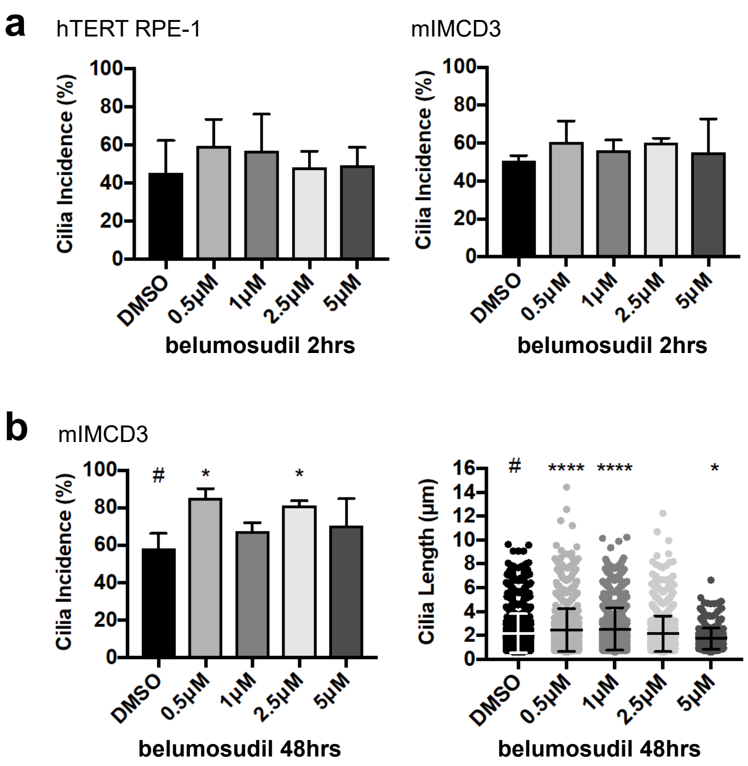

Supplemental Figure 5: Treatment with belumosudil for 48 hrs significantly increases cilia incidence and length.

(a) 2 hr treatment with belumosudil after 24 hrs serum starvation did not cause significant changes to either cilia incidence in hTERT RPE-1 (left panel) or mIMCD3 (right panel) cells. Data were confirmed to be normally distributed using D'Agostino-Pearson omnibus K2 test. Significance was then calculated using one-way ANOVAs with Dunnett's test for multiple corrections and was as follows: hTERT RPE-1: DMSO vs 0.5 μM belumosudil n.s. p=0.5684, DMSO vs 1 μM belumosudil n.s. p=0.7219, DMSO vs 2.5 μM belumosudil n.s. p=0.9973, DMSO vs 5 μM belumosudil n.s. p=0.9897, n=3 biological replicates, F=0.5540, df=10; mIMCD3: DMSO vs 0.5 μM belumosudil n.s. p=0.5360, DMSO vs 1 μM belumosudil n.s. p=0.8416, DMSO vs 2.5 μM belumosudil n.s. p=0.4848, DMSO vs 5 μM belumosudil n.s. p=0.9405, n=3 biological replicates, F=0.6633, df=8 Error bars represent S.D. **(b)** 48 hr treatment of with belumosudil in serum starvation conditions led to significant increases in cilia incidence (left panels) and length (right panels) in mIMCD3 cells compared to DMSO-treated control cells (n=3 experimental replicates, with at least 100 cilia measured per experimental replicate). Data for cilia incidence were confirmed to be normally distributed using D'Agostino-

Pearson omnibus K2 test, whereas cilia length data were not normally distributed. Significance was then calculated using one-way ANOVAs with Dunnett's test for multiple corrections for cilia incidence data as follows: DMSO 0.5 μM belumosudil * *p*=0.0174, DMSO vs 1 μM belumosudil n.s. *p*=0.6368, DMSO vs 2.5 μM belumosudil * *p*=0.0441, DMSO vs 5 μM belumosudil n.s. p=0.4036, n=3 biological replicates, F=4.186, df=12. Kruskal-Wallis tests with Dunn's multiple comparisons test were used for cilia length data as follows: DMSO 0.5 μM belumosudil **** *p*<0.0001, Z=4.539, DMSO vs 1 μM belumosudil **** *p*<0.0001, Z=5.731, DMSO vs 2.5 μM belumosudil n.s. *p*>0.9999, Z=0.7758, DMSO vs 5 μM belumosudil * *p*=0.0114, Z=3.052, n=3 biological replicates, KW stat=106.6. n=3 biological replicates with 1142 (DMSO), 979 (0.5 μM belumosudil), 831 (1 μM belumosudil), 883 (2.5 μM belumosudil) and 386 (5 μM belumosudil) cilia measured in total *=*p*<0.05, **=*p*<0.01, ***=*p*<0.001, ****=*p*< 0.0001. # is the control to which all data sets were compared. Error bars represent S.D. Equivalent data for hTERT RPE-1 cells is shown in Figure 6a.

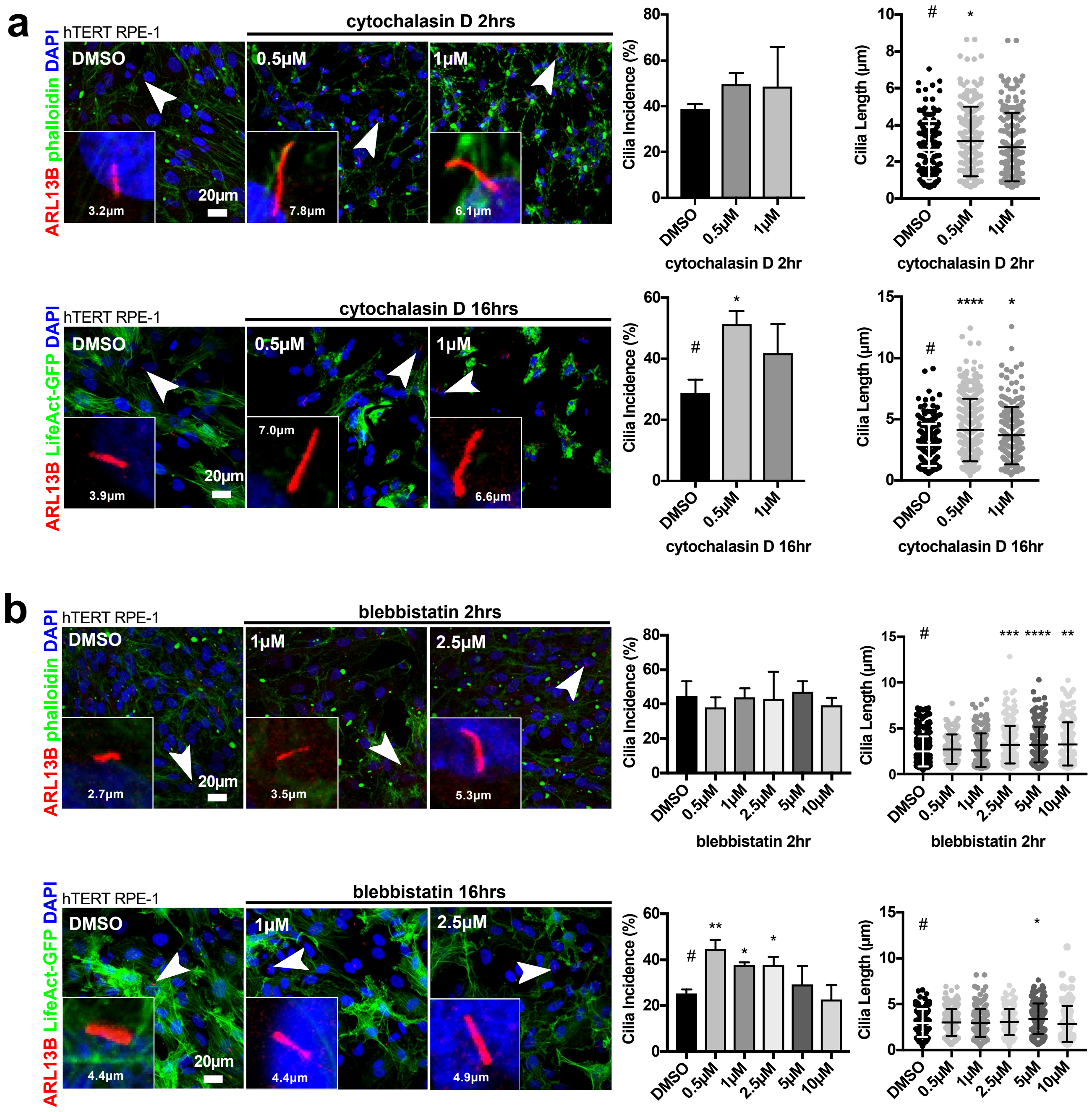

Supplemental Figure 6: Treatment with cytochalasin D and blebbistatin increases ciliogenesis.

(a) Pharmacological inhibition of F-actin stabilisation though treatment with cytochalasin D increased ciliogenesis. hTERT RPE-1 cells stably transfected with LifeAct-GFP (green) were treated for either 2 hrs (upper panels) with cytochalasin D or vehicle control after 24 hours of serum starvation, or for 16 hrs (lower panels) with cytochalasin D or vehicle control in serum

starvation media (all conditions n=3 biological replicates). Representative confocal microscopy images show cells stained with cilia marker ARL13B (red) with arrowheads indicating cilia displayed in magnified insets and their measured lengths. Bar graphs quantitate the effect of inhibition with cytochalasin D at the indicated concentrations on cilia incidence and length. Cells treated with cytochalasin D for 2 hrs had a slight but non-significant (n.s.) increase in cilia incidence and increase in mean length (significant at 0.5 μM). Cells treated for 16 hrs had significant increases in cilia incidence and mean cilia length. A minimum of 20 cilia were measured per replicate condition. Statistical significance was calculated with a one-way ANOVA with a Dunnett's multiple comparisons test for cilia incidence data: 2hrs: DMSO vs 0.5 μM cytochalasin D: n.s. p=0.3856, DMSO vs 1 μM cytochalasin D: n.s. p=0.4518 n=3, F=0.4189, df=6; 16hrs: DMSO vs 0.5 μM cytochalasin D: * p=0.0101, DMSO vs 1 μM cytochalasin D: n.s. p=0.0904 n=3, F=8.914, df=6. A Kruskal-Wallis test with Dunn's multiple comparisons test was used to assess cilia length data: 2hrs: DMSO vs 0.5 μM cytochalasin D: n.s. p=0.0995, Z=1.962, DMSO vs 1 μM cytochalasin D: n.s. p>0.9999, Z=0.3217, n=3 biological replicates with 146 (DMSO), 200 (0.5 μM cytochalasin D), 179 (1 μM cytochalasin D) cilia measured in total, KW statistic 4.755; 16hrs: DMSO vs 0.5 μM cytochalasin D: **** p<0.0001, Z=4.888, DMSO vs 1 μM cytochalasin D: * p=0.0152, Z=2.669, n=3 biological replicates with 178 (DMSO), 385 (0.5 μM cytochalasin D), 203 (1 μM cytochalasin D) cilia measured in total, KW statistic=24.02 (* p<0.05, **=p<0.01, ***=p<0.001, ****=p<0.0001). # indicates the control to which all data-sets were compared. **(b)** Pharmacological inhibition of non-muscle myosin II ATPase activity through treatment with blebbistatin increased ciliogenesis. hTERT RPE-1 cells stably transfected with LifeAct-GFP were treated for either 2 hrs (upper panels) with blebbistatin or vehicle control after 24 hrs of serum starvation, or for 16 hrs (lower panels) with blebbistatin or vehicle control in serum starvation media (all conditions n=3). Cells treated for 2 hrs had no significant changes in cilia incidence across all blebbistatin concentrations, although there was a significant increase in mean cilia length for concentrations ≥ 2.5 μM. A minimum of 25 cilia were measured per replicate condition. Statistical significance was calculated as for (a). Values were: cilia incidence data: 2hrs: DMSO vs 0.5 μM blebbistatin: n.s. p=0.8158, DMSO vs 1 μM blebbistatin: n.s. p>0.9999,

DMSO vs 2.5 μM blebbistatin: n.s. *p*=0.9994, DMSO vs 5 μM blebbistatin: n.s. *p*=0.9978, DMSO vs 10 μM blebbistatin: n.s. *p*=0.9312, n=3, F=0.4209, df=11; 16hrs: DMSO vs 0.5 μM blebbistatin: ** *p*=0.0042, DMSO vs 1 μM blebbistatin: * *p*=0.0359 DMSO vs 2.5 μM blebbistatin: * *p*=0.0328, DMSO vs 5 μM blebbistatin: n.s. *p*=0.7616, DMSO vs 10 μM blebbistatin: n.s. *p*=0.9396 n=3 biological replicates (apart from 0.5 μM blebbistatin, since images were found to be unusable for automatic recognition for one replicate), F=8.121, DF=11. Cilia length: 2hrs: DMSO vs 0.5 μM blebbistatin: n.s. *p*=0.5954, Z=1.559, DMSO vs 1 μM blebbistatin: n.s. *p*>0.9999, Z=0.2323, DMSO vs 2.5 μM blebbistatin: *** *p*=0.0002, Z=4.103, DMSO vs 5 μM blebbistatin: **** *p*<0.0001, Z=4.297, DMSO vs 10 μM blebbistatin: ** *p*=0.0051. Z=3.285, n=3 biological replicates with 325 (DMSO), 140 (0.5 μM blebbistatin), 218 (1 μM blebbistatin), 266 (2.5 μM blebbistatin), 231 (5 μM blebbistatin) and 193 (10 μM blebbistatin) cilia measured in total, KW stat=33.82. 16hrs: DMSO vs 0.5 μM blebbistatin: n.s. *p*>0.9999, Z=0.4299, DMSO vs 1 μM blebbistatin: ns *p*>0.9999, Z=0.1481, DMSO vs 2.5 μM blebbistatin: n.s. *p*>0.9999, Z=0.7216, DMSO vs 5 μM blebbistatin: * *p*=0.0691, Z=2.462, DMSO vs 10 μM blebbistatin: n.s. *p*>0.9999, Z=1.180, n=3 biological replicates with 160 (DMSO), 234 (0.5 μM blebbistatin), 271 (1 μM blebbistatin), 232 (2.5 μM blebbistatin), 170 (5 μM blebbistatin) and 118 (10 μM blebbistatin) cilia measured in total, KW stat=14.36.

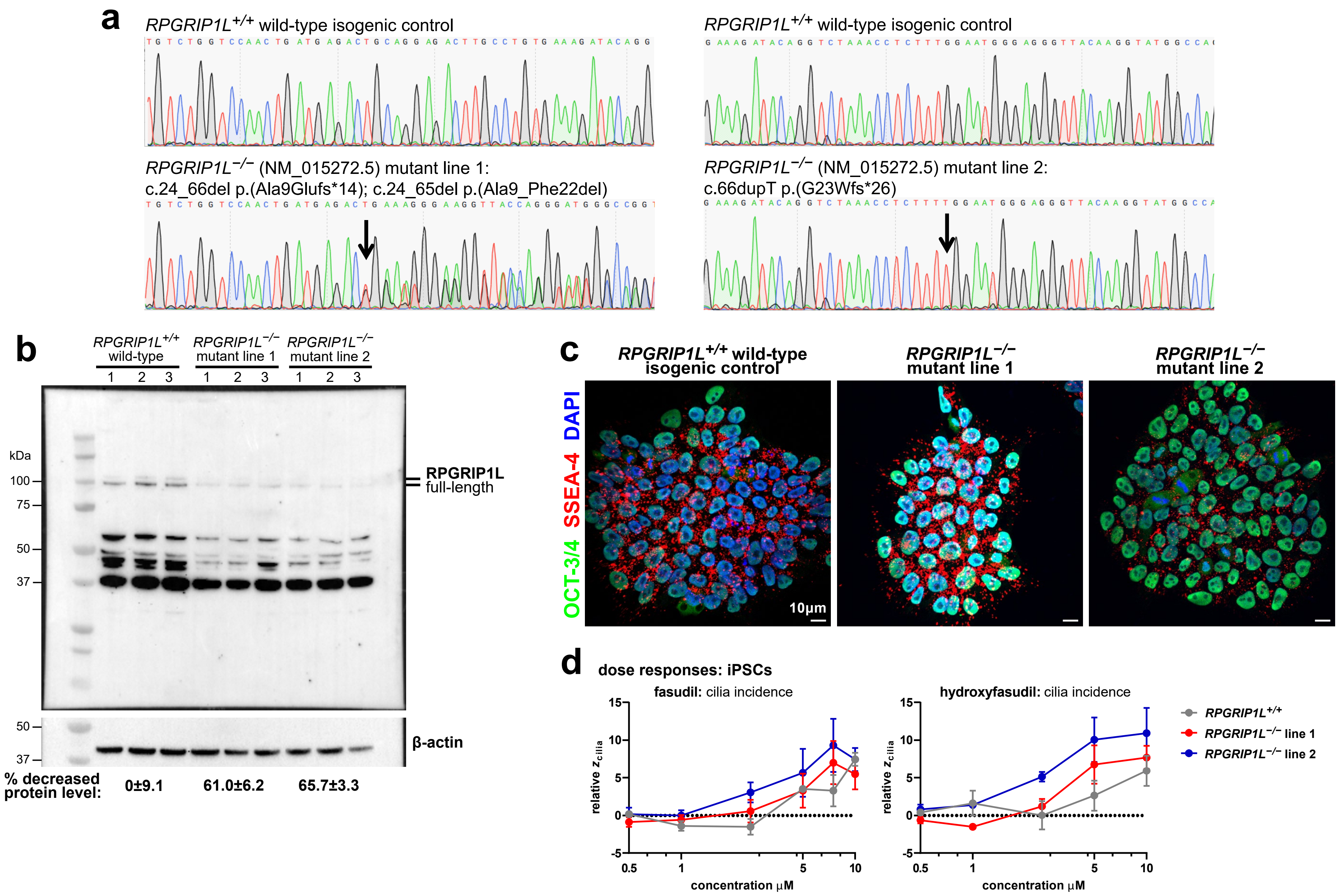

a
RPGRIP1L+/+ wild-type isogenic control
RPGRIP1L+/+ wild-type isogenic control
RPGRIP1L−/− (NM_015272.5) mutant line 1:
c.24_66del p.(Ala9Glufs*14); c.24_65del p.(Ala9_Phe22del)
RPGRIP1L−/− (NM_015272.5) mutant line 2:
c.66dupT p.(G23Wfs*26)
b
RPGRIP1L+/+ wild-type
RPGRIP1L−/− mutant line 1
RPGRIP1L−/− mutant line 2
1 2 3 1 2 3 1 2 3
kDa
100
75
50
37
RPGRIP1L full-length
50
37
β-actin
% decreased protein level:
0±9.1
61.0±6.2
65.7±3.3
c
RPGRIP1L+/+ wild-type isogenic control
RPGRIP1L−/− mutant line 1
RPGRIP1L−/− mutant line 2
OCT-3/4 SSEA-4 DAPI
10µm
d
dose responses: iPSCs
fasudil: cilia incidence
hydroxyfasudil: cilia incidence
relative zcilia
15
10
5
0
-5
0.5
1
5
10
concentration µM
RPGRIP1L+/+
RPGRIP1L−/− line 1
RPGRIP1L−/− line 2

Supplemental Figure 7: Characterization of isogenic control and CRISPR-Cas9 engineered *RPGRIP1L* mutant hiPSC cell lines.

(a) Sanger sequencing electropherograms showing wild-type *RPGRIP1L*$^{+/+}$ isogenic control line sequencing (upper panels) in comparison to *RPGRIP1L*$^{-/-}$ (NM_015272.5) mutant line 1 (left): c.24_66del p.(Ala9Glufs*14); c.24_65del p.(Ala9_Phe22del) and mutant line 2 (right): sequencing (lower panels). Arrows indicate the position of the first altered base. **(b)** Western blot of protein from wild-type *RPGRIP1L*$^{+/+}$ isogenic control cells, mutant line 1 and mutant line 2. 30 mg total protein was loaded. Full length RPGRIP1L is seen at around 100 kDa in two isoforms. Smaller isoforms are seen between 37 kDa and around 55 kDa. Mutant lines show a visible reduction in both the full-length isoforms and the smaller isoforms. The effect on the two largest isoforms was quantified by densitometry with comparison to β actin loading control: n=3 biological replicates, error is SEM. An unpaired, two-tailed Student's t test showed the there was a significant decrease in RPGRIP1L expression for both *RPGRIP1L*$^{-/-}$ mutant lines: mutant line 1: * p=0.02208, t=3.9180 and mutant line 2: ** p=0.006830, t=4.1669, n=3, df=4 **(c)** Immunofluorescence confocal imaging of pluripotency markers OCT3/4 (green), SSEA-4 (red) and DAPI (nuclei; blue) of wild-type *RPGRIP1L*$^{+/+}$ isogenic control cells (left), *RPGRIP1L*$^{-/-}$ mutant lines 1 and 2. Scale bar = 10μm. **(d)** Dose response assays showing z_{cilia} relative to that of wild-type untreated cells for hiPSC CRISPR-Cas9 engineered cell lines. Significant increases in z_{cilia} were apparent from doses of around 5 μM for fasudil and around 2.5 μM for hydroxyfasudil. n=3 biological replicates. Error bars indicate the SEM.

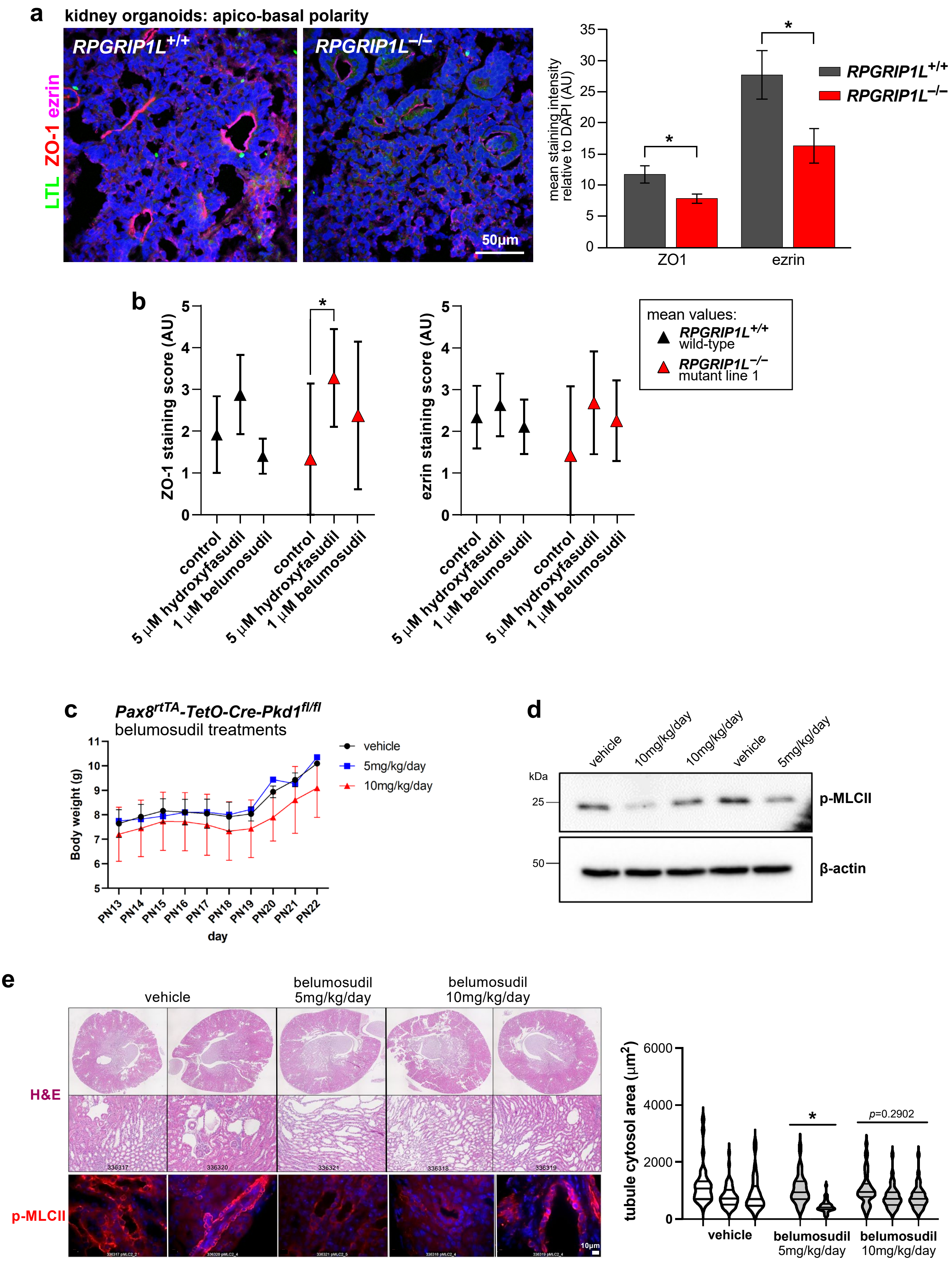
a
kidney organoids: apico-basal polarity
RPGRIP1L+/+
RPGRIP1L−/−
LTL ZO-1 ezrin
50µm
mean staining intensity relative to DAPI (AU)
35
30
25
20
15
10
5
0
ZO1
ezrin
RPGRIP1L+/+
RPGRIP1L−/−
b
ZO-1 staining score (AU)
ezrin staining score (AU)
5
4
3
2
1
0
control
5 µM hydroxyfasudil
1 µM belumosudil
mean values:
RPGRIP1L+/+
wild-type
RPGRIP1L−/−
mutant line 1
c
Pax8rtTA-TetO-Cre-Pkd1fl/fl
belumosudil treatments
Body weight (g)
11
10
9
8
7
6
5
PN13
PN14
PN15
PN16
PN17
PN18
PN19
PN20
PN21
PN22
day
vehicle
5mg/kg/day
10mg/kg/day
d
vehicle
10mg/kg/day
10mg/kg/day
vehicle
5mg/kg/day
kDa
25
50
p-MLCII
β-actin
e
vehicle
belumosudil
5mg/kg/day
belumosudil
10mg/kg/day
H&E
p-MLCII
10µm
tubule cytosol area (µm2)
6000
4000
2000
0
p=0.2902
vehicle
belumosudil
5mg/kg/day
belumosudil
10mg/kg/day

Supplemental Figure 8: Apico-basal polarity defects in *RPGRIP1L*$^{-/-}$ kidney organoids and functional effects on MLCII phosphorylation in the conditional cystic mouse model *Pax8*^{rtTA}*-TetO-Cre-Pkd1*$^{fl/fl}$ of PKD

(a) Immunofluorescence confocal imaging of isogenic wild-type and *RPGRIP1L*$^{-/-}$ mutant line 1 organoids at day 23 of differentiation to show differences in apico-basal polarity (left). Organoid sections were stained with LTL (green; proximal tubules), ZO-1 (red; tight junctions), ezrin (magenta; apical cell surface) and DAPI (blue; nuclei). Scale bar = 50 μm. Bar graph (right) shows the quantification of ZO-1 and ezrin staining relative to DAPI for both cell lines. Both ZO1 and ezrin staining are significantly less for mutant line 1 compared to wildtype: Unpaired, two-tailed Student's t test: ZO-1 intensity relative to DAPI: wild-type vs *RPGRIP1L*$^{-/-}$ mutant line 1: * 0.0127, t=5.1341 n=3 biological replicates each with 2-7 technical replicates, df=20. Ezrin intensity relative to DAPI: wild-type vs *RPGRIP1L*$^{-/-}$ mutant line 1: * 0.0291, t=4.5782 n=3 biological replicates each with 2-7 technical replicates, df=20. **(b)** Graphs to show the mean staining score for ZO-1 (left) and ezrin (right) for wild-type and *RPGRIP1L*$^{-/-}$ mutant line 1 day 23 organoid sections for organoids treated with ROCK inhibitors or 0.1% DMSO vehicle control. Error bars show SD. Student's t test: *RPGRIP1L*$^{-/-}$ mutant: vehicle control vs 5 μM hydroxyfasudil ZO-1 staining score: * p=0.0164, t=4.7885 n=3 biological replicates, each with 2-4 technical replicates. **(c)** Belumosudil-treated *Pkd1* mice (5 and 10 mg/kg/day doses by i.p. injections from PN16; n = 5) showed similar weight gain to vehicle-injected control animals before (n = 5) and after treatment for 7 days (PN22). Total animals assessed n=15. **(d)** Kidney tissue from belumosudil-treated animals had reduced levels of p-MLCII relative to vehicle control-treated animals. Loading control is β-actin. **(e)** Left: Kidney section stained with H&E show extensive renal cystic changes and variably reduced levels of p-MLCII (red) following belumosudil treatments. Scale bar=10μm. Right: Violin plot of individual renal tubule area per kidney for drug treatments (n=2 or 3 animals, as indicated, with n=121, 83 and 122 tubules respectively), showing a significant decrease in tubule cytosol areas for 5 mg/kg/day but not for 10 mg/kg/day (unpaired two-tailed Student's t tests for tubules from vehicle vs 5 mg/kg/day belumosudil animal groups, * p=0.0330. t=4.3317, df=202, vehicle vs 10 mg/kg/day belumosudil animal groups, ns p=0.02902 t=2.1193, df=241).

Supplemental Tables

Compound	IC_{50} ROCK1 (µM)	IC_{50} ROCK2 (µM)	Details	Source
Fasudil	0.3	"similar" to 0.3	1 µM ATP, human ROCK1 (aa 3-543) expressed in a baculovirus system [1]	Doe, et al. [2]
	N/A	1.9	100 µM ATP, human ROCK2, expressed in a baculovirus system	Davies, et al. [3]
	1.2	0.82	No details	Rikitake, et al. [4]
	10.7 ± 2		250 µM ATP, bovine brain derived ROCK[A]	Swärd, et al. [5]
	2.670 ± 0.270	1.290 ± 0.080	40 µM ATP, human ROCK1 and ROCK2[B]	Löhn, et al. [6]
	0.844 (1.174-0.515)	0.691 (0.893-0.489)	10 µM ATP, human ROCK1 (aa 1-535), human ROCK2 (aa 1-552)	Cantoni, et al. [7]
	N/A	0.158	No details	Tamura, et al. [8C]
Hydroxyfasudil	0.73	0.72	No details	Rikitake, et al. [4]
Ripasudil	0.051	0.019	1 µM ATP, human ROCK1 (aa 1-477), ROCK2 (aa 1-553) expressed in a baculovirus system	Isobe, et al. [9]
Belumosudil (KD-025)	24	0.105	10 µM ATP, human ROCK1 (aa 1-535), human ROCK2 (aa 1-552) expressed in a baculovirus system	Boerma, et al. [10]
	>10	0.059	10 µM ATP, human ROCK1 (aa 1-535), human ROCK2 (aa 1-552) expressed in a baculovirus system	Lee, et al. [11]
	5.1	0.07	No details	Keystone Symposia on Molecular and Cellular Biology[D]
	9.8	0.16	No details	Paris NASH meeting[E]

Supplemental Table 1: IC_{50} values for the ROCK inhibitors used in this study. Note that the ATP concentration in cells is in the mM range, which is higher than that used for in vitro assays. However, in vivo activity is likely to be significantly lower than in vitro activity due to competition for ATP. Cell based assay IC_{50} values are often 100-1000 fold higher than in vitro equivalents [12]. Alternative names: fasudil (hydrochloride) / HA1077, hydroxyfasudil / HA1100, ripasudil / K115, belumosudil / KD025 / SLx-2119 / RezuROCK

[A] It is unclear whether unpurified ROCK, ROCK1 or ROCK2 was assayed, but many manufacturers specify this IC_{50} value for ROCK1.

[B] The authors highlight differences in ATP K_m values for ROCK1 and ROCK2, concluding that fasudil is not selective for either isoform.

[C] This reference is a review article with no experimental reference specified for the information stated.
[D]Keystone symposia on Molecular and Cellular Biology, January 2019: https://www.redxpharma.com/app/uploads/2019/01/ROCK2-poster-NASH-Keystone_Jan-2019_Emily-Offer.pdf
[E]Paris NASH meeting, July 2019: https://www.redxpharma.com/app/uploads/2019/07/ROCK2-poster-NASH-July-2019_Emily-Offer.pdf

**Compound**	**K_i ROCK1 µM**	**K_i ROCK2 µM**	**K_i ROCK µM**	**Details**	**Source**
Fasudil			0.35	100 µM ATP, bovine brain derived ROCK	Ito, et al. [13]
	0.145 ± 0.007	0.112 ± 0.008		10 µM ATP, human ROCK1 (aa 1-535), human ROCK2 (aa 1-552) expressed in a baculovirus system	Lee, et al. [11]
			0.4	100 µM ATP, kinase domain of ROCK (aa 6-553)	Amano, et al. [14]
		0.271 ± 0.014		40 µM ATP, human ROCK2	Löhn, et al. [6]
Hydroxyfasudil			0.56	No details	Ito, et al. [13]
Belumosudil (KD-025)	>10	0.041 ± 0.002		10 µM ATP, human ROCK1 (aa 1-535), human ROCK2 (aa 1-552)	Lee, et al. [11]

Supplemental Table 2: K_i values for the ROCK inhibitors included in this study. Note that K_i values for ripasudil are unavailable [15].
Alternative names: fasudil (hydrochloride) / HA1077, hydroxyfasudil / HA1100, ripasudil / K115, KD025 / SLx-2119 / belumosudil.
[A] Ito et al. [13] cites Seto et al. [16] but there is no information present for hydroxyfasudil

References for Supplemental Tables

1 Khandekar, S. S. *et al.* Expression, purification, and characterization of an enzymatically active truncated human rho-kinase I (ROCK I) domain expressed in Sf-9 insect cells. *Protein Pept Lett* **13**, 369-376 (2006). https://doi.org/10.2174/092986606775974357

2 Doe, C. *et al.* Novel Rho kinase inhibitors with anti-inflammatory and vasodilatory activities. *J Pharmacol Exp Ther* **320**, 89-98 (2007). https://doi.org/10.1124/jpet.106.110635

3 Davies, S. P., Reddy, H., Caivano, M. & Cohen, P. Specificity and mechanism of action of some commonly used protein kinase inhibitors. *Biochem J* **351**, 95-105 (2000). https://doi.org/10.1042/0264-6021:3510095

4 Rikitake, Y. *et al.* Inhibition of Rho kinase (ROCK) leads to increased cerebral blood flow and stroke protection. *Stroke* **36**, 2251-2257 (2005). https://doi.org/10.1161/01.Str.0000181077.84981.11

5 Swärd, K. *et al.* Inhibition of Rho-associated kinase blocks agonist-induced Ca2+ sensitization of myosin phosphorylation and force in guinea-pig ileum. *J Physiol* **522 Pt 1**, 33-49 (2000). https://doi.org/10.1111/j.1469-7793.2000.0033m.x

6 Löhn, M. *et al.* Pharmacological characterization of SAR407899, a novel rho-kinase inhibitor. *Hypertension* **54**, 676-683 (2009). https://doi.org/10.1161/hypertensionaha.109.134353

7 Cantoni, S. *et al.* Pharmacological characterization of a highly selective Rho kinase (ROCK) inhibitor and its therapeutic effects in experimental pulmonary hypertension. *Eur J Pharmacol* **850**, 126-134 (2019). https://doi.org/10.1016/j.ejphar.2019.02.009

8 Tamura, M. *et al.* Development of specific Rho-kinase inhibitors and their clinical application. *Biochim Biophys Acta* **1754**, 245-252 (2005). https://doi.org/10.1016/j.bbapap.2005.06.015

9 Isobe, T. *et al.* Effects of K-115, a rho-kinase inhibitor, on aqueous humor dynamics in rabbits. *Curr Eye Res* **39**, 813-822 (2014). https://doi.org/10.3109/02713683.2013.874444

10 Boerma, M. *et al.* Comparative gene expression profiling in three primary human cell lines after treatment with a novel inhibitor of Rho kinase or atorvastatin. *Blood Coagul Fibrinolysis* **19**, 709-718 (2008). https://doi.org/10.1097/MBC.0b013e32830b2891

11 Lee, J. H. *et al.* Selective ROCK2 Inhibition In Focal Cerebral Ischemia. *Ann Clin Transl Neurol* **1**, 2-14 (2014). https://doi.org/10.1002/acn3.19

12 Mueller, B. K., Mack, H. & Teusch, N. Rho kinase, a promising drug target for neurological disorders. *Nat Rev Drug Discov* **4**, 387-398 (2005). https://doi.org/10.1038/nrd1719

13 Ito, K. *et al.* Essential role of rho kinase in the Ca2+ sensitization of prostaglandin F(2alpha)-induced contraction of rabbit aortae. *J Physiol* **546**, 823-836 (2003). https://doi.org/10.1113/jphysiol.2002.030775

14 Amano, M. *et al.* The COOH terminus of Rho-kinase negatively regulates rho-kinase activity. *J Biol Chem* **274**, 32418-32424 (1999). https://doi.org/10.1074/jbc.274.45.32418

15 Koch, J. C. *et al.* ROCK inhibition in models of neurodegeneration and its potential for clinical translation. *Pharmacol Ther* **189**, 1-21 (2018). https://doi.org/10.1016/j.pharmthera.2018.03.008

16 Seto, M., Sasaki, Y., Hidaka, H. & Sasaki, Y. Effects of HA1077, a protein kinase inhibitor, on myosin phosphorylation and tension in smooth muscle. *Eur J Pharmacol* **195**, 267-272 (1991). https://doi.org/10.1016/0014-2999(91)90545-2
