# Supplementary material for: Drug and siRNA screens identify ROCK2 as a therapeutic target for ciliopathies
Source: Commun Med (Lond). 2025 Apr 19;5:129. doi: 10.1038/s43856-025-00847-1 (PMC12009310; doi:10.1038/s43856-025-00847-1)
Supplement: Supplementary file 2 — Description of Additional Supplementary File [file 43856_2025_847_MOESM2_ESM.pdf]

Description Of Additional Supplementary File

File name: Supplemental Data 1
Description: Results from high content imaging screen to identify drugs that restore cilia after siRNA knockdown. Primary screen: used the Tocriscreen Total library (1120 biologically active clinical development compounds) in mIMCD3 cells treated with si<i>Rpgrip1l</i>.Secondary screen: used 71 chemicals in mIMCD3 cells treated with si<i>Rpgrip1l</i>. Tertiary screen: used 25 chemicals in hTERT RPE-1 cells treated with si<i>RPGRIP1L</i> and si<i>IFT88</i>.

File name: Supplemental Data 2
Description: High content imaging screen to identify siRNAs that increased cilia incidence. Primary screen results are available in full in supplementary table 1 in Wheway et al. 2015: https://doi.org/10.1038/ncb3201 Secondary screen: used 83 siRNAs in mIMCD3 cells. Additional information is available at https://etheses.whiterose.ac.uk/27888/

File name: Supplemental Data 3
Description: Macro for identifying and measuring cilia.

File name: Supplemental Data 4
Description: All data used for Figures or Supplemental Figures

File name: Supplemental Data 5
Description: All unedited blots used for Figures or Supplemental Figures

File name: Supplemental Data 6
Description: Sanger sequencing datafiles (.ab1 files) for RPGRIP1L mutant iPSC lines
