# Supplementary material for: Drug and siRNA screens identify ROCK2 as a therapeutic target for ciliopathies
Source: Commun Med (Lond). 2025 Apr 19;5:129. doi: 10.1038/s43856-025-00847-1 (PMC12009310; doi:10.1038/s43856-025-00847-1)
Supplement: Supplementary file 7 — Supplemental Data 5 [file 43856_2025_847_MOESM7_ESM.pdf]

Other supporting data are available at:

https://etheses.whiterose.ac.uk/27888/
https://www.nature.com/articles/ncb3201
https://onlinelibrary.wiley.com/doi/10.1111/ahg.12529

Protein marker used throughout is Precision Plus Protein Standard

Full unedited blot for Figure 4e

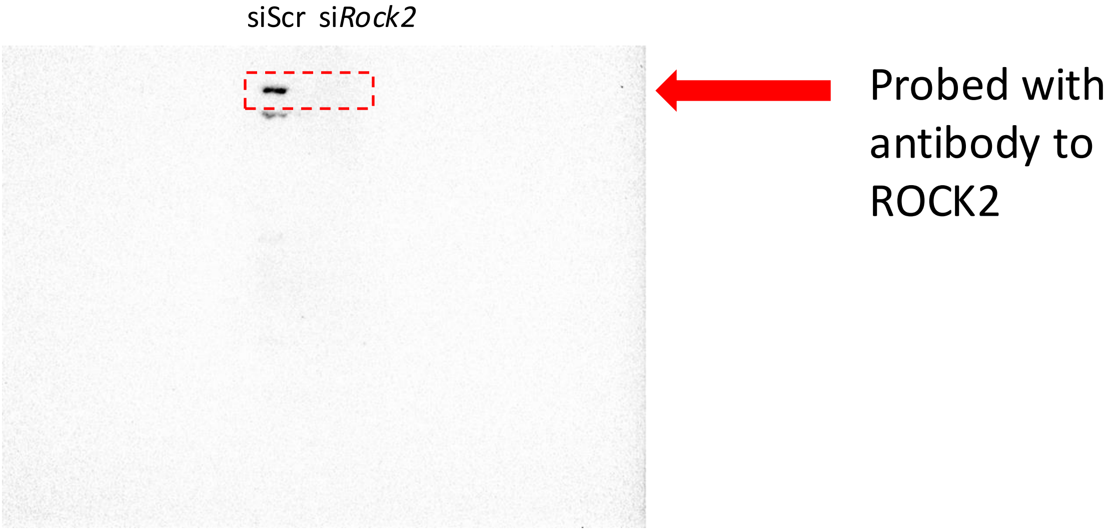

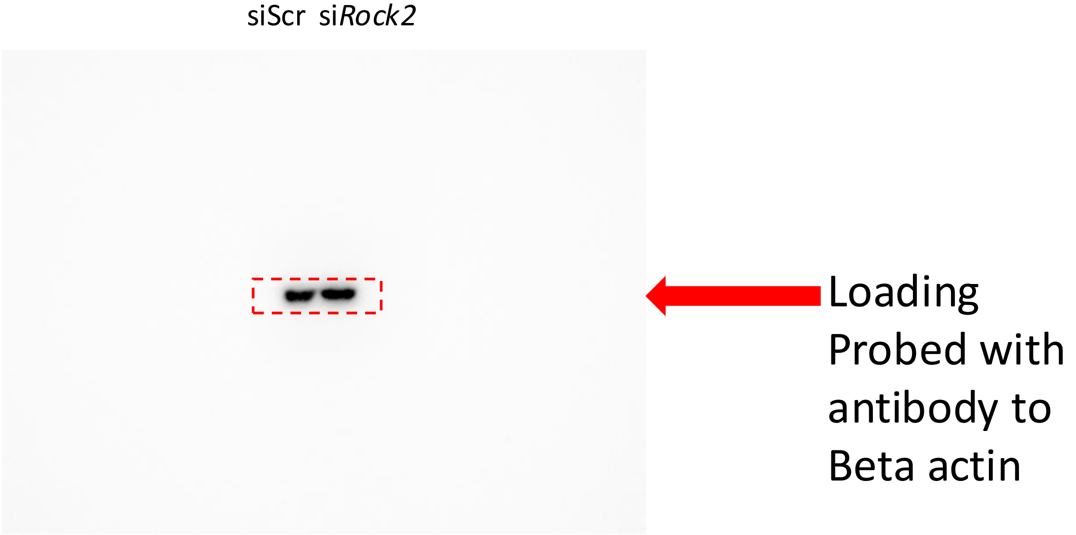

Full unedited blot for Supplemental Figure 2a

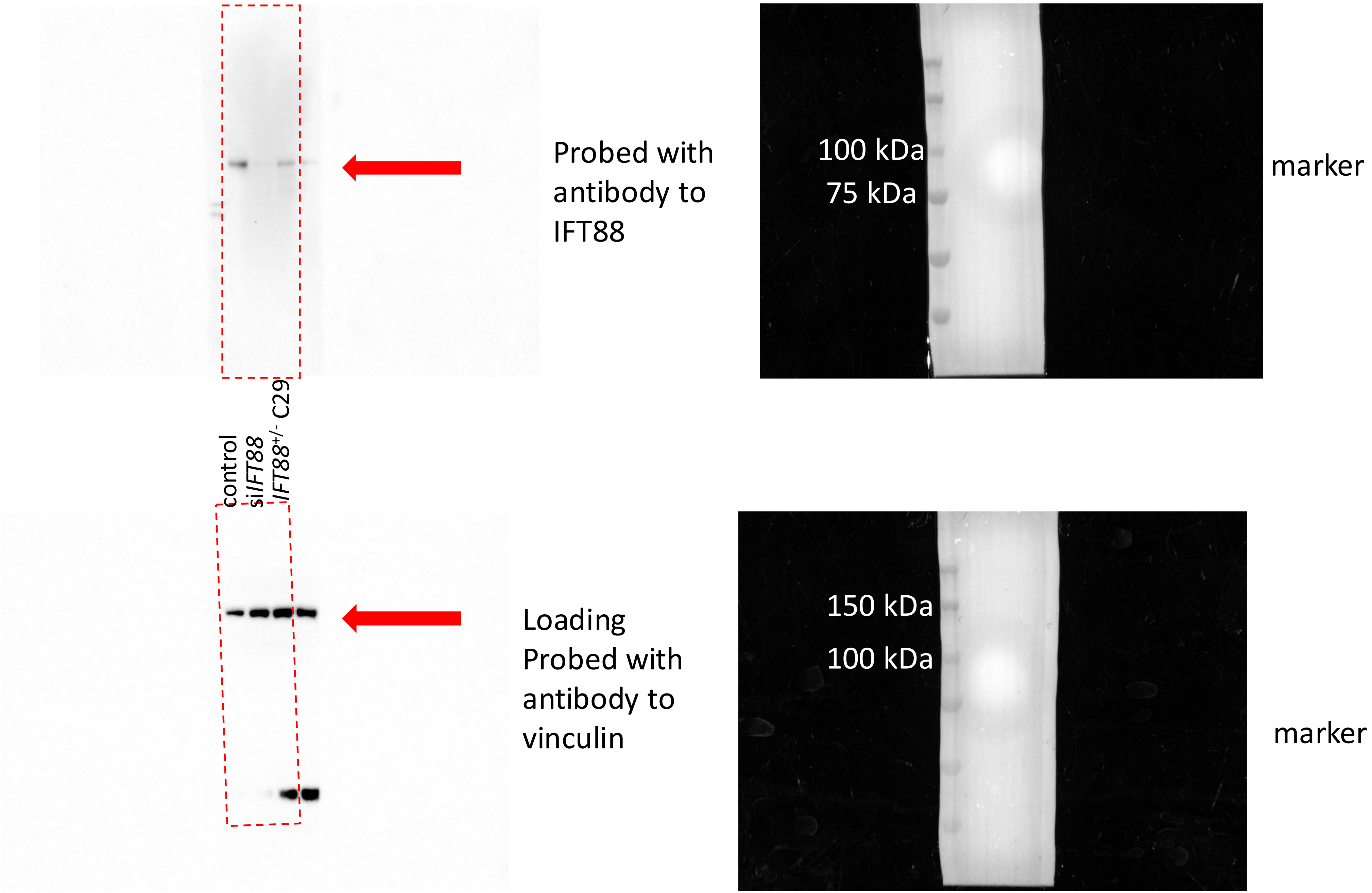

Full unedited blot for Supplemental Figure 2b

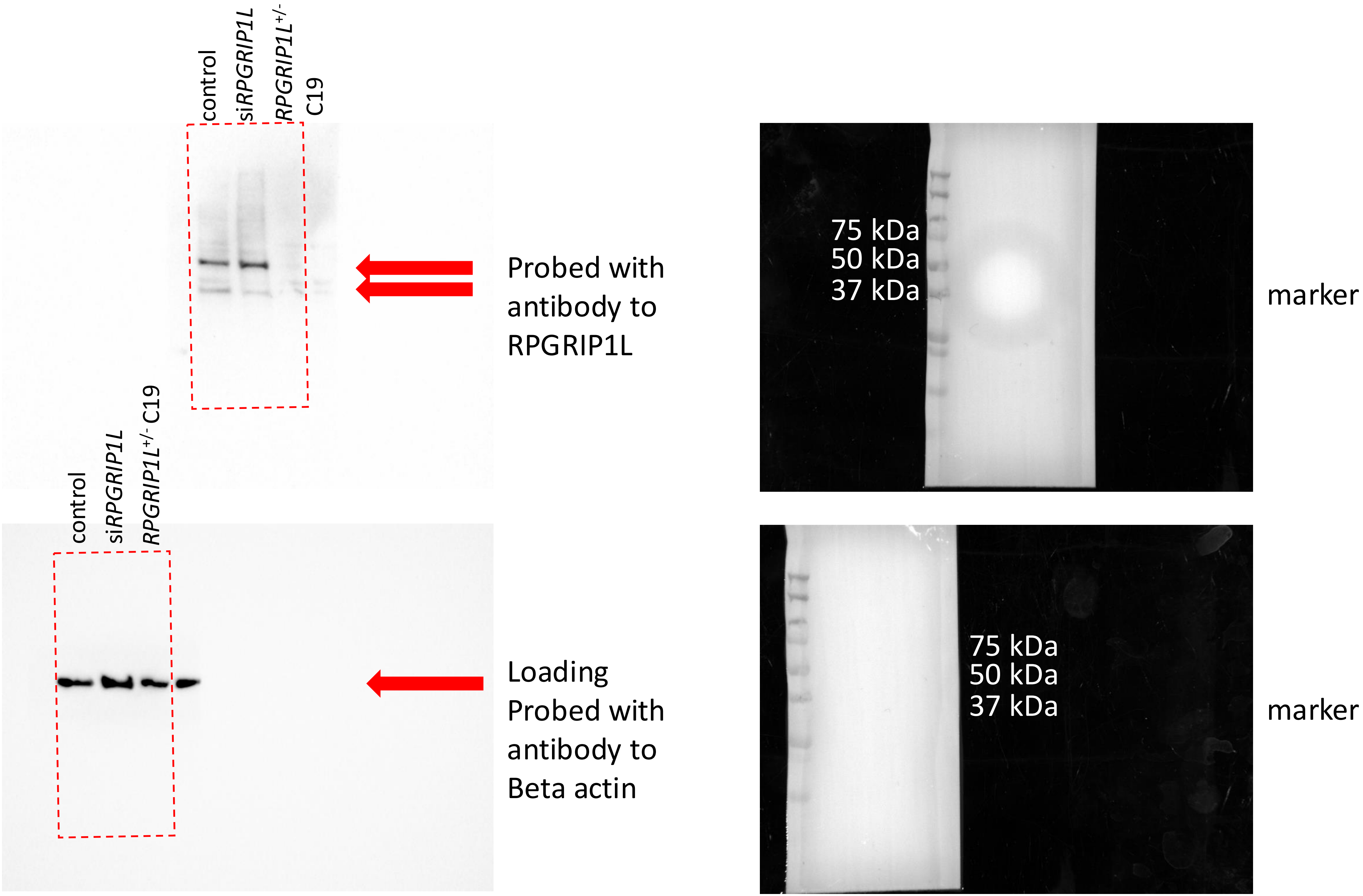

Full unedited blot for Supplemental Figure 2c

Raw images (Merged with ladder)

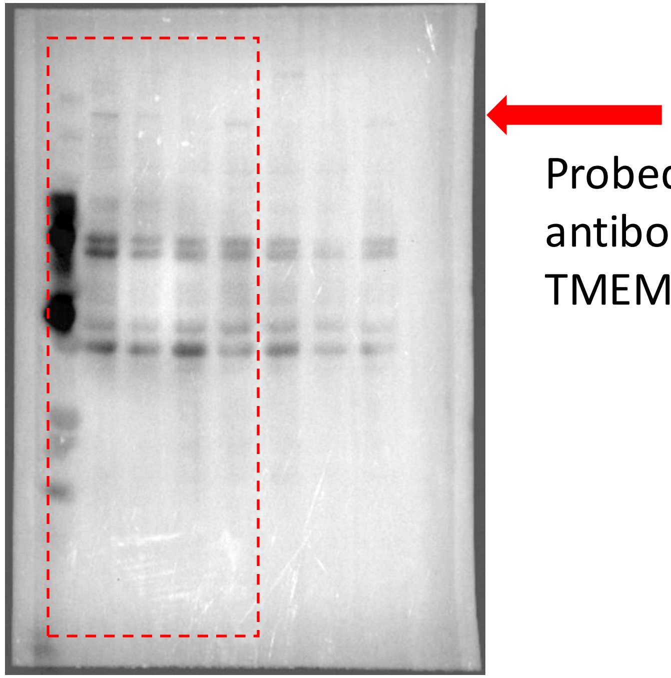

Probed with antibody to TMEM67

Raw images (Merged with ladder)

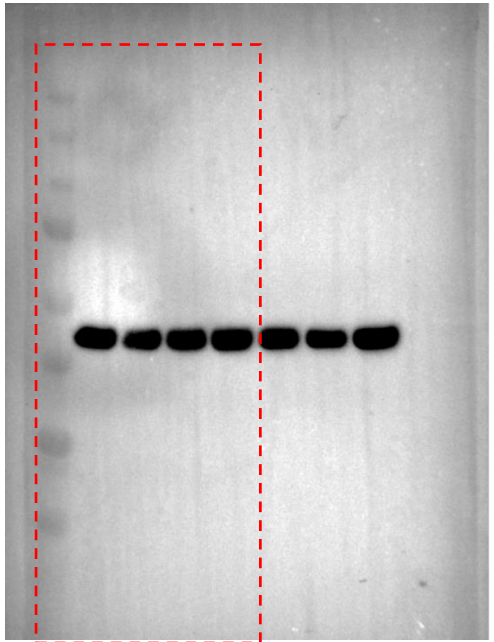

Loading Probed with antibody to Beta actin

Zoom of region (labelled)

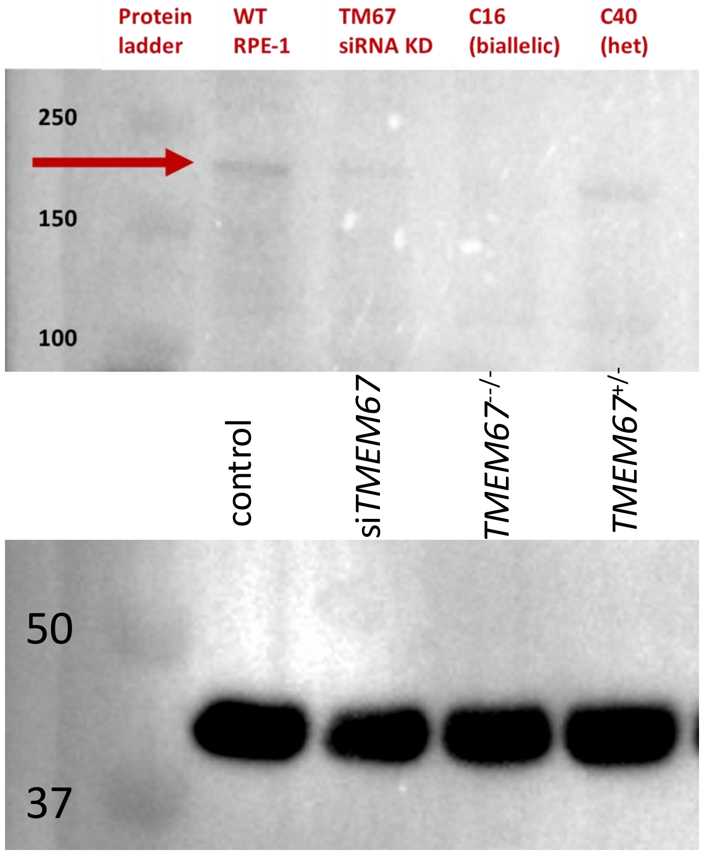

Full unedited blot for Supplemental Figure 6b

Raw images: not merged

Raw images: combined with marker

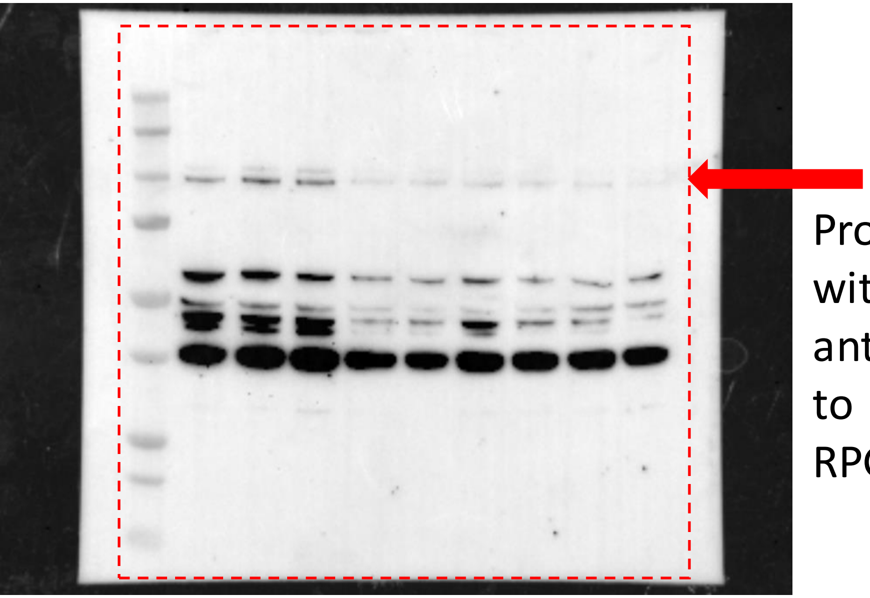

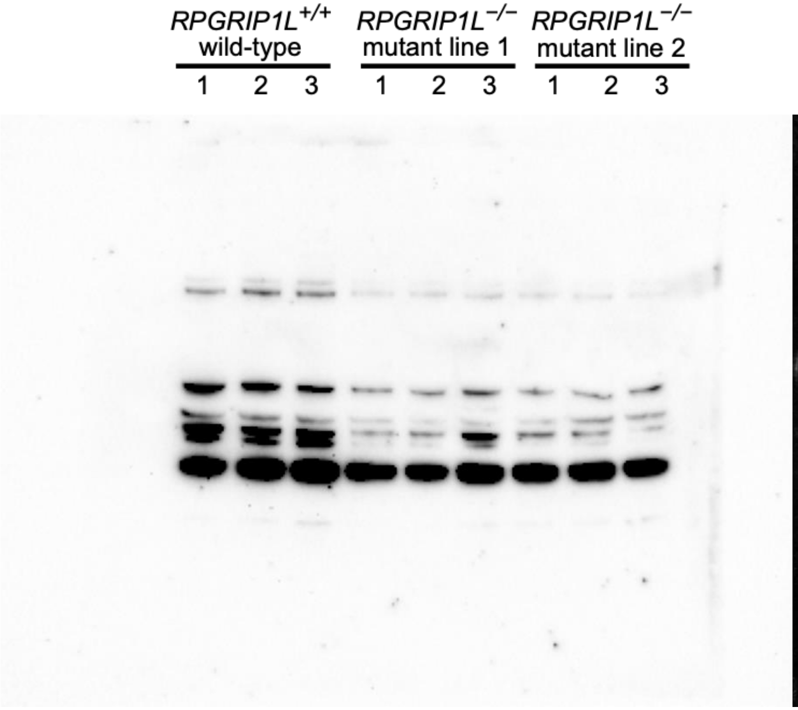

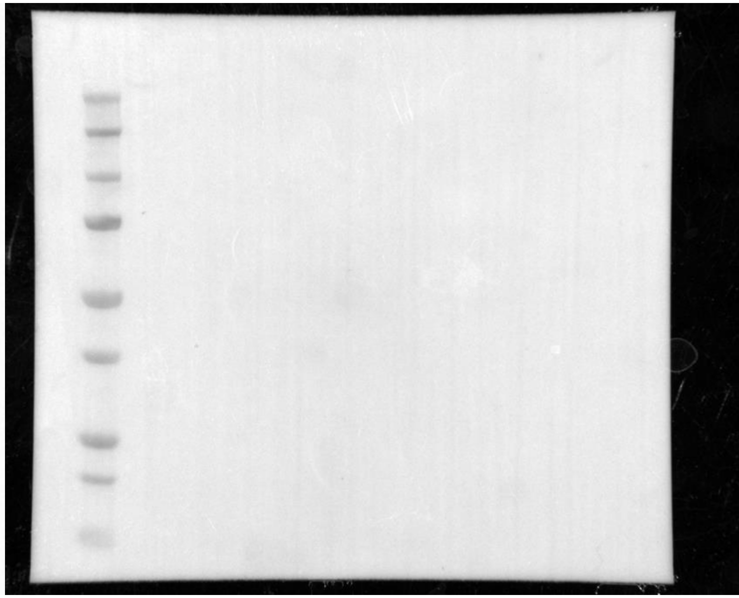

Probed with antibody to RPGRIP1L

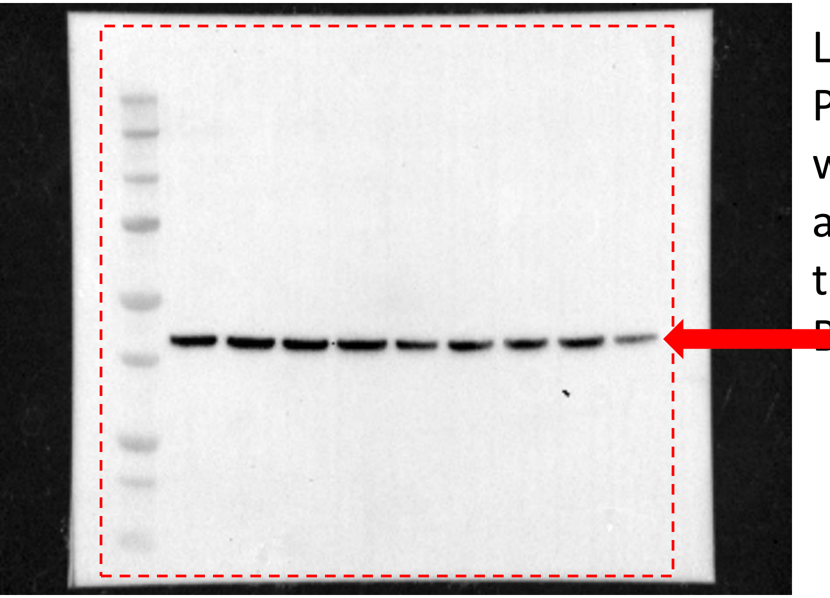

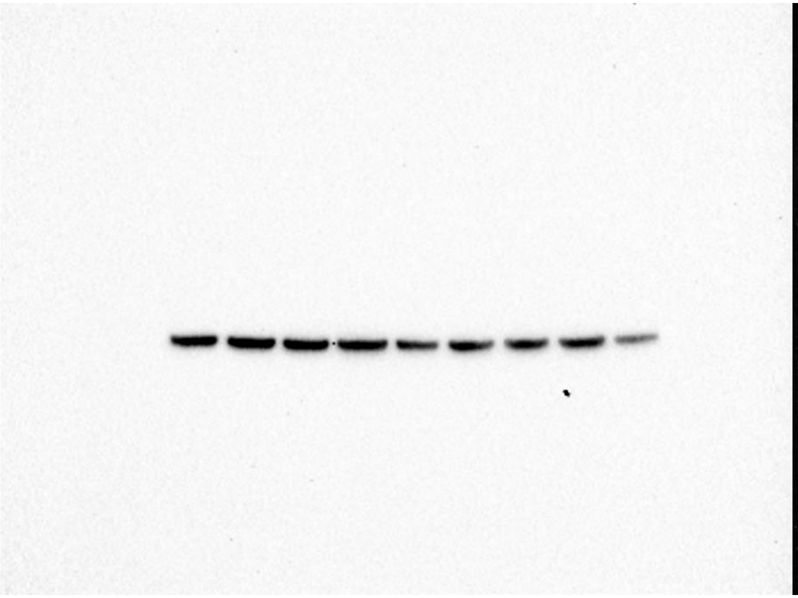

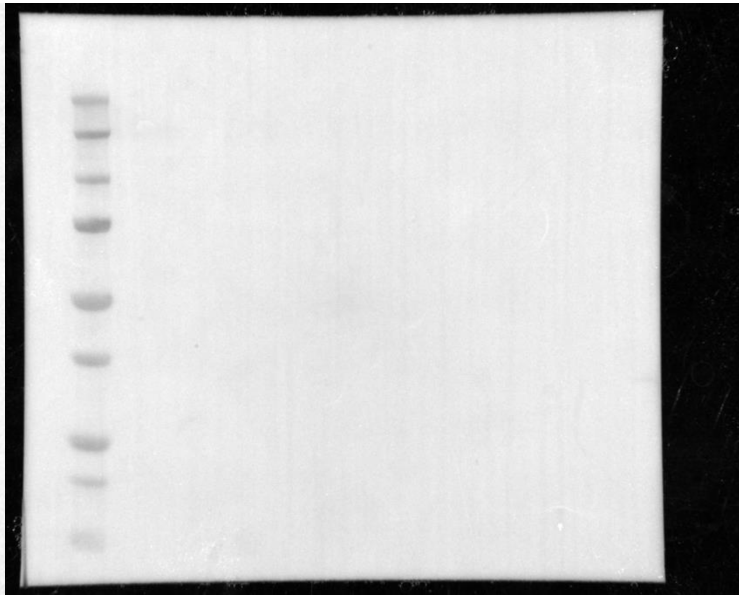

Loading Probed with antibody to Beta actin

Full unedited blot for Supplemental Figure 7d

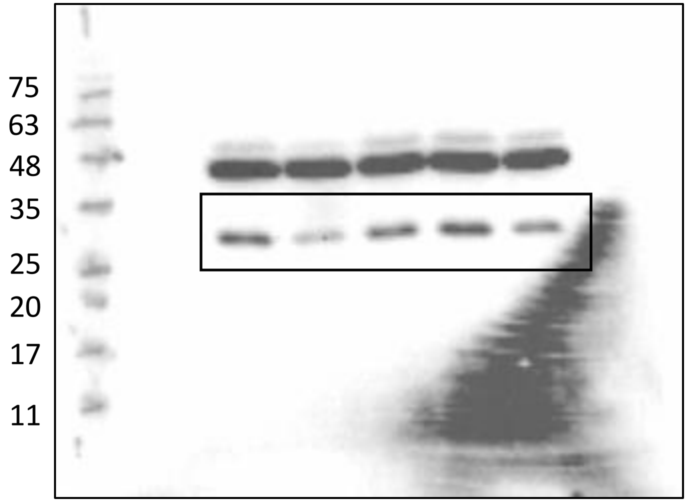

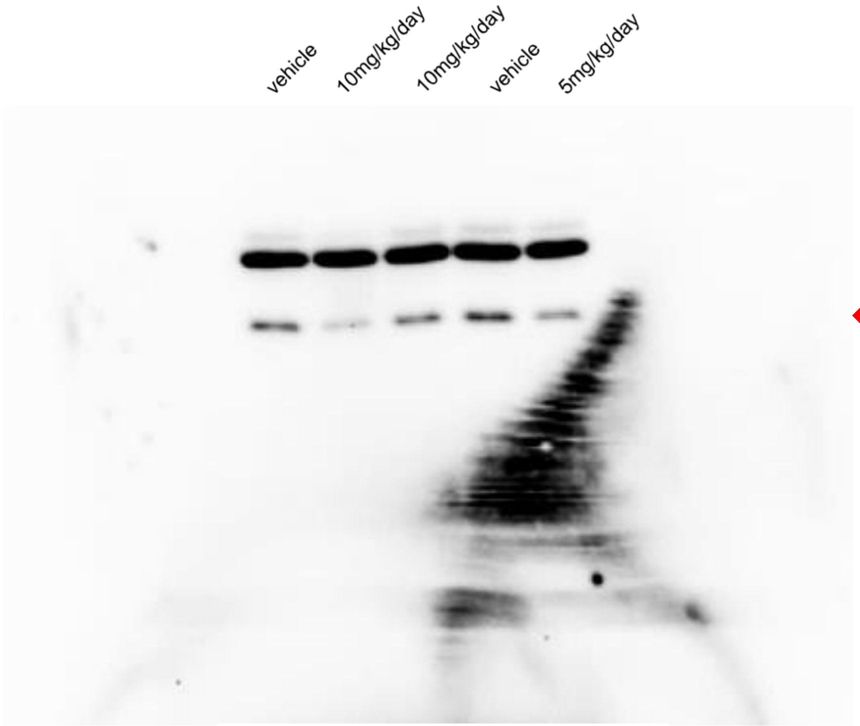

Probed with antibody to p-MLCII

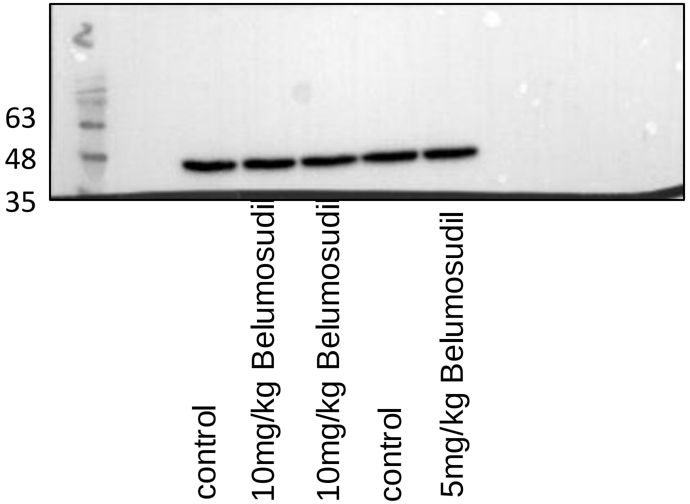

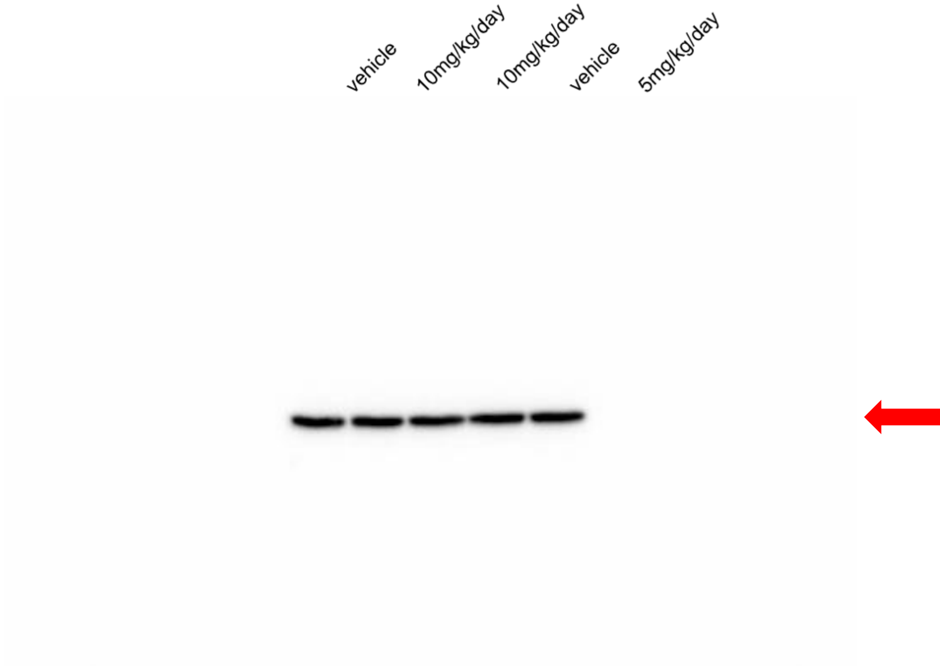

Loading Probed with antibody to beta actin
