# Supplementary material for: Drug and siRNA screens identify ROCK2 as a therapeutic target for ciliopathies
Source: Commun Med (Lond). 2025 Apr 19;5:129. doi: 10.1038/s43856-025-00847-1 (PMC12009310; doi:10.1038/s43856-025-00847-1)
Supplement: Supplementary file 9 — reporting summary [file 43856_2025_847_MOESM9_ESM.pdf]

nature portfolio

Corresponding author(s): Colin A Johnson

Last updated by author(s): Dec 18, 2024

Reporting Summary

Nature Portfolio wishes to improve the reproducibility of the work that we publish. This form provides structure for consistency and transparency in reporting. For further information on Nature Portfolio policies, see our Editorial Policies and the Editorial Policy Checklist.

Statistics

For all statistical analyses, confirm that the following items are present in the figure legend, table legend, main text, or Methods section.

n/a	Confirmed	
☐	☒	The exact sample size (*n*) for each experimental group/condition, given as a discrete number and unit of measurement
☐	☒	A statement on whether measurements were taken from distinct samples or whether the same sample was measured repeatedly
☐	☒	The statistical test(s) used AND whether they are one- or two-sided<br>*Only common tests should be described solely by name; describe more complex techniques in the Methods section.*
☐	☒	A description of all covariates tested
☐	☒	A description of any assumptions or corrections, such as tests of normality and adjustment for multiple comparisons
☐	☒	A full description of the statistical parameters including central tendency (e.g. means) or other basic estimates (e.g. regression coefficient) AND variation (e.g. standard deviation) or associated estimates of uncertainty (e.g. confidence intervals)
☐	☒	For null hypothesis testing, the test statistic (e.g. F, t, r) with confidence intervals, effect sizes, degrees of freedom and P value noted<br>*Give P values as exact values whenever suitable.*
☒	☐	For Bayesian analysis, information on the choice of priors and Markov chain Monte Carlo settings
☒	☐	For hierarchical and complex designs, identification of the appropriate level for tests and full reporting of outcomes
☒	☐	Estimates of effect sizes (e.g. Cohen's *d*, Pearson's *r*), indicating how they were calculated

Our web collection on statistics for biologists contains articles on many of the points above.

Software and code

Policy information about availability of computer code

Data collection	source code available in Supplemental File 3
Data analysis	not applicable

For manuscripts utilizing custom algorithms or software that are central to the research but not yet described in published literature, software must be made available to editors and reviewers. We strongly encourage code deposition in a community repository (e.g. GitHub). See the Nature Portfolio guidelines for submitting code & software for further information.

Data

Policy information about availability of data

All manuscripts must include a data availability statement. This statement should provide the following information, where applicable:

- Accession codes, unique identifiers, or web links for publicly available datasets
- A description of any restrictions on data availability
- For clinical datasets or third party data, please ensure that the statement adheres to our policy

CRISPR-Cas9 edited cell lines are available upon request. All raw experimental data is available in Supplemental File 4.

Human research participants

Policy information about studies involving human research participants and Sex and Gender in Research.

Reporting on sex and gender	no human research participants
Population characteristics	*Describe the covariate-relevant population characteristics of the human research participants (e.g. age, genotypic information, past and current diagnosis and treatment categories). If you filled out the behavioural & social sciences study design questions and have nothing to add here, write "See above."*
Recruitment	*Describe how participants were recruited. Outline any potential self-selection bias or other biases that may be present and how these are likely to impact results.*
Ethics oversight	*Identify the organization(s) that approved the study protocol.*

Note that full information on the approval of the study protocol must also be provided in the manuscript.

Field-specific reporting

Please select the one below that is the best fit for your research. If you are not sure, read the appropriate sections before making your selection.

☒ Life sciences ☐ Behavioural & social sciences ☐ Ecological, evolutionary & environmental sciences

For a reference copy of the document with all sections, see nature.com/documents/nr-reporting-summary-flat.pdf

Life sciences study design

All studies must disclose on these points even when the disclosure is negative.

Sample size	Sample size is determined empirically, depending on the biological assay in question, or otherwise by power calculation where appropriate.
Data exclusions	Data exclusions are detailed in the main text of the manuscript and comprise singleton outliers in high content screening datasets, excluded on the basis of manual inspection of images (eg cell loss in a field of view, incorrect focus).
Replication	All experiments were subject to three biological replicates, each comprising technical replicates as detailed in the main text of the manuscript, other than two biological replicates ("runs") of the whole genome siRNA and drug screens.
Randomization	Cell-lines and animals were allocated on the basis of siRNA or drug treatment, or on genotype, into experimental groups versus control groups (eg isogenic-matched iPSC lines)
Blinding	Analysis of images in Figure 6 (genotype, treatment status) was blinded to three independent scorers. Data was unblinded and collated by first author CELS.

Reporting for specific materials, systems and methods

We require information from authors about some types of materials, experimental systems and methods used in many studies. Here, indicate whether each material, system or method listed is relevant to your study. If you are not sure if a list item applies to your research, read the appropriate section before selecting a response.

Materials & experimental systems

n/a	Involved in the study	
☐	☒	Antibodies
☐	☒	Eukaryotic cell lines
☒	☐	Palaeontology and archaeology
☐	☒	Animals and other organisms
☒	☐	Clinical data
☒	☐	Dual use research of concern

Methods

n/a	Involved in the study	
☒	☐	ChIP-seq
☒	☐	Flow cytometry
☒	☐	MRI-based neuroimaging

Antibodies

Antibodies used	Details of all antibodies used are provided in the Methods & Materials section of the manuscript.

Validation	All antibodies are commercially supplied and were only used if a validation statement of specificity was available from the manufacturer or supplier (ie specificity confirmed by siRNA knockdown, loss of signal on knockout cell or tissues, peptide block experiments, or immunopreciptation experiment of the cognate protein).

Eukaryotic cell lines

Policy information about cell lines and Sex and Gender in Research

Cell line source(s)	Cell-lines were obtained from ECACC with certificates of validation and expanded for use from early passage cultures. The cell line OX161/c1 was immortalised from tubule cells taken from an ADPKD human kidney as part of clinical care with informed consent and have been previously described in Lannoy et al. Kidney Int 2020;98:404-19.
Authentication	The wildtype iPSC line has been described and validated as described in Atkinson et al. Nat Commun 2024;15:3138. doi: 10.1038/s41467-024-47253-0
Mycoplasma contamination	All lines were tested using a commercial ELISA test for mycoplasma contamination; all lines reported in the manuscript are negative.
Commonly misidentified lines (See ICLAC register)	Not applicable.

Animals and other research organisms

Policy information about studies involving animals; ARRIVE guidelines recommended for reporting animal research, and Sex and Gender in Research

Laboratory animals	The tetracycline-inducible, kidney-specific Pkd1 mice model (Pax8rtTA-TetO-Cre-Pkd1fl/fl) was used at postnatal days (PNs) 13-15, as described previously in Streets et al. JCI Insight. 2020;5:e135385
Wild animals	Not applicable
Reporting on sex	Sex of animals was allocated randomly to control or treatment groups in data reported in Figure 6.
Field-collected samples	Not applicable
Ethics oversight	All experiments in mice were carried out under project licence PP9820851 with local ethical approval at the University of Sheffield, UK.

Note that full information on the approval of the study protocol must also be provided in the manuscript.
